# Cycloaddition reactions of heterocyclic azides with 2-cyanoacetamidines as a new route to *C*,*N*-diheteroarylcarbamidines

**DOI:** 10.3762/bjoc.20.3

**Published:** 2024-01-05

**Authors:** Pavel S Silaichev, Tetyana V Beryozkina, Vsevolod V Melekhin, Valeriy O Filimonov, Andrey N Maslivets, Vladimir G Ilkin, Wim Dehaen, Vasiliy A Bakulev

**Affiliations:** 1 Department of Organic Chemistry, Perm State University, 15 Bukireva st., Perm 614990, Russia; 2 TOS Department, Ural Federal University, 19 Mira st., Yekaterinburg 620002, Russia; 3 Innovation Center for Chemical and Pharmaceutical Technologies, Ural Federal University, 19 Mira st., Yekaterinburg 620002, Russia; 4 Department of Medical Biology and Genetics, Ural State Medical University, 3 Repina st., Yekaterinburg 620028, Russian; 5 Sustainable Chemistry for Metals and Molecules, Department of Chemistry, KU Leuven, Celestijnenlaan 200F, B-3001 Leuven, Belgium

**Keywords:** Cornforth rearrangement, cycloaddition reactions, 3,3-diaminoacrylonitriles, heterocyclic azides, 1,2,3-triazole

## Abstract

A novel and efficient base-catalyzed, transition-metal-free method for the synthesis of diheterocyclic compounds connected by an amidine linker, including apart from the common 1,2,3-triazole ring, either an additional pyrimidinedione, 4-nitroimidazole, isoxazole, 1,3,4-triazole, 2-oxochromone or thiazole ring, has been developed. The process was facilitated by a strong base and includes the cycloaddition reaction of 3,3-diaminoacrylonitriles (2-cyanoacetamidines) to heterocyclic azides followed by a Cornforth-type rearrangement to the final products. The reaction is tolerant to various *N*-monosubstituted 3,3-diaminoacrylonitriles and to different heterocyclic azides. The developed method has a broad scope and can be applied to obtain a variety of *N*-heteroaryl-1,2,3-triazole-4-carbimidamides with alkyl, allyl, propargyl, benzyl, cycloalkyl, and indolyl substituents at the *N*^1^ position .

## Introduction

Heteroaryl amidines are widely used in the synthesis of various nitrogen-containing heterocyclic compounds and have a variety of biological activities [1–4]. After the discovery of click chemistry [5–6] involving the CuAAC method of 1,2,3-triazole synthesis [7–8], there has been great interest of studing the chemical and biological properties of the triazoles thus obtained [9–12]. It should be noted that the synthesis of amidines containing other heterocycles in addition to 1,2,3-triazole in the molecule has not been described in the literature. In this regard, it is of interest to develop an effective method for the synthesis of hybrids of 1,2,3-triazole with other heterocycles and to identify biologically active compounds among the synthesized compounds.

**Scheme 1:**
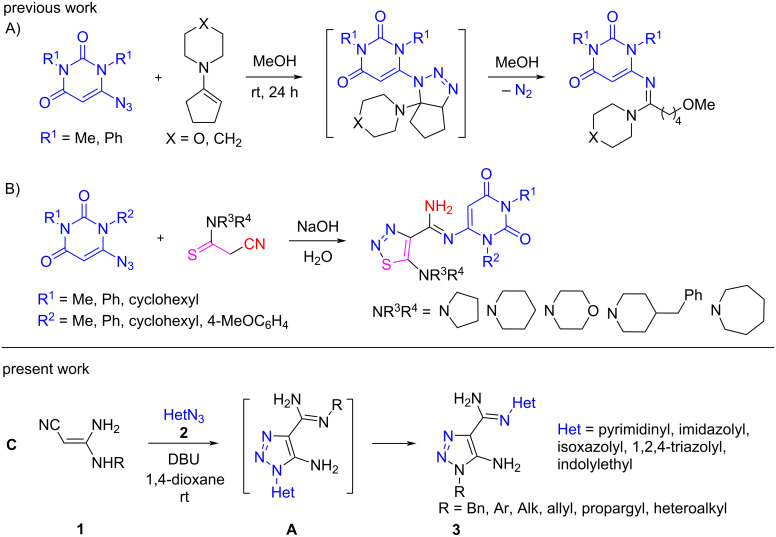
Synthesis of heteroaryl amidines.

It is known that the cycloaddition reaction of azidopyrimidinediones with enamines [13] represents an effective method for the synthesis of pyrimidinyl amidines [14] (Scheme 1A). A few years ago, we discovered that the replacement of enamines with 2-cyano-*N*,*N*-dialkylethanethioamides in this reaction [15] led to the formation of amidines containing two heterocycles: pyrimidine-2,4-dione and 1,2,3-thiadiazole (Scheme 1B).

The idea came to our mind to construct a system comprising pyrimidine and 1,2,3-triazole rings connected with an amidine linker by the cycloaddition reaction of 3,3-diaminoacrylonitriles with 6-azidopyrimidine (Scheme 1C). Based on this idea, an efficient and novel method with wide scope has been developed which was then applied to obtain a variety of previously unknown 5-amino-1-substituted 1,2,3-triazole-*N*-heteroaryl-4-carboximidamides **3** bearing alkyl, allyl, propargyl, benzyl, cycloalkyl, and various heteroaryl substituents at the *N*^1^ postion of the carbimidamide group. Herein, we disclose our results on the cycloaddition reaction of 3,3-diaminoacrylonitriles **1** with heteroaryl azides (HetN_3_) **2** [16] leading to 5-amino-1,2,3-triazole-4-*N*-heteroarylcarbimidamides **3** (Scheme 1C).

## Results and Discussion

### Optimization of the reaction of amidine **1a** with azide **2a**

We initiated the study by investigating a model reaction involving the cycloaddition of 3-amino-3-(benzylamino)acrylonitrile (**1a**) to 6-azidopyrimidine-2,4-dione **2a** (Table 1). To our surprise we obtained *(Z)*-5-amino-1-benzyl-*N'*-(1,3-dimethyl-2,6-dioxo-1,2,3,6-tetrahydropyrimidin-4-yl)-1*H-*1,2,3-triazole-4-carboximidamide (**3a**) in 93% yield as the major product with 5-amino-1-benzyl-1*H*-1,2,3-triazole-4-carbonitrile (**4**) in 5% yield when the reaction was carried out at room temperature in 1,4-dioxane in the presence of an equivalent amount of TEA (Table 1, entry 1). This result is in contrast to our previous findings where the reaction of compound **1a** with sulfonyl azides led to 5-amino-1,2,3-triazole-4-*N*-sulfonylamidines selectively [17].

**Table 1:** Optimization of the reaction of amidine **1a** with 6-azidopyrimidine-2,4-dione **2a**.^a^

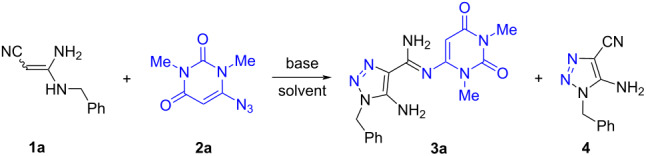					

Entry	Solvent	Base (mol %)	*Т*	Ratio **3**:**4** (%)	Yield triazole **3a** (%)^b^

1	1,4-dioxane	Et_3_N (100)	rt	95:5	93
2	EtOH	DBU (100)	rt	91:9	87
3	DCM	DBU (100)	rt	90:10	83
4	1,4-dioxane	DBU (100)	rt	99:1	95
5	1,4-dioxane	DBU (100)	rt	100:0	98^c^
6	1,4-dioxane	DBU (80)	rt	90:10	85
7	1,4-dioxane	DBU (120)	rt	100:0	98
8	1,4-dioxane	no base	rt	40:60	38
9	1,4-dioxane	pyridine (100)	rt	37:63	35
10	EtOH	NaOH (100)	rt	94:6	90
11	EtOH	NaOH (100)	0 °С	97:3	95^c^

^a^Conditions: **1a** (0.5 mmol), **2a** (0.5 mmol), solvent (2 mL), 5–10 min; ^b^determined by ^1^H NMR spectroscopy; ^c^isolated yield.

The following screening of organic (Table 1, entries 2‒7 and 8) and inorganic (Table 1, entry 10) bases at room temperature revealed that using DBU resulted in the highest yield of triazole **3a**. Analysis of experiments with 100 mol %, 120 mol %, and 80 mol % of DBU (Table 1, entries 2‒7) showed that the use of 100 mol % of DBU is optimal for the selective synthesis of triazole **3a** in high yield (Table 1, entries 4 and 5). A study of the reaction medium revealed that common organic solvents are highly efficient for this cascade reaction (Table 1, entries 1‒11). Among the solvents screened, 1,4-dioxane was found the best solvent in terms of yield of the target product, solubility of the reagents, and ease of separation of the product.

Thus, the optimal conditions found were reacting amidine **1** with azide **2** in the presence of DBU in a 1:1:1 ratio in 1,4-dioxane at room temperature. A similar yield of the target product **3a** was obtained by the reaction in ethanol in the presence of NaOH at 0 °С (entry 11 in Table 1). The latter conditions can be an alternative for the synthesis of compounds **3**.

### Synthesis of *N*-heteroaryl amidines **3**

**Figure 1:**
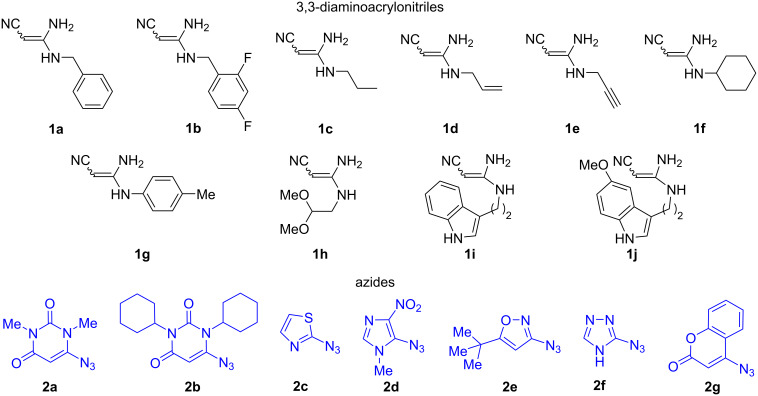
Structures of starting compounds.

We hypothesized that both 3,3-diaminoacrylonitriles **1** and azides **2** can serve as three atom building blocks (Figure 1), affording 5-amino-1,2,3-triazole-4-carbimidamides **A** (Scheme 1C), which could be suitable precursors for the desired compounds **3**.

**Scheme 2:**
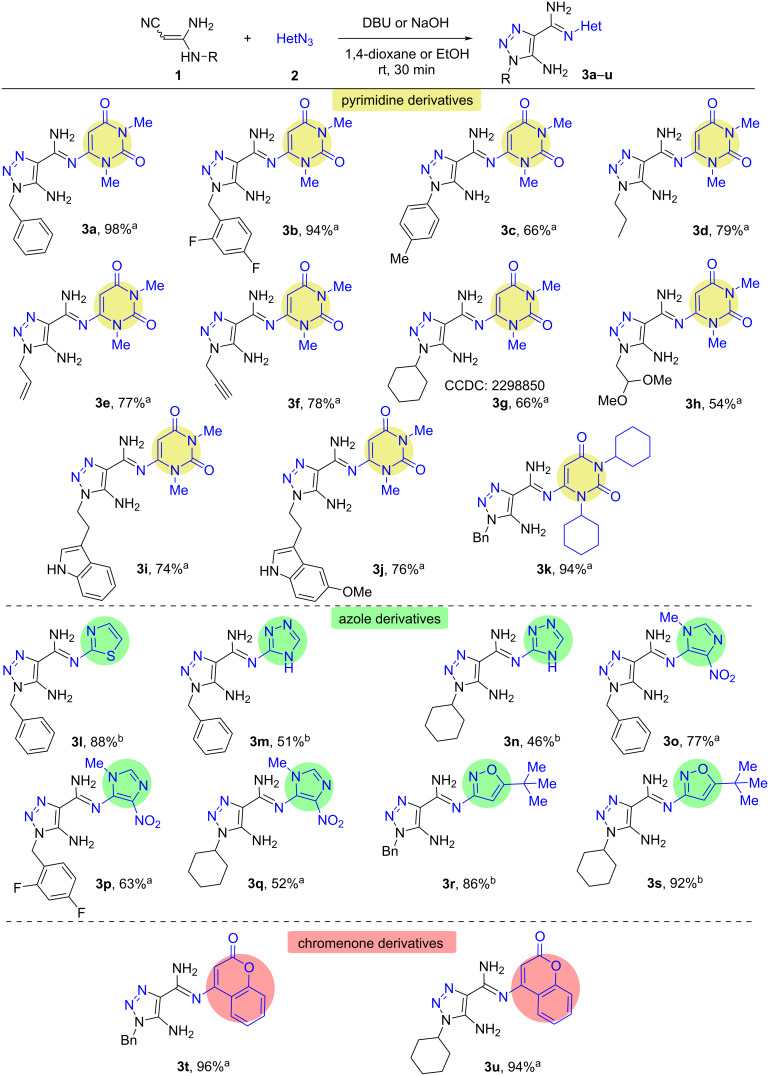
Scope of 3,3-diaminoacrylonitriles **1** and heterocyclic azides **2**. Reaction conditions: **1** (0.5 mmol), **2** (0.5 mmol), DBU (0.5 mmol), 1,4-dioxane (2 mL), rt, 30 min (method A)^a^; **1** (0.5 mmol), **2** (0.5 mmol), NaOH (0.5 mmol), EtOH (2 mL), 0 °C → rt, 30 min (method B)^b^.

With the starting 3,3-diaminoacrylonitriles **1** and azides **2** at hands they were subjected to the optimized reaction conditions to obtain the desired 5-amino-1,2,3-triazole-4-*N*-heteroarylcarbimidamides **3**. In this way, 21 pyrimidine (**3a**–**k**), azolyl (**3l**–**s**), and chromenone (**3t** and **3u**) derivatives were successfully prepared (Scheme 2 and Supporting Information File 1).

Generally, the reaction proceeded in moderate to high yields (46–98%), ranging between 79–98% for reactions of 3,3-diaminoacrylonitriles **1** with azidoheterocycles **2**. Compared with azidopyrimidines **2a**,**b** and azidochromene **2g**, the use of azidoazoles **2c**–**f** delivered the expected amidines **3l**–**r** in somewhat lower yields. No definite electronic effect of the substituents in azides **2** was observed. We assume the higher yield of pyrimidine containing triazoles **3a–k** by their lower solubility in 1,4-dioxane compared with the solubility of azole containing compounds **3l–s** in both 1,4-dioxane and ethanol.

With regard to the scope of diaminoacrylonitriles **1a**–**j**, various substituents at the nitrogen were tolerated including aryl, (substituted) benzyl and alkyl substituents (including tryptamine, cyclohexyl, propargyl, and allyl) and furnished the *N*-heteroaryl amidines **3a**–**u** in high or moderate yields (46–98%) (Scheme 2). There was no apparent substituent effect on the yield of the final compounds **3** observed.

The structures of compounds **3a**–**u** were confirmed by IR, ^1^H and ^13^C NMR spectroscopy (Figures S1‒S44 in Supporting Information File 1) as well as by high-resolution mass spectrometry (HRMS). X-ray data obtained for compound **3g** gave us final proof of the structure of the prepared compounds.

**Scheme 3:**
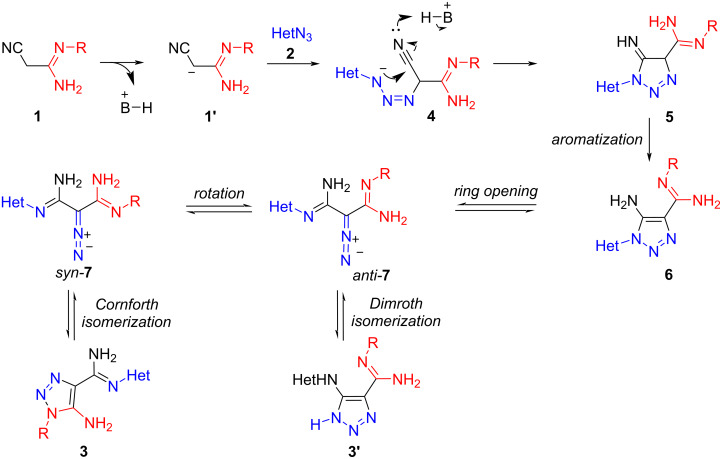
Proposed mechanism for the formation of triazoles **3**.

To explain the outcome of the tandem reaction of 3,3-diaminoacrylonitriles to heterocyclic azides, a tentative mechanism for the formation of 1,2,3-triazoles **3** from acrylonitriles **1** and azides **2** is shown in Scheme 3.

Firstly, treatment with a base, leads to deprotonation of acrylonitriles **1** to form anion **1′**. The subsequent formal cycloaddition of anion **1**′ with azides **2** then affords triazolines **5**, which aromatize through a 1,3-H-shift to afford the triazoles **6**. Two pathways towards the isomeric triazoles **3** can be proposed: the first involving the electrocyclic ring opening of triazole **6** to form diazo compound *anti***-7**, followed by rotation around the C‒C bond of the amidine group to furnish rotamer *syn*-**7** which then undergoes 1,5-dipolar cyclization to products **3**. The second path involves a Dimroth-type cyclization to form products **3′**, which however, were not experimentally observed.

The base-catalyzed cycloaddition of 3,3-diaminoacrylonitriles **1** to azides **2**, thus proceeds in a Cornforth-type fashion, which involves a triazole–triazole isomerization through ring opening, rotation of the amidine substituent around the single bond, and cyclization.

### Cytotoxic activity

The cytotoxic activity of seven synthesized compounds **3b**,**d**,**e**,**g**,**h**,**l**,**m** was studied. According to the results of the MTT test, IC_50_ values were calculated for these compounds (Table 2). The results obtained indicated that compounds **3b**,**e**,**g**,**h** did not have a pronounced effect on the viability of cultured cells within the concentration range. This indicates the low cytotoxicity of these compounds. Meanwhile, compounds **3m**,**l** are characterized by a borderline inhibition of cell culture viability. Attention is drawn to the increased toxic effect of **3m** on cells of tumor origin, in comparison with normal human embryonic cells. Identification of the cause of the registered selective toxicity of the tested compounds requires further study.

**Table 2:** Cytotoxicity index (IC_50_ ± SE) in µM of the studied compounds on human embryo kidney cells (HEK-293), glioblastoma (A-172), and osteosarcoma (HOS) cells.

Entry	Compound	Type of cell studied		

		НЕK-293	A-172	HOS

1	**3b**	>256	>256	>256
2	**3d**	>1024	>1024	>1024
3	**3e**	>1024	>1024	>1024
4	**3g**	>512	>512	>512
5	**3h**	471.98 ± 106.54	>512	>512
6	**3l**	174.62 ± 43.72	>256	224.28 ± 67.36
7	**3m**	184.60 ± 51.06	95.71 ± 13.29	83.86 ± 17.13
8	cisplatin	59.90 ± 19.97	39.51 ± 6.39	38.14 ± 5.93

## Conclusion

Thus, we have introduced an effective base-catalyzed tandem reaction including a Cornforth-type rearrangement of 1-heteroaryl-1,2,3-triazole-4-carboximidamides and formal cycloaddition reaction of readily available heterocyclic azides with 3,3-diaminoacrylonitriles. The reaction represents a novel method for the preparation of 1,2,3-triazoles bearing an *N*-hetaryl amidine moiety and thus this reaction offers a novel method for the preparation of new types of 1-substituted-1,2,3-triazoles, widening the synthetic applications of both azides and derivatives of acrylonitrile. Some of the prepared compounds exhibited a mild toxic effect on tumor cells in comparison with normal human embryonic cells.

## Experimental

3,3-Diaminoacrylonitriles **1b** and **1f** were synthesized from ethyl 2-cyanoacetimidate and corresponding amines according to the literature procedure [17] and compounds **1a**,**c**–**e**,**h**–**j** are commercially available. Azides **2a**–**d**,**f**,**g** were synthesized according to the literature procedures [18–23], and azide **2e** is commercially available.

### Preparation of triazoles **3**

Method A. In a manner similar to [17], DBU (0.5 mmol) was added to the solution of amidine **1** (0.5 mmol) in 1,4-dioxane (2 mL) at room temperature and azide **2** (0.5 mmol) was added to the resulting solution 5 min later. The reaction mixture was stirred for 30 min at room temperature, then water (6 mL) was added and the resulting solution was stirred for additional 5 min. Then, acetic acid (34 µL) was added to the reaction mixture, the formed precipitate was filtered off, washed with water, and dried in a desiccator over P_4_O_10_.

Method B. Amidine **1** (0.5 mmol) was added into a solution of sodium hydroxide, freshly prepared from sodium hydroxide (20 mg, 0.5 mmol) and ethanol (2 mL), and the resulting mixture was stirred at room temperature for 5–10 min. Then, the mixture was cooled to 0 °C, the corresponding azide **2** (0.5 mmol) was added and the resulting mixture were stirred for 30 min, after which cooling was removed. The reaction mixture was allowed to warm to ambient temperature under stirring and water (8 mL) was added to the mixture. The resulting solution was stirred for 5 min, after which acetic acid (34 µL) was added. The formed precipitate was filtered off, washed with water, and dried in a desiccator over P_4_O_10_.

*(****Z*)-5-Amino-1-benzyl-*N*'-(1,3-dimethyl-2,6-dioxo-1,2,3,6-tetrahydropyrimidin-4-yl)-1*H*-1,2,3-triazole-4-carboximidamide (3a).** Compound **3a** was obtained in 98% yield (174 mg) according to the general procedure A (DBU: 76 mg, 75 µL, 0.5 mmol; amidine **1a**: 86 mg, 0.5 mmol; azide **2a**: 90 mg, 0.5 mmol; 1,4-dioxane (2 mL)) as a colorless powder; mp 225–226 °C; ^1^H NMR (400 MHz, DMSO-*d*_6_) δ 3.16 (s, 3H), 3.21 (s, 3H), 5.08 (s, 1H), 5.46 (s, 2H), 6.52 (s, 1H), 6.53 (s, 1H), 7.19 (br. s, 2H), 7.25–7.39 (m, 5H); ^13^C NMR (101 MHz, DMSO-*d*_6_) δ 27.1, 29.8, 48.4, 87.4, 120.9, 127.4, 127.6, 128.5, 135.7, 143.8, 152.3, 152.9, 157.3, 162.4; IR (ATR, KBr, cm^−1^): ν 3402, 3316, 3201, 1700, 1688, 1649, 1629, 1594, 1568, 1520, 1497, 1476, 1454, 1444, 1426, 1386, 1358, 1335, 1311, 1276, 1264, 1248, 1194, 1090, 1057, 1028; HRMS–ESI-TOF (*m/z*): [M + H]^+^ calcd for C_16_H_19_N_8_O_2_^+^, 355.1625; found: 355.1628.

**(*Z*)-5-Amino-1-benzyl-*N*'-(thiazol-2-yl)-1*H*-1,2,3-triazole-4-carboximidamide (3l).** Compound **3l** was obtained in 88% yield (117 mg) according to the general procedure B (NaOH: 20 mg, 0.5 mmol; amidine **1a**: 77 mg, 0.5 mmol; azide **2c**: 56 mg, 0.5 mmol; ethanol (2 mL)) as light yellow needles; mp 199–200 °C; ^1^H NMR (400 MHz, DMSO-*d*_6_) δ 5.48 (s, 2H), 6.68 (s, 1H), 6.69 (s, 1H), 7.10 (d, *J =* 3.9 Hz, 1H), 7.24 (d, *J* = 6.9 Hz, 2H), 7.28–7.38 (m, 3H), 7.43 (d, *J* = 3.9 Hz, 1H), 8.42 (br. s, 1H), 9.15 (br. s, 1H); ^13^C NMR (101 MHz, DMSO-*d*_6_) δ 48.5, 112.6, 121.2, 127.2, 127.6, 128.5, 135.7, 138.8, 143.6, 153.5, 174.5; IR (ATR, KBr, cm^−1^): ν 3400, 3359, 3255, 1625, 1602, 1563, 1554, 1508, 1495, 1483, 1455, 1436, 1425, 1402, 1385, 1356, 1332, 1319, 1303, 1283, 1257, 1215, 1151, 1095, 1067, 1053, 1035, 1011; HRMS–ESI-TOF (*m/z*): [M + H]^+^ calcd for C_13_H_14_N_7_S^+^, 300.1026; found, 300.1031.

### X-ray structure determination

**3g**: Crystal data for C_15_H_22_N_8_O_2_ (M = 346.40 g/mol): monoclinic, space group *P*2_1_/*c* (no. 14), *a* = 14.734(3) Å, *b* = 9.000(2) Å, *c* = 13.125(4) Å, β = 104.29°, *V* = 1686.6(8)Å^3^, Z = 4, *T* = 295(2) K, μ(Mo Kα) = 0.097 mm^−1^, *D*_calc_ = 1.364 g/cm^3^, 6415 reflections measured (5.4° ≤ 2Θ ≤ 59°), 6415 unique (R(sigma) = 0.0978) which were used in all calculations. The final R_1_ was 0.0693 (I > 2σ(I)) and wR_2_ was 0.1849 (all data). The refined twin ratio was 0.6748(18):0.3252(18).

The experiment was accomplished on the automated X-ray diffractometer «Xcalibur Ruby» with CCD detector following standard procedures (Mo Kα irradiation, graphite monochromator, ω-scans with 1° step at *T* = 295(2) K). Empirical absorption correction was applied. The structure was solved using the intrinsic phases in ShelXT program [24] and refined by ShelXL [25] using full-matrix least-squared method for non-hydrogen atoms. The H-atoms atoms bound to carbon were placed in the calculated positions and were refined in isotropic approximation. The hydrogen atoms of NH_2_ groups were refined independently with isotropic displacement parameters. The solution and refinement of the structures were accomplished with the Olex2 program package [26]. The structure was refined using HKLF5 format file as twin with two components.

CCDC 2298850 (**3g**) contains the supplementary crystallographic data for this paper. These data can be obtained free of charge via http://www.ccdc.cam.ac.uk/conts/retrieving.html (or from the CCDC, 12 Union Road, Cambridge CB2 1EZ, UK; Fax: +44 1223 336033; Email: deposit@ccdc.cam.ac.uk)

## Supporting Information

File 1 Full experimental details and characterization data of all new compounds.

File 2 Copies of NMR spectra of all new compounds.

## Data Availability

Data generated and analyzed during this study is openly available in CCDC at https://doi.org/10.5517/ccdc.csd.cc2h54g9.
