# Supplementary material for: Cycloaddition reactions of heterocyclic azides with 2-cyanoacetamidines as a new route to C,N-diheteroarylcarbamidines
Source: Beilstein J Org Chem. 2024 Jan 5;20:17–24. doi: 10.3762/bjoc.20.3 (PMC10783006; doi:10.3762/bjoc.20.3)
Supplement: File 1 — Full experimental details and characterization data of all new compounds. [file Beilstein_J_Org_Chem-20-17-s001.pdf]

BEILSTEIN JOURNAL OF ORGANIC CHEMISTRY

Supporting Information

for

Cycloaddition reactions of heterocyclic azides with 2-cyanoacetamidines as a new route to *C*,*N*-diheteroarylcarbamidines

Pavel S. Silaichev, Tetyana V. Beryozkina, Vsevolod V. Melekhin, Valeriy O. Filimonov, Andrey N. Maslivets, Vladimir G. Ilkin, Wim Dehaen and Vasiliy A. Bakulev

Beilstein J. Org. Chem. **2024,** *20,* 17–24. doi:10.3762/bjoc.20.3

Full experimental details and characterization data of all new compounds

License and Terms: This is a supporting information file under the terms of the Creative Commons Attribution License (https://creativecommons.org/licenses/by/4.0). Please note that the reuse, redistribution and reproduction in particular requires that the author(s) and source are credited and that individual graphics may be subject to special legal provisions.
The license is subject to the *Beilstein Journal of Organic Chemistry* terms and conditions: (https://www.beilstein-journals.org/bjoc/terms)

Experimental Part

All chemicals were used without further purification. The reaction progress was determined on the Waters UPLC Acquity I-Class ultra-high-efficiency chromatography system with a tandem quadrupole mass spectrometric XEVO TQD detector and a diode-matrix detector. Analytical thin layer chromatography was performed on aluminum foil plates Sorbfil UV-254 coated with 0.2 mm silica gel and visualized with UV-lamp 254 nm in an EtOAc/petroleum ether (PE) system (3:1, 2:1 or 1:2). Melting points were determined on a melting point apparatus Stuart SMP10 (Staffordshire, ST15 OSA, UK) and are uncorrected. All NMR spectra were recorded with a Bruker Avance II (Karlsruhe, Germany) spectrometer at 400 MHz, 600 MHz (^{1}H NMR), 101 MHz (^{13}C NMR) and ^{19}F NMR (377 MHz) in DMSO–d_6. The chemical shifts are given in ppm relative to the resonance of the solvent [^{1}H: δ (DMSO–d_5) = 2.50, ^{13}C: δ (DMSO–d_6) = 39.52 ppm]. Multiplicities were given as: s (singlet); br. s (broad singlet); d (doublet); t (triplet); dd (double of doublet); (m (multiplet). Coupling constants are reported as *J* value in Hz. High-resolution mass spectra (HRMS) were recorded using ultrahigh resolution quadrupole time-of-flight mass spectrometer Bruker maXis impact HD (USA) with the electrospray ionization probe installed coupled with Agilent 1260 HPLC system. The Fourier transform infrared (FT-IR) spectra were obtained using a Bruker Alpha (ATR, ZnSe) spectrometer (Ettlingen, Germany) in the 4000–500 cm^{-1} region.

The cytotoxic activity studies were carried out on cultured embryo kidney cells (HEK293, ATCC CRL 1573), glioblastoma (A-172, ATCC CRL 1620) and human osteosarcoma (HOS, ATCC CRL 1543). Cultures were obtained from the CCU "Collection of Vertebrate Cell Cultures" (Institute of Cytology of the Russian Academy of Sciences, St. Petersburg, Russia). The cells were cultured using a DMEM/F12 medium containing 10% fetal bovine serum at 37°C, 5% CO_2 and 98% humidity. Subcultivation using a 0.25% trypsin solution was carried out when the culture reached ≥90% confluence.

(Z)-5-Amino-1-(2,4-difluorobenzyl)-N'-(1,3-dimethyl-2,6-dioxo-1,2,3,6-tetrahydropyrimidin-4-yl)-1H-1,2,3-triazole-4-carboximidamide (**3b**). Compound **3b** was obtained in 94% yield (184 mg) according to the general procedure A (DBU: 76 mg, 75 µL, 0.5 mmol; amidine **1b**: 104 mg, 0.5 mmol; azide **2a**: 90 mg, 0.5 mmol; 1,4-dioxane (2 mL)) as colorless needles, mp 199–200 °C. ^{1}H NMR (400 MHz, DMSO-d_6): δ 3.16 (s, 3H), 3.21 (s, 3H), 5.08 (s, 1H), 5.47 (s, 2H), 6.56 (s, 1H), 6.57 (s, 1H), 7.09 (m, 1H), 7.14–7.34 (m, 4H). ^{13}C NMR (101 MHz, DMSO-d_6): δ 27.1, 29.8, 42.5, 87.4, 104.0 (t, *J* 25.8 Hz), 111.6 (dd, *J* 21.1, 3.6 Hz), 119.1 (dd, *J* 15.1, 3.6 Hz), 120.8, 130.9 (dd, *J* 10.0, 5.4 Hz), 144.0, 152.3, 152.8, 157.3, 160.0 (dd, *J* 249.1, 12.4 Hz), 162.0 (dd, *J* 246.7, 11.7 Hz), 162.4. ^{19}F NMR (377 MHz, DMSO-d_6): δ -112.57 (d, *J* 7.6 Hz), -110.12 (d, *J* 7.6 Hz). IR (ATR, KBr, cm^{-1}): *v* 3409, 3383, 3318, 3202, 1697, 1686, 1647, 1627, 1588, 1564, 1522, 1508, 1475, 1443, 1427, 1386, 1334, 1309, 1278, 1272, 1254, 1247, 1198, 1146, 1142, 1106, 1060, 1049. HRMS (ESI-TOF) *m/z*: [M+H]$^+$ Calcd for $C_{16}H_{17}F_2N_8O_2^+$ 391.1437; Found: 391.1437.

(Z)-5-Amino-1-cyclohexyl-N'-(1,3-dimethyl-2,6-dioxo-1,2,3,6-tetrahydropyrimidin-4-yl)-1H-1,2,3-triazole-4-carboximidamide (**3c**). Compound **3c** was obtained in 66% yield (299 mg) according to the general procedure A (DBU: 76 mg, 75 µL, 0.5 mmol; amidine **1g**: 87 mg, 0.5 mmol; azide **2a**: 91 mg, 0.5 mmol; 1,4-dioxane (2 mL)) as a colorless powder,

mp 340–341 °C. ^{1}H NMR (400 MHz, DMSO-d_6): δ 2.41 (s, 3H), 3.17 (s, 3H), 3.22 (s, 3H), 5.11 (s, 1H), 6.44 (s, 1H), 6.45 (s, 1H), 7.30 (br. s, 2H), 7.42 (d, J 8.3 Hz, 2H), 7.48 (d, J 8.3 Hz, 2H). ^{13}C NMR (101 MHz, DMSO-d_6): δ 20.6, 27.1, 29.8, 87.6, 120.8, 124.2, 130.1, 132.2, 138.9, 143.7, 152.3, 152.9, 157.2, 162.5. IR (ATR, KBr, cm^{-1}): ν 3486, 3399, 3315, 3190, 1692, 1631, 1590, 1567, 1549, 1519, 1501, 1465, 1436, 1404, 1384, 1319, 1305, 1292, 1270, 1213, 1194, 1116, 1089, 1054, 1039. HRMS (ESI-TOF) *m/z*: [M+H]$^+$ Calcd for $C_{16}H_{19}N_8O_2^+$ 355.1625; Found: 355.1628.

(Z)-5-Amino-1-(2,4-difluorobenzyl)-N'-(1,3-dimethyl-2,6-dioxo-1,2,3,6-tetrahydropyrimidin-4-yl)-1H-1,2,3-triazole-4-carboximidamide (**3d**). Compound **3d** was obtained in 79% yield (121 mg) according to the general procedure A (DBU: 76 mg, 75 µL, 0.5 mmol; amidine **1c**: 63 mg, 0.5 mmol; azide **2a**: 91 mg, 0.5 mmol; 1,4-dioxane (2 mL)) as a colorless powder, mp 200–201 °C. ^{1}H NMR (400 MHz, DMSO-d_6): δ 0.89 (t, J 7.4 Hz, 3H), 1.74–1.79 (m, 1H), 3.16 (s, 3H), 3.21 (s, 3H), 4.12 (t, J 7.2 Hz, 2H), 5.07 (s, 1H), 6.40 (s, 2H), 7.15 (br. s, 2H). ^{13}C NMR (101 MHz, DMSO-d_6): δ 10.7, 21.6, 27.1, 29.8, 46.8, 87.3, 120.8, 143.6, 152.3, 153.0, 157.3, 162.5. IR (ATR, KBr, cm^{-1}): ν 3405, 3315, 3198, 2963, 2934, 2877, 1701, 1688, 1647, 1623, 1591, 1566, 1519, 1476, 1442, 1425, 1385, 1350, 1310, 1296, 1284, 1268, 1246, 1196, 1037. HRMS (ESI-TOF) *m/z*: [M+H]$^+$ Calcd for $C_{12}H_{19}N_8O_2^+$ 307.1625; Found: 307.1623.

(Z)-5-Amino-1-(2,4-difluorobenzyl)-N'-(1,3-dimethyl-2,6-dioxo-1,2,3,6-tetrahydropyrimidin-4-yl)-1H-1,2,3-triazole-4-carboximidamide (**3e**). Compound **3e** was obtained in 77% yield (117 mg) according to the general procedure A (DBU: 76 mg, 75 µL, 0.5 mmol; amidine **1d**: 62 mg, 0.5 mmol; azide **2a**: 91 mg, 0.5 mmol; 1,4-dioxane (2 mL)) as a colorless powder, mp 207–208 °C. ^{1}H NMR (400 MHz, DMSO-d_6): δ 3.16 (s, 3H), 3.21 (s, 3H), 4.84 (d, J 5.4 Hz, 2H), 5.07 (s, 1H), 5.10 (d, J 15.0 Hz, 1H), 5.23 (d, J 10.3 Hz, 1H), 5.92–6.01 (m, 1H), 6.41 (s, 2H), 7.18 (br. s, 1H). ^{13}C NMR (101 MHz, DMSO-d_6): δ 27.1, 29.8, 47.5, 87.3, 117.7, 120.8, 131.8, 143.7, 152.3, 153.0, 157.3, 162.4. IR (ATR, KBr, cm^{-1}): ν 3473, 3393, 3298, 3190, 1692, 1636, 1572, 1553, 1523, 1475, 1432, 1385, 1315, 1275, 1251, 1200, 1163, 1101, 1059. HRMS (ESI-TOF) *m/z*: [M+H]$^+$ Calcd for $C_{12}H_{17}N_8O_2^+$ 305.1469; Found: 305.1473.

(Z)-5-Amino-1-(2,4-difluorobenzyl)-N'-(1,3-dimethyl-2,6-dioxo-1,2,3,6-tetrahydropyrimidin-4-yl)-1H-1,2,3-triazole-4-carboximidamide (**3f**). Compound **3f** was obtained in 78% yield (117 mg) according to the general procedure A (DBU: 76 mg, 75 µL, 0.5 mmol; amidine **1e**: 61 mg, 0.5 mmol; azide **2a**: 91 mg, 0.5 mmol; 1,4-dioxane (2 mL)) as a colorless powder, mp 218–219 °C. ^{1}H NMR (400 MHz, DMSO-d_6): δ 3.16 (s, 3H), 3.21 (s, 3H), 3.44 (t, J 2.5 Hz, 1H), 5.07 (s, 1H), 5.13 (d, J 2.5 Hz, 2H), 6.55 (s, 1H), 6.56 (s, 1H), 7.19 (br. s, 1H). ^{13}C NMR (101 MHz, DMSO-d_6): δ 27.1, 29.8, 35.3, 76.1, 77.1, 87.4, 120.8, 143.6, 152.3, 152.8, 157.3, 162.5. IR (ATR, KBr, cm^{-1}): ν 3439, 3388, 3315, 3285, 3225, 2947, 2132, 1695, 1638, 1610, 1578, 1562, 1511, 1450, 1432, 1402, 1384, 1310, 1280, 1246, 1217, 1202, 1106, 1049, 1021. HRMS (ESI-TOF) *m/z*: [M+H]$^+$ Calcd for $C_{12}H_{15}N_8O_2^+$ 303.1312; Found: 303.1311.

(Z)-5-Amino-1-cyclohexyl-N'-(1,3-dimethyl-2,6-dioxo-1,2,3,6-tetrahydropyrimidin-4-yl)-1H-1,2,3-triazole-4-carboximidamide (**3g**). Compound **3g** was obtained in 66% yield (299 mg) according to the general procedure A (DBU: 76 mg, 75 µL, 0.5 mmol; amidine **1f**: 83 mg, 0.5 mmol; azide **2a**: 91 mg, 0.5 mmol; 1,4-dioxane (2 mL)) as colorless needles, mp 207–208 °C. ^{1}H NMR (400 MHz, DMSO-d_6): δ 1.22–1.30 (m, 1H), 1.34–1.47 (m, 2H), 1.67–70 (m, 1H), 1.75–1.90 (m, 4H), 1.92–2.00 (m, 2H), 3.16 (s, 3H), 3.21 (s, 3H), 4.19–

27 (m, 1H), 5.07 (s, 1H), 6.38 (s, 1H), 6.39 (s, 1H), 7.14 (s, 2H). ^{13}C NMR (101 MHz, DMSO-d_6): δ 24.6, 24.8, 27.1, 29.7, 31.5, 54.7, 87.3, 120.8, 142.8, 152.3, 153.0, 157.3, 162.4. IR (ATR, KBr, cm^{-1}): v 3494, 3366, 3284, 3136, 3102, 2931, 2853, 1687, 1631, 1588, 1568, 1533, 1505, 1461, 1452, 1437, 1388, 1371, 1353, 1336, 1308, 1242, 1219, 1199, 1169, 1154, 1087, 1059, 1049, 1012. HRMS (ESI-TOF) *m/z*: $[M+H]^+$ Calcd for $C_{15}H_{23}N_8O_2^+$ 347.1938; Found: 347.1939.

(Z)-5-Amino-1-cyclohexyl-N'-(1,3-dimethyl-2,6-dioxo-1,2,3,6-tetrahydropyrimidin-4-yl)-1H-1,2,3-triazole-4-carboximidamide (**3h**). Compound **3h** was obtained in 54% yield (95 mg) according to the general procedure A (DBU: 76 mg, 75 µL, 0.5 mmol; amidine **1h**: 86 mg, 0.5 mmol; azide **2a**: 91 mg, 0.5 mmol; 1,4-dioxane (2 mL)) as a colorless powder, mp 222–223 °C. ^{1}H NMR (400 MHz, DMSO-d_6): δ 3.16 (s, 3H), 3.21 (s, 3H), 3.32 (s, 6H), 4.31 (d, J 5.5 Hz, 2H), 4.79 (t, J 5.5 Hz, 1H), 5.07 (s, 1H), 6.40 (s, 2H), 7.17 (br. s, 2H). ^{13}C NMR (101 MHz, DMSO-d_6): δ 27.1, 29.8, 47.0, 53.8, 87.4, 101.1, 120.7, 144.3, 152.3, 152.9, 157.3, 162.4. IR (ATR, KBr, cm^{-1}): v 3402, 3310, 3200, 3108, 2994, 2955, 2928, 2880 2834, 1702, 1644, 1623, 1596, 1587, 1565, 1521, 1473, 1440, 1384,1330, 1312, 1275, 1253, 1192, 1146, 1113, 1067, 1047, 1040, 1015. HRMS (ESI-TOF) *m/z*: $[M+H]^+$ Calcd for $C_{13}H_{21}N_8O_4^+$ 353.1680; Found: 353.1684.

(Z)-1-(2-(1H-Indol-3-yl)ethyl)-5-amino-N'-(1,3-dimethyl-2,6-dioxo-1,2,3,6-tetrahydropyrimidin-4-yl)-1H-1,2,3-triazole-4-carboximidamide (**3i**). Compound **3i** was obtained in 74% yield (151 mg) according to the general procedure A (DBU: 76 mg, 75 µL, 0.5 mmol; amidine **1i**: 113 mg, 0.5 mmol; azide **2a**: 91 mg, 0.5 mmol; 1,4-dioxane (2 mL)) as a colorless powder, mp 242–243 °C. ^{1}H NMR (400 MHz, DMSO-d_6): δ 3.17 (s, 3H), 3.21 (s, 3H), 3.22 (t, J 7.4 Hz, 2H), 4.46 (t, J 7.4 Hz, 2H), 5.08 (s, 1H), 6.42 (s, 2H), 6.99 (t, J 7.8 Hz, 1H), 7.08 (t, J 7.1 Hz, 1H), 7.17 (s, 2H), 7.35 (d, J 8.1 Hz, 1H), 7.55 (d, J 7.9 Hz, 1H), 10.82 (s, 1H). ^{13}C NMR (101 MHz, DMSO-d_6): δ 24.2, 27.1, 29.8, 46.0, 87.4, 110.2, 111.3, 118.1, 118.3, 121.0, 123.0, 127.0, 136.1, 143.7, 152.3, 153.0, 157.3, 162.5. IR (ATR, KBr, cm^{-1}): v 3449, 3377, 3343, 3206, 1687, 1640, 1554, 1524, 1455, 1428, 1403, 1385, 1366, 1347, 1339, 1315, 1272, 1250, 1232, 1213, 1200, 1162, 1153, 1102, 1049,1012. HRMS (ESI-TOF) *m/z*: $[M+H]^+$ Calcd for $C_{19}H_{22}N_9O_2^+$ 408.1891; Found: 408.1889.

(Z)-5-Amino-N'-(1,3-dimethyl-2,6-dioxo-1,2,3,6-tetrahydropyrimidin-4-yl)-1-(2-(5-methoxy-1H-indol-3-yl)ethyl)-1H-1,2,3-triazole-4-carboximidamide (**3j**). Compound **3j** was obtained in 76% yield (166 mg) according to the general procedure A (DBU: 76 mg, 75 µL, 0.5 mmol; amidine **1j**: 128 mg, 0.5 mmol; azide **2a**: 90 mg, 0.5 mmol; 1,4-dioxane (2 mL)) as a colorless powder, mp 200–201 °C. ^{1}H NMR (400 MHz, DMSO-d_6): δ 3.17 (s, 3H), 3.17 (t, J 7.4 Hz, 2H), 3.20 (s, 3H), 3.75 (s, 3H), 4.44 (t, J 7.4 Hz, 2H), 5.07 (s, 1H), 6.38 (s, 1H), 6.39 (s, 1H), 6.71 (dd, J 8.8, 2.4 Hz, 1H), 6.93 (d, J 2.4 Hz, 1H), 7.13 (s, 1H), 7.16 (br. s, 2H), 7.23 (d, J 8.8 Hz, 1H), 10.65 (s, 1H). ^{13}C NMR (101 MHz, DMSO-d_6): δ 24.2, 27.1, 29.7, 46.2, 55.3, 87.3, 100.0, 110.1, 111.1, 111.9, 121.0, 123.7, 127.4, 131.2, 143.7, 152.3, 153.0, 157.3, 162.5. IR (ATR, KBr, cm^{-1}): v 3402, 3311, 3199, 2995, 2951, 1700, 1687, 1645, 1626, 1586, 1564, 1520, 1485, 1454, 1441, 1428, 1385, 1309, 1274, 1251, 1212, 1200, 1175, 1094,1051, 1027. HRMS (ESI-TOF) *m/z*: $[M+H]^+$ Calcd for $C_{20}H_{24}N_9O_3^+$ 438.1996; Found: 438.1997.

(Z)-5-Amino-1-benzyl-N'-(1,3-dicyclohexyl-2,6-dioxo-1,2,3,6-tetrahydropyrimidin-4-yl)-1H-1,2,3-triazole-4-carboximidamide (**3k**). Compound **3k** was obtained in 94% yield (230 mg) according to the general procedure A (DBU: 76 mg, 75 µL, 0.5 mmol; amidine **1a**: 86

mg, 0.5 mmol; azide **2b**: 158 mg, 0.5 mmol; 1,4-dioxane (2 mL)) as a colorless powder, mp 236–237 °C. ^{1}H NMR (400 MHz, DMSO-*d*$_6$): *δ* 0.98–1.17 (m, 4H), 1.22–1.32 (m, 2H), 1.47–1.56 (m, 3H), 1.50–1.65 (m, 3H), 1.70–1.79 (m, 4H), 2.24–2.41 (m, 4H), 4.38–4.44 (m, 1H), 4.63–4.70 (m, 1H), 4.97 (s, 1H), 5.46 (s, 2H), 6.50 (s, 2H), 7.24–7.38 (m, 7H). ^{13}C NMR (101 MHz, DMSO-*d*$_6$): *δ* 24.9, 25.1, 26.0, 28.3, 29.0, 48.5, 51.9, 55.7, 88.0, 121.0, 127.3, 127.6, 128.5, 135.6, 143.8, 151.4, 152.8, 157.0, 162.7. IR (ATR, KBr, cm^{-1}): *v* 3401, 3291, 3242, 3183, 2930, 2853, 1689, 1644, 1614, 1593, 1563, 1497, 1451, 1439, 1426, 1406, 1383, 1369, 1347, 1331, 1309, 1286, 1254, 1225, 1193, 1162, 1105, 1092, 1048, 1030. HRMS (ESI-TOF) *m/z*: [M+H]$^+$ Calcd for $C_{26}H_{35}N_8O_2^+$ 491.2878; Found: 491.2876.

(Z)-5-Amino-1-benzyl-N'-(thiazol-2-yl)-1H-1,2,3-triazole-4-carboximidamide (**3l**). Compound **3l** was obtained in 88% yield (117 mg) according to the general procedure B (NaOH: 20 mg, 0.5 mmol; amidine **1a**: 77 mg, 0.5 mmol; azide **2c**: 56 mg, 0.5 mmol; ethanol (2 mL)) as light yellow needles, mp 199–200 °C. ^{1}H NMR (400 MHz, DMSO-*d*$_6$): *δ* 5.48 (s, 2H), 6.68 (s, 1H), 6.69 (s, 1H), 7.10 (d, *J* 3.9 Hz, 1H), 7.24 (d, *J* 6.9 Hz, 2H), 7.28–7.38 (m, 3H), 7.43 (d, *J* 3.9 Hz, 1H), 8.42 (br. s, 1H), 9.15 (br. s, 1H). ^{13}C NMR (101 MHz, DMSO-*d*$_6$): *δ* 48.5, 112.6, 121.2, 127.2, 127.6, 128.5, 135.7, 138.8, 143.6, 153.5, 174.5. IR (ATR, KBr, cm^{-1}): *v* 3400, 3359, 3255, 1625, 1602, 1563, 1554, 1508, 1495, 1483, 1455, 1436, 1425, 1402, 1385, 1356, 1332, 1319, 1303, 1283, 1257, 1215, 1151, 1095, 1067, 1053, 1035, 1011. HRMS (ESI-TOF) *m/z*: [M+H]$^+$ Calcd for $C_{13}H_{14}N_7S^+$ 300.1026; Found: 300.1031.

(Z)-5-Amino-1-benzyl-N'-(4H-1,2,4-triazol-3-yl)-1H-1,2,3-triazole-4-carboximidamide (**3m**). Compound **3m** was obtained in 51% yield (72 mg) according to the general procedure B (NaOH: 20 mg, 0.5 mmol; amidine **1a**: 87 mg, 0.5 mmol; azide **2f**: 55 mg, 0.5 mmol; ethanol (2 mL)) as a colorless powder, mp 259–260 °C. ^{1}H NMR (400 MHz, DMSO-*d*$_6$): *δ* 5.47 (s, 2H), 6.85 (s, 2H), 7.25 (d, *J* Hz, 2H), 7.25–7.30 (m, 1H), 7.32–7.39 (m, 2H), 7.75 (s, 1H), 7.96 (s, 1H), 8.81 (s, 1H), 12.93 (s, 1H). ^{13}C NMR (101 MHz, DMSO-*d*$_6$): *δ* 48.5, 121.4, 127.2, 127.6, 128.6, 135.7, 143.9, 148.8, 154.2, 158.7. IR (ATR, KBr, cm^{-1}): *v* 3438, 3359, 3277, 3223, 3168, 1630, 1612, 1566, 1537, 1515, 1496, 1474, 1455, 1448, 1403, 1384, 1374, 1365, 1332, 1306, 1292, 1274, 1263, 1241, 1206, 1196, 1157, 1103, 1082, 1072, 1028, 1013. HRMS (ESI-TOF) *m/z*: [M+H]$^+$ Calcd for $C_{12}H_{14}N_9^+$ 284.1367; Found: 284.1369.

(Z)-5-Amino-1-cyclohexyl-N'-(4H-1,2,4-triazol-3-yl)-1H-1,2,3-triazole-4-carboximidamide (**3n**). Compound **3n** was obtained in 46% yield (63 mg) according to the general procedure B (NaOH: 20 mg, 0.5 mmol; amidine **1f**: 83 mg, 0.5 mmol; azide **2f**: 55 mg, 0.5 mmol; ethanol (2 mL)) as a colorless powder, mp 255–256 °C. ^{1}H NMR (400 MHz, DMSO-*d*$_6$): *δ* 1.25–1.46 (m, 3H), 1.68–1.99 (m, 7H), 4.18–4.23 (m, 1H), 6.67 (s, 1H), 6.69 (s, 1H), 7.74 (s, 1H), 7.88 (s, 1H), 8.80 (s, 1H), 12.95 (s, 1H). ^{13}C NMR (101 MHz, DMSO-*d*$_6$): *δ* 24.7, 24.9, 31.6, 54.8, 121.3, 142.8, 148.7, 154.4, 158.5. IR (ATR, KBr, cm^{-1}): *v* 3455, 3367, 3278, 3223, 2940, 2920, 2908, 2859, 1628, 1608, 1564, 1542, 1529, 1512, 1472, 1450, 1384, 1292, 1268, 1248, 1199, 1153, 1101, 1085, 1075, 1015. HRMS (ESI-TOF) *m/z*: [M+H]$^+$ Calcd for $C_{11}H_{18}N_9^+$ 276.1680; Found: 276.1683.

(Z)-5-Amino-1-benzyl-N'-(1-methyl-4-nitro-1H-imidazol-5-yl)-1H-1,2,3-triazole-4-carboximidamide (**3o**). Compound **3o** was obtained in 77% yield (132 mg) according to the general procedure A (DBU: 76 mg, 75 µL, 0.5 mmol; amidine **1a**: 86 mg, 0.5 mmol; azide **2d**: 176 mg, 0.524 mmol; 1,4-dioxane (2 mL)) as a yellow powder, mp 269–270 °C.

^{1}H NMR (600 MHz, DMSO-d_6): δ 3.40 (s, 3H), 5.47 (s, 2H), 6.60 (s, 2H), 7.21 (br. s, 2H), 7.27–7.40 (m, 5H), 7.55 (s, 1H). ^{13}C NMR (151 MHz, DMSO-d_6): δ 30.5, 48.5, 121.5, 127.5, 127.7, 128.6, 132.3, 134.4, 135.8, 140.8, 144.0, 153.9. IR (ATR, KBr, cm^{-1}): *v* 3398, 3330, 3299, 3151, 3132, 1648, 1628, 1599, 1564, 1555, 1518, 1498, 1554, 1435, 1426, 1369, 1357, 1336, 1329, 1306, 1283, 1270, 1251, 1237, 1200, 1106, 1074, 1061, 1030, 1012. HRMS (ESI-TOF) *m/z*: [M+H]$^+$ Calcd for $C_{14}H_{16}N_9O_2^+$ 342.1421; Found: 342.1421.

(Z)-5-Amino-1-(2,4-difluorobenzyl)-N'-(1-methyl-4-nitro-1H-imidazol-5-yl)-1H-1,2,3-triazole-4-carboximidamide (**3p**). Compound **3p** was obtained in 63% yield (118 mg) according to the general procedure A (DBU: 76 mg, 75 μL, 0.5 mmol; amidine **1b**: 104 mg, 0.5 mmol; azide **2d**: 88 mg, 0.524 mmol; 1,4-dioxane (2 mL)) as a yellow powder, mp 254–255 °C. ^{1}H NMR (400 MHz, DMSO-d_6): δ 3.42 (s, 3H), 5.48 (s, 2H), 6.60 (s, 2H), 7.09–7.32 (m, 5H), 7.54 (s, 1H). ^{13}C NMR (101 MHz, DMSO-d_6): δ 30.4, 42.5 (d, *J* 3.4 Hz), 104.0 (t, *J* 25.8 Hz), 111.6 (dd, *J* 21.4, 3.7 Hz), 119.1 (dd, *J* 15.1, 3.8 Hz), 121.4, 130.9 (dd, *J* 10.1, 5.3 Hz), 132.7, 134.4, 140.6, 144.1, 153.8, 160.0 (dd, *J* 249.2, 12.4 Hz), 162.0 (dd, *J* 247.0, 12.2 Hz). ^{19}F NMR (377 MHz, DMSO-d_6): δ -112.52 (d, *J* 7.7 Hz), -110.11 (d, *J* 7.7 Hz). IR (ATR, KBr, cm^{-1}): *v* 3492, 3403, 3328, 3294, 3162, 3132, 1652, 1624, 1600, 1566, 1553, 1519, 1508, 1455, 1431, 1372, 1361, 1335, 1308, 1274, 1252, 1237, 1201, 1174, 1138, 1109, 1090, 1059, 1030, 1013. HRMS (ESI-TOF) *m/z*: [M+H]$^+$ Calcd for $C_{14}H_{14}F_2N_9O_2^+$ 378.1233; Found: 378.1234.

(Z)-5-Amino-1-cyclohexyl-N'-(1-methyl-4-nitro-1H-imidazol-5-yl)-1H-1,2,3-triazole-4-carboximidamide (**3q**). Compound **3q** was obtained in 52% yield (86 mg) according to the general procedure A (DBU: 76 mg, 75 μL, 0.5 mmol; amidine **1f**: 82 mg, 0.5 mmol; azide **2d**: 88 mg, 0.524 mmol; 1,4-dioxane (2 mL)) as a yellow powder, mp 261–262 °C. ^{1}H NMR (600 MHz, DMSO-d_6): δ 1.24–1.31 (m, 1H), 1.37–1.45 (m, 2H), 1.67–1.70 (m, 1H), 1.78–1.86 (m, 4H), 1.96–1.98 (m, 2H), 3.41 (s, 3H), 4.20–4.26 (m, 1H), 6.47 (s, 2H), 7.16 (br. s, 2H), 7.55 (s, 1H). ^{13}C NMR (151 MHz, DMSO-d_6): δ 24.7, 24.9, 30.4, 31.7, 54.7, 121.4, 132.8, 134.4, 140.9, 143.1, 154.1. IR (ATR, KBr, cm^{-1}): *v* 3419, 3315, 3244, 3177, 3120, 2936, 2858, 1642, 1593, 1561, 1523, 1510, 1465, 1452, 1424, 1381, 1362, 1331, 1301, 1282, 1238, 1207, 1156, 1136, 1086, 1057, 1028. HRMS (ESI-TOF) *m/z*: [M+H]$^+$ Calcd for $C_{13}H_{20}N_9O_2^+$ 334.1734; Found: 334.1730.

(Z)-5-Amino-1-benzyl-N'-(5-(tert-butyl)isoxazol-3-yl)-1H-1,2,3-triazole-4-carboximidamide (**3r**). Compound **3r** was obtained in 86% yield (146 mg) according to the general procedure B (NaOH: 20 mg, 0.5 mmol; amidine **1a**: 86 mg, 0.5 mmol; azide **2e**: 83 mg, 0.5 mmol; ethanol (2 mL)) as a colorless powder, mp 223–224 °C. ^{1}H NMR (400 MHz, DMSO-d_6): δ 1.30 (s, 9H), 5.47 (s, 2H), 6.06 (s, 1H), 6.75 (s, 1H), 6.76 (s, 1H), 7.24 (d, *J* 7.0 Hz, 2H), 7.28–7.38 (m, 3H), 7.75 (br. s, 2H). ^{13}C NMR (101 MHz, DMSO-d_6): δ 28.4, 32.1, 48.4, 97.9, 121.5, 127.2, 127.6, 128.5, 135.8, 143.7, 153.8, 167.4, 178.7. IR (ATR, KBr, cm^{-1}): *v* 3407, 3294, 2966, 2934, 2908, 2872, 1632, 1599, 1572, 1559, 1519, 1496, 1476, 1454, 1436, 1416, 1370, 1360, 1333, 1320, 1303, 1272, 1263, 1191, 1104, 1074, 1030, 1002. HRMS (ESI-TOF) *m/z*: [M+H]$^+$ Calcd for $C_{17}H_{22}N_7O^+$ 340.1880; Found: 340.1881.

(Z)-5-Amino-N'-(5-(tert-butyl)isoxazol-3-yl)-1-cyclohexyl-1H-1,2,3-triazole-4-carboximidamide (**3s**). Compound **3s** was obtained in 92% yield (152 mg) according to the general procedure B (NaOH: 20 mg, 0.5 mmol; amidine **1f**: 82 mg, 0.5 mmol; azide **2e**: 84 mg, 0.5 mmol; ethanol (2 mL)) as colorless needles, mp 231–232 °C. ^{1}H NMR

(400 MHz, DMSO-d_6): δ 1.30 (s, 9H), 1.24–1.43 (m, 3H), 1.68–1.98 (m, 7H), 4.18–4.25 (m, 1H), 6.05 (s, 1H), 6.59 (s, 1H), 6.61 (s, 1H), 7.71 (br. s, 2H). ^{13}C NMR (101 MHz, DMSO-d_6): δ 24.7, 24.8, 28.4, 31.6, 32.1, 54.8, 97.9, 121.5, 142.7, 154.1, 167.4, 178.7. IR (ATR, KBr, cm^{-1}): v 3394, 3292, 3270, 2970, 2959, 2940, 2921, 2858, 1630, 1601, 1558, 1501, 1477, 1466, 1442, 1413, 1384, 1364, 1353, 1312, 1279, 1254, 1247, 1206, 1196, 1151, 1101, 1046, 1030, 1009. HRMS (ESI-TOF) *m/z*: [M+H]$^+$ Calcd for $C_{16}H_{26}N_7O$ 332.2193$^+$; Found: 332.2190.

(Z)-5-Amino-1-benzyl-N'-(2-oxo-2H-chromen-4-yl)-1H-1,2,3-triazole-4-carboximidamide (**3t**). Compound **3t** was obtained in 96% yield (138 mg) according to the general procedure A (DBU: 76 mg, 75 µL, 0.5 mmol; amidine **1a**: 86 mg, 0.5 mmol; azide **2g**: 94 mg, 0.5 mmol; 1,4-dioxane (2 mL)) as a colorless powder, mp 229–230°C. ^{1}H NMR (400 MHz, DMSO-d_6): δ 5.50 (s, 2H), 5.77 (s, 1H), 6.57 (s, 1H), 6.58 (s, 1H), 7.03 (s, 2H), 7.26–7.41 (m, 7H), 7.58 (t, *J* 7.2 Hz, 1H), 7.68 (d, *J* 7.8 Hz, 1H). ^{13}C NMR (101 MHz, DMSO-d_6): δ 48.5, 100.5, 116.2, 118.4, 121.4, 123.5, 124.6, 127.4, 127.7, 128.6, 135.7, 143.8, 152.0, 153.7, 160.1, 161.7. IR (ATR, KBr, cm^{-1}): v 3421, 3382, 3327, 3282, 3208, 1707, 1683, 1640, 1600, 1558, 1528, 1496, 1482, 1455, 1448, 1400, 1384, 1363, 1354, 1326, 1310, 1285, 1272, 1257, 1245, 1216, 1180, 1153, 1136, 1108, 1096, 1068, 1030. HRMS (ESI-TOF) *m/z*: [M+H]$^+$ Calcd for $C_{19}H_{17}N_6O_2^+$ 361.1408; Found: 361.1407.

*(Z)-5-Amino-1-cyclohexyl-N'-(2-oxo-2H-chromen-4-yl)-1H-1,2,3-triazole-4-carboximidamide (***3u***).* Compound **3u** was obtained in 94% yield (166 mg) according to the general procedure A (DBU: 76 mg, 75 µL, 0.5 mmol; amidine **1f**: 82 mg, 0.5 mmol; azide **2g**: 94 mg, 0.5 mmol; 1,4-dioxane (2 mL)) as a colorless powder, mp 221–222°C. ^{1}H NMR (600 MHz, DMSO-d_6): δ 1.23–1.30 (m, 1H), 1.38–1.45 (m, 2H), 1.68–1.70 (m, 1H), 1.80–1.87 (m, 4H), 1.98–2.00 (m, 2H), 4.23–4.28 (m, 1H), 5.75 (s, 1H), 6.49 (s, 2H), 7.06 (br. s, 2H), 7.27–7.30 (m, 1H), 7.36–7.38 (m, 1H), 7.59 (ddd, J = 8.6, 7.3, 1.6 Hz, 1H), 7.68 (dd, *J* 7.9, 1.5 Hz, 1H). ^{13}C NMR (151 MHz, DMSO-d_6): δ 24.8, 25.0, 31.7, 54.8, 100.6, 116.3, 118.6, 121.3, 123.6, 124.7, 131.7, 142.9, 152.3, 153.8, 160.3, 161.8. IR (ATR, KBr, cm^{-1}): v 3457, 3404, 3362, 3311, 3205, 2929, 2855, 1702, 1682, 1639, 1596, 1553, 1540, 1508, 1482, 1447, 1385, 1365, 1328, 1301, 1269, 1242, 1214, 1183, 1164, 1152, 1137, 1106, 1031. HRMS (ESI-TOF) *m/z*: [M+H]$^+$ Calcd for $C_{18}H_{21}N_6O_2^+$ 353.1720; Found: 353.1716.
