# Supplementary material for: Cycloaddition reactions of heterocyclic azides with 2-cyanoacetamidines as a new route to C,N-diheteroarylcarbamidines
Source: Beilstein J Org Chem. 2024 Jan 5;20:17–24. doi: 10.3762/bjoc.20.3 (PMC10783006; doi:10.3762/bjoc.20.3)
Supplement: File 2 — Copies of NMR spectra of all new compounds. [file Beilstein_J_Org_Chem-20-17-s002.pdf]

BEILSTEIN JOURNAL OF ORGANIC CHEMISTRY

Supporting Information

for

Cycloaddition reactions of heterocyclic azides with 2-cyanoacetamidines as a new route to *C,N*-diheteroarylcarbamidines

Pavel S. Silaichev, Tetyana V. Beryozkina, Vsevolod V. Melekhin, Valeriy O. Filimonov, Andrey N. Maslivets, Vladimir G. Ilkin, Wim Dehaen and Vasiliy A. Bakulev

Beilstein J. Org. Chem. **2024,** *20,* 17–24. doi:10.3762/bjoc.20.3

Copies of NMR spectra of all new compounds

License and Terms: This is a supporting information file under the terms of the Creative Commons Attribution License (https://creativecommons.org/licenses/by/4.0). Please note that the reuse, redistribution and reproduction in particular requires that the author(s) and source are credited and that individual graphics may be subject to special legal provisions.
The license is subject to the *Beilstein Journal of Organic Chemistry* terms and conditions: (https://www.beilstein-journals.org/bjoc/terms)

Table of contents

NMR spectra of compounds **3a–u** S2

X-ray Single Crystal Data for **3g** S28

Fig. S1. ^{1}H NMR spectrum (DMSO-d_6) of **3a**

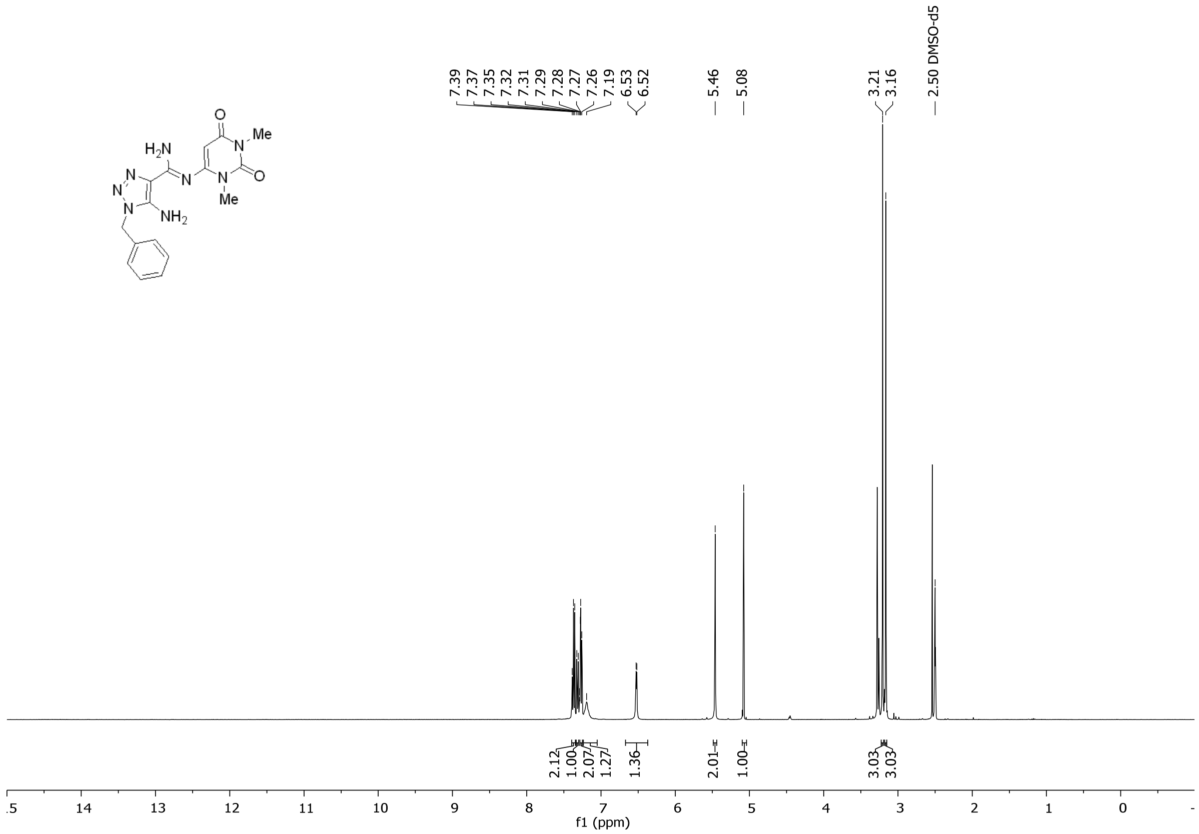

Fig. S2. ^{13}C NMR spectrum (DMSO-d_6) of **3a**

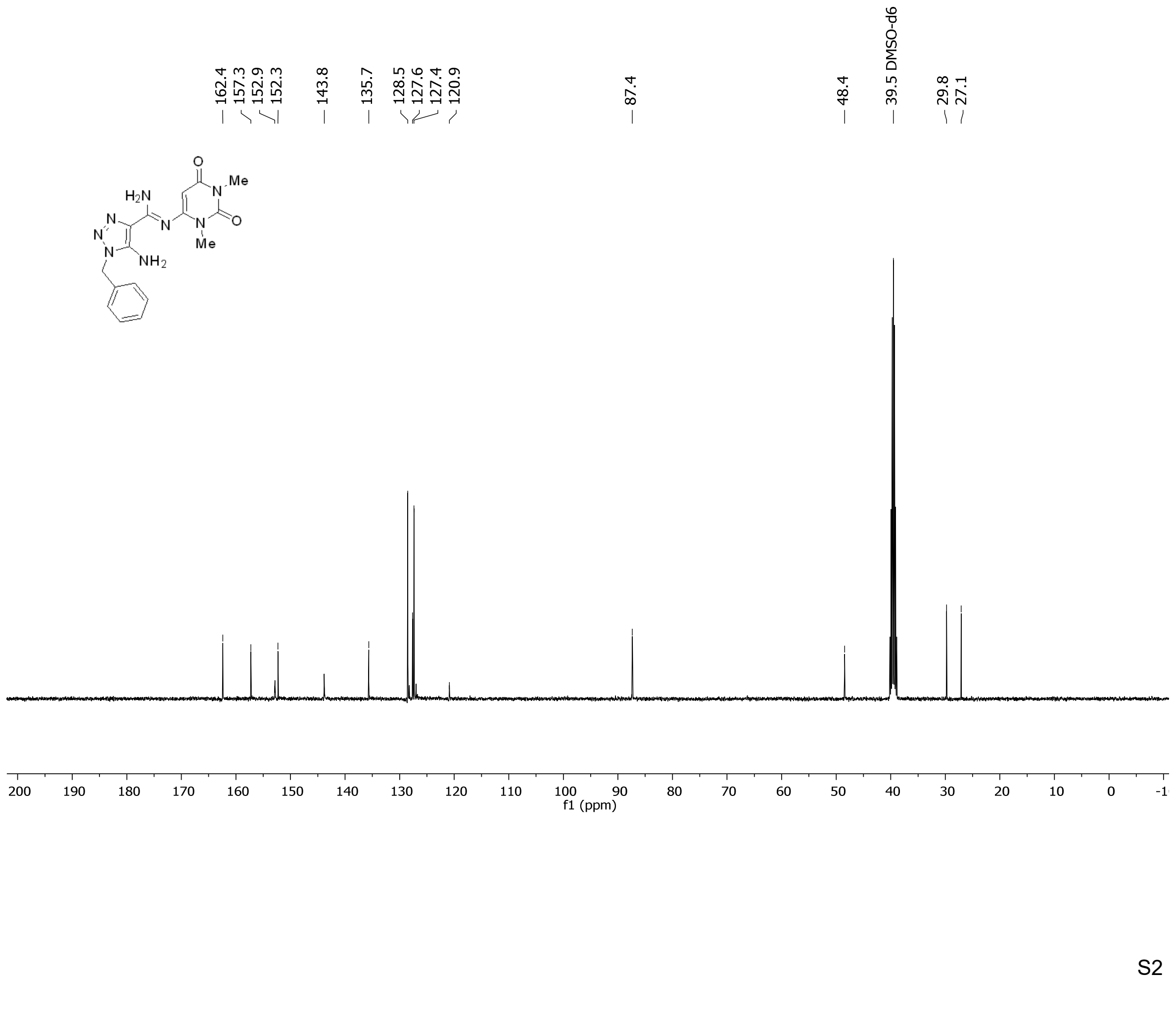

Fig. S3. ^{1}H NMR spectrum (DMSO-d_6) of **3b**

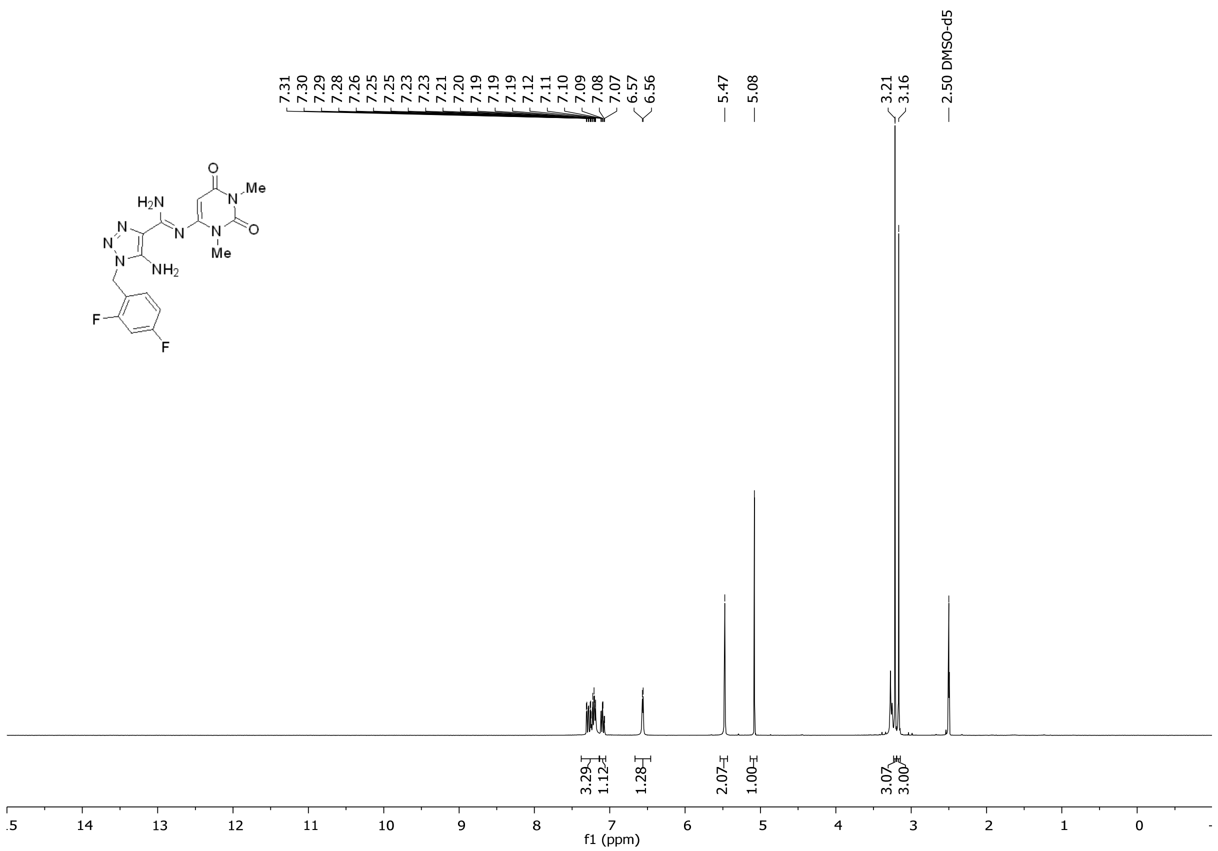

Fig. S4. ^{13}C NMR spectrum (DMSO-d_6) of **3b**

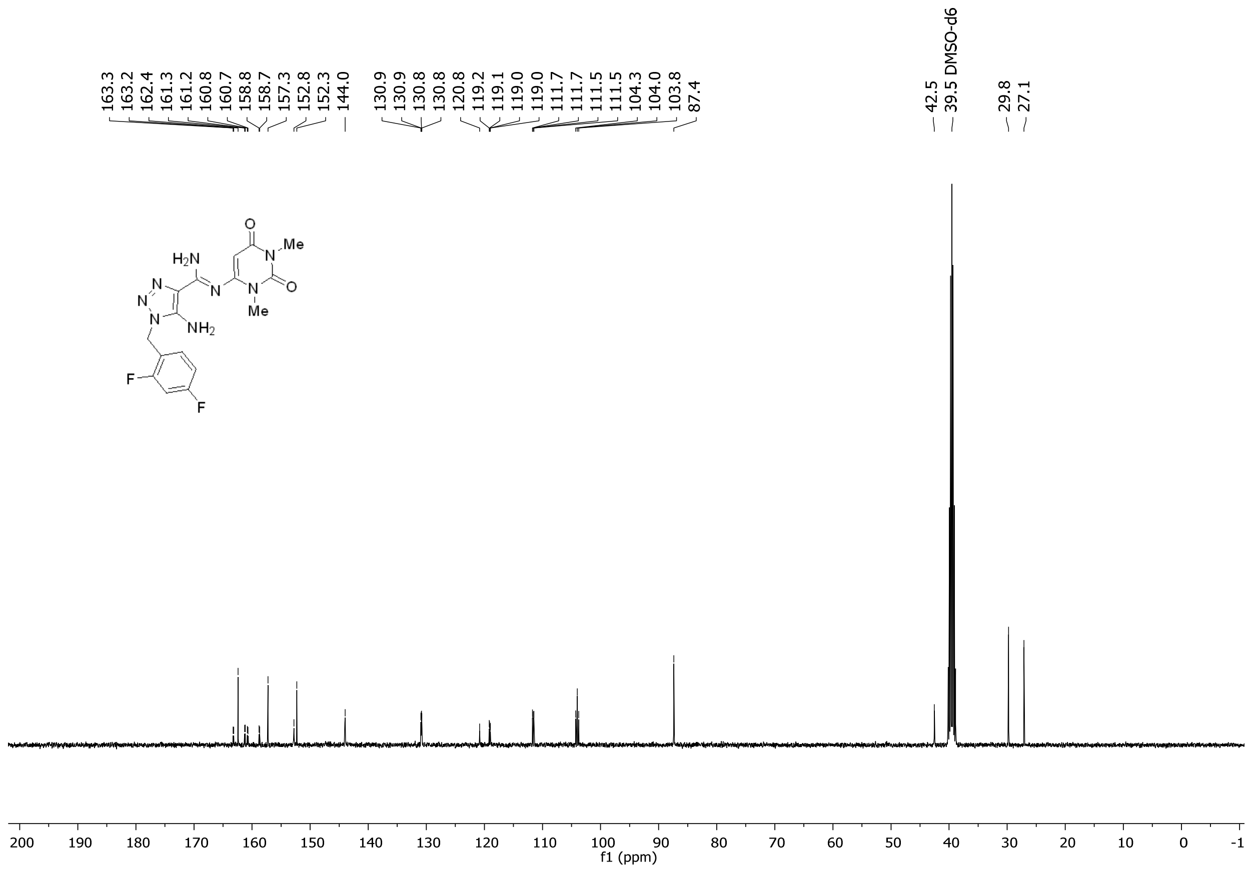

Fig. S5. ^{19}F NMR spectrum (DMSO-d_6) of **3b**

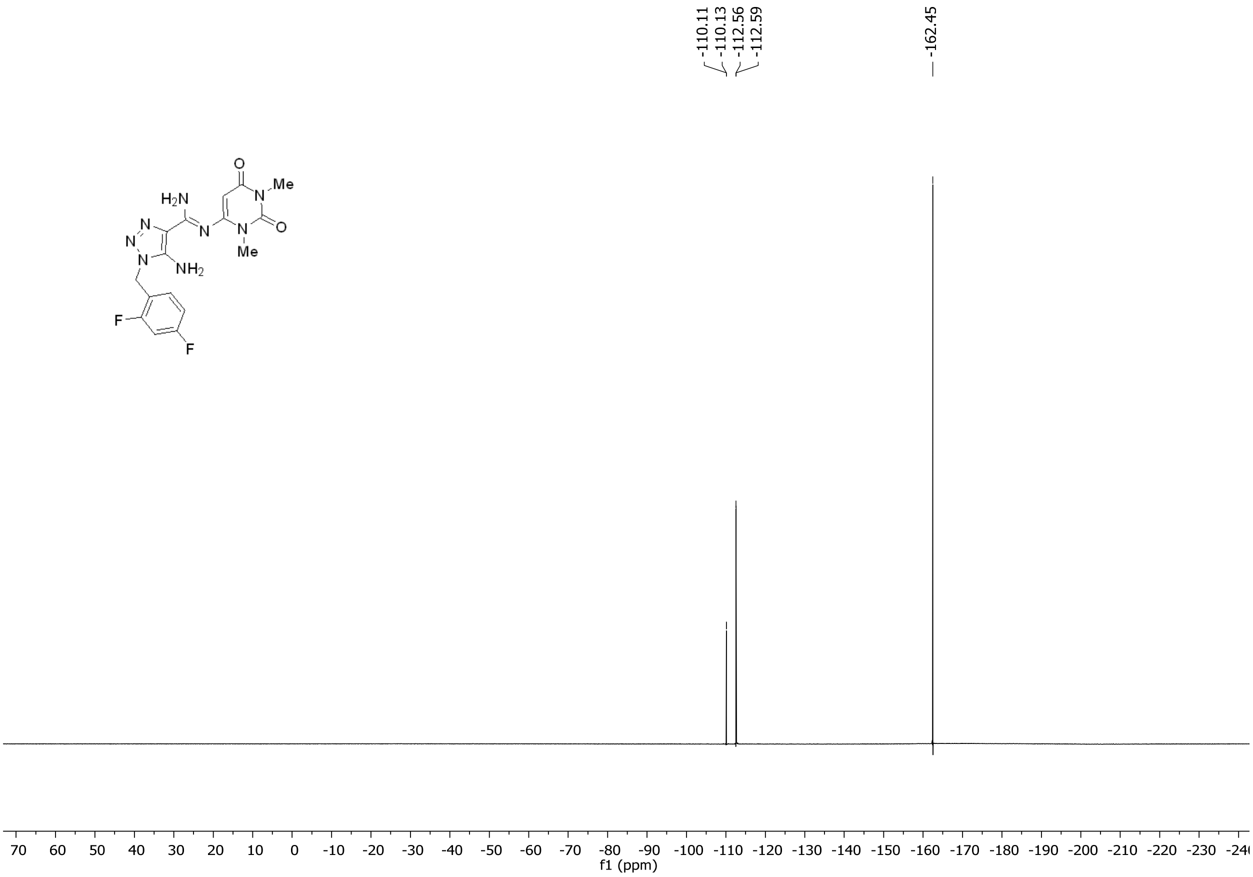

Fig. S6. ^{1}H NMR spectrum (DMSO-d_6) of **3c**

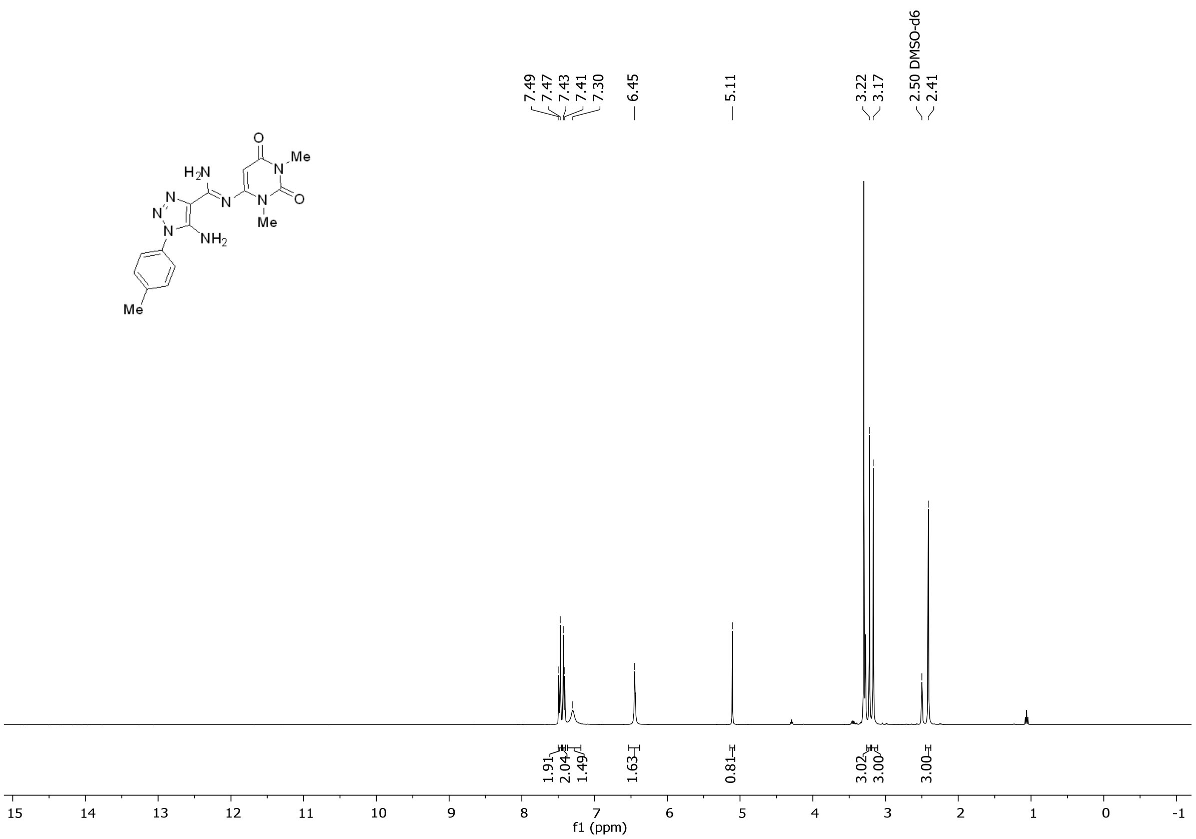

Fig. S7. ^{13}C NMR spectrum (DMSO-d_6) of **3c**

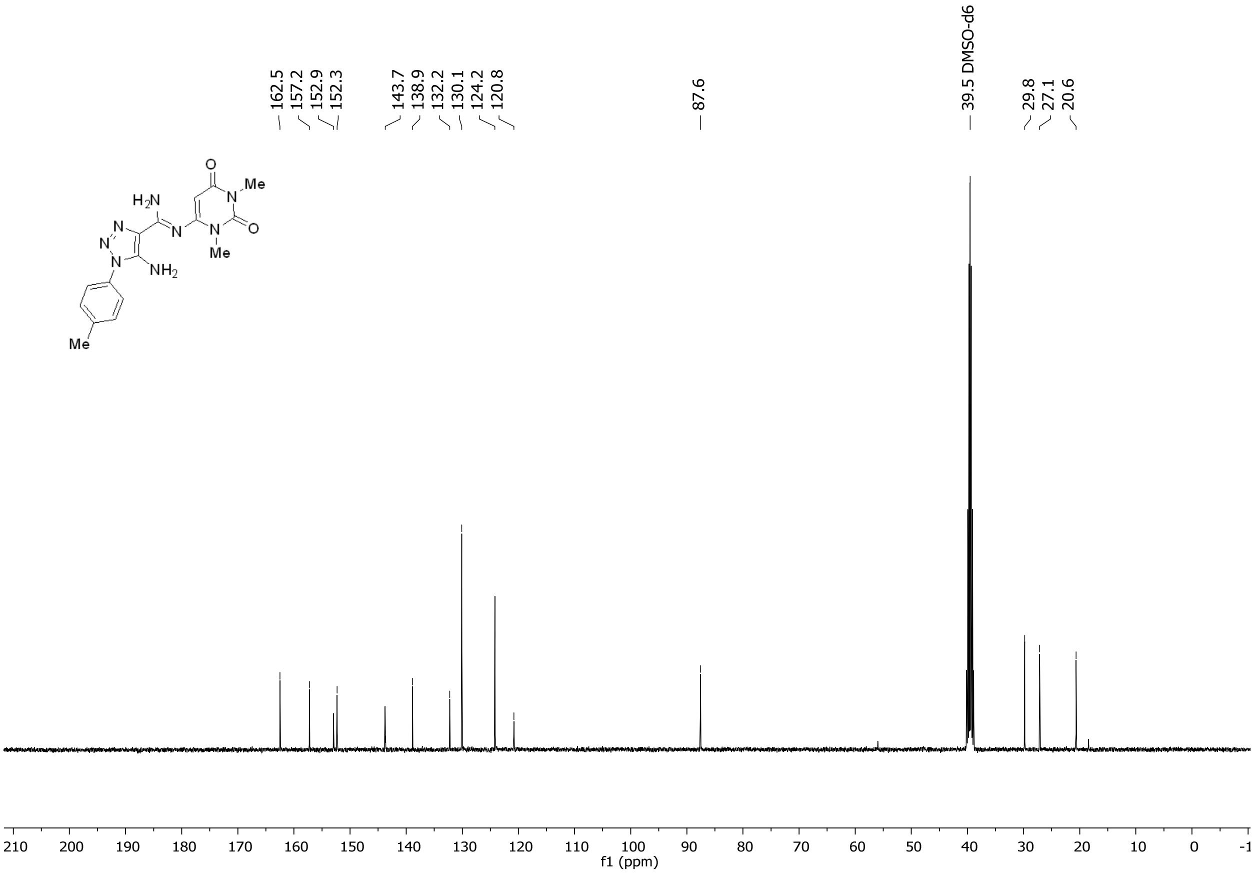

Fig. S8. ^{1}H NMR spectrum (DMSO-d_6) of **3d**

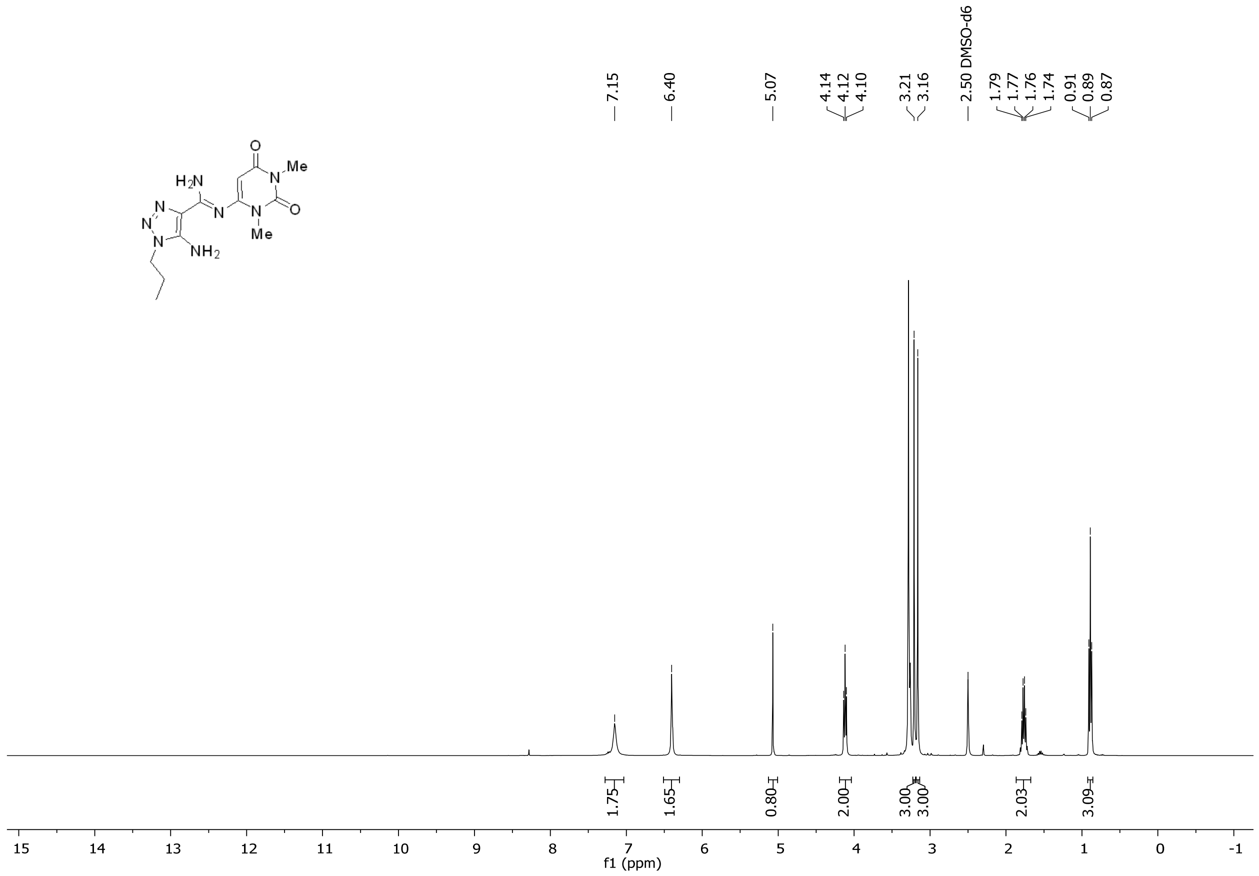

Fig. S9. ^{13}C NMR spectrum (DMSO-d_6) of **3d**

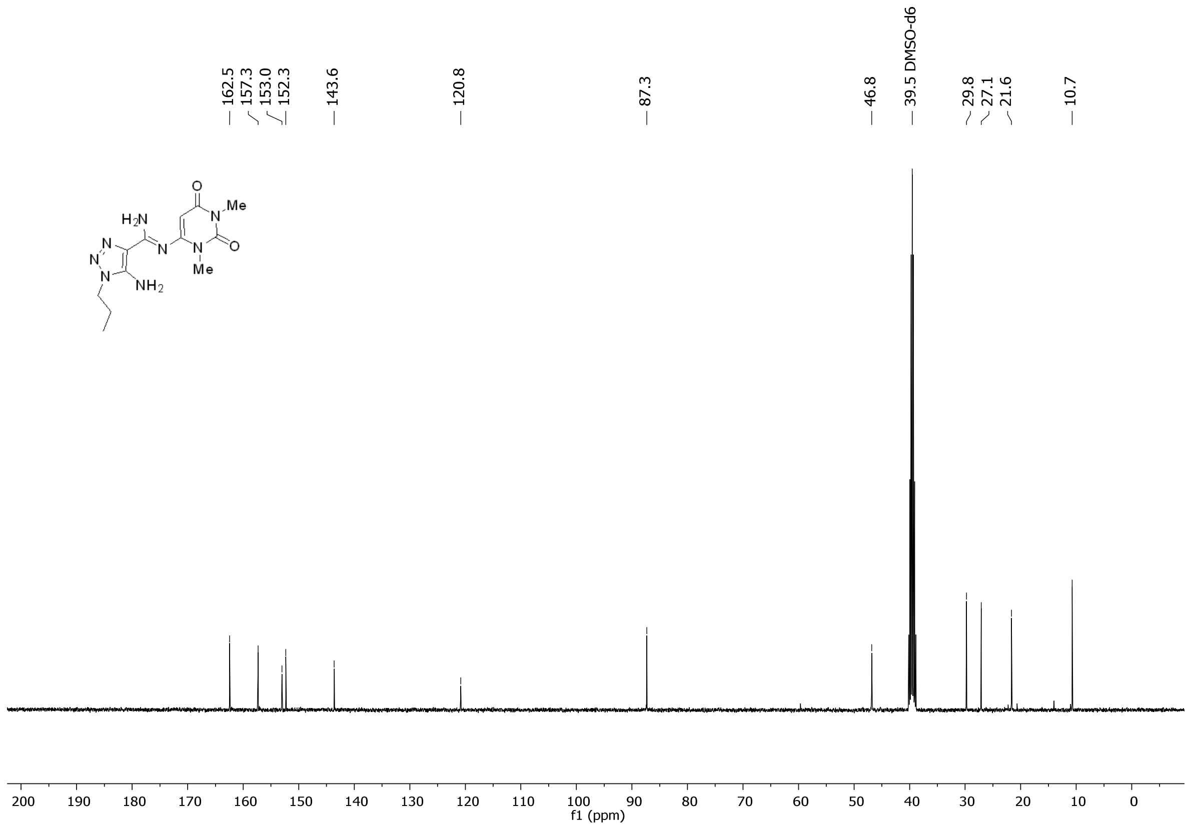

Fig. S10. ^{1}H NMR spectrum (DMSO-d_6) of **3e**

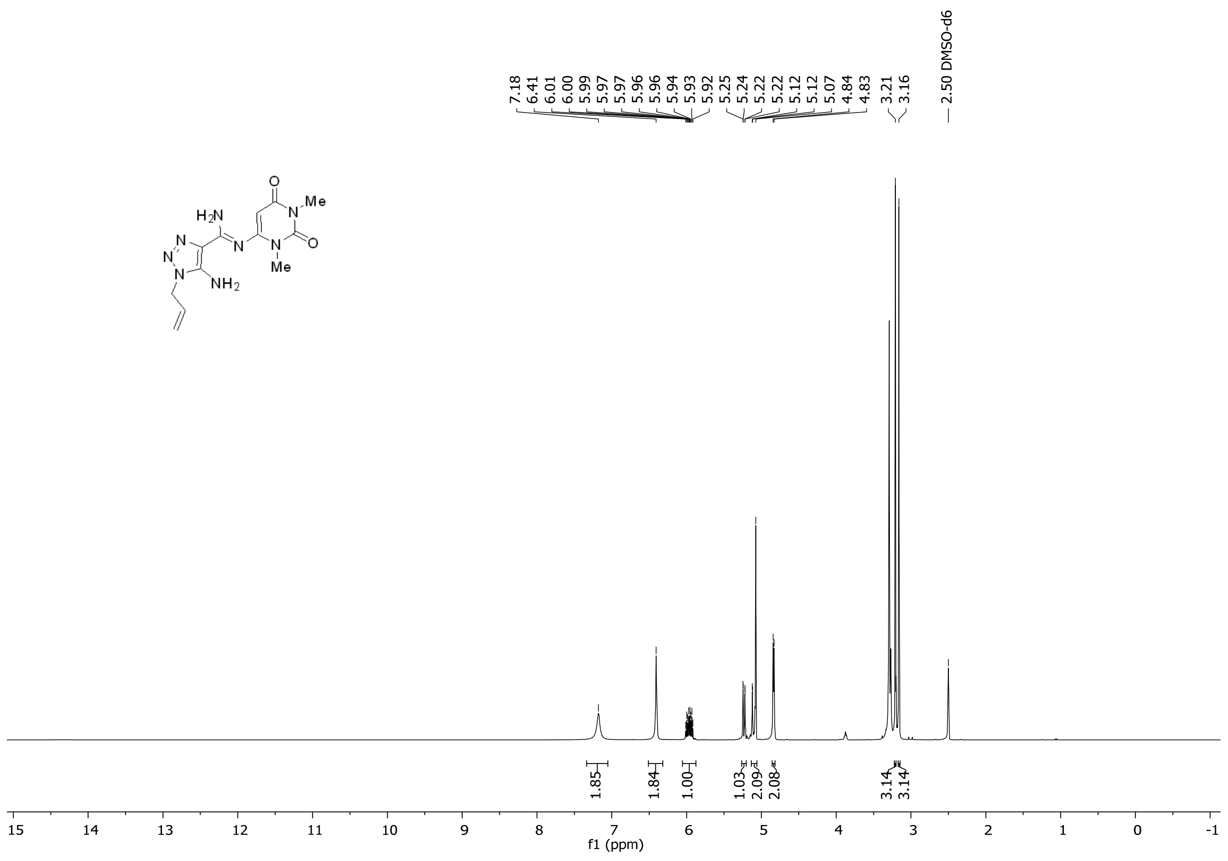

Fig. S11. ^{13}C NMR spectrum (DMSO-d_6) of **3e**

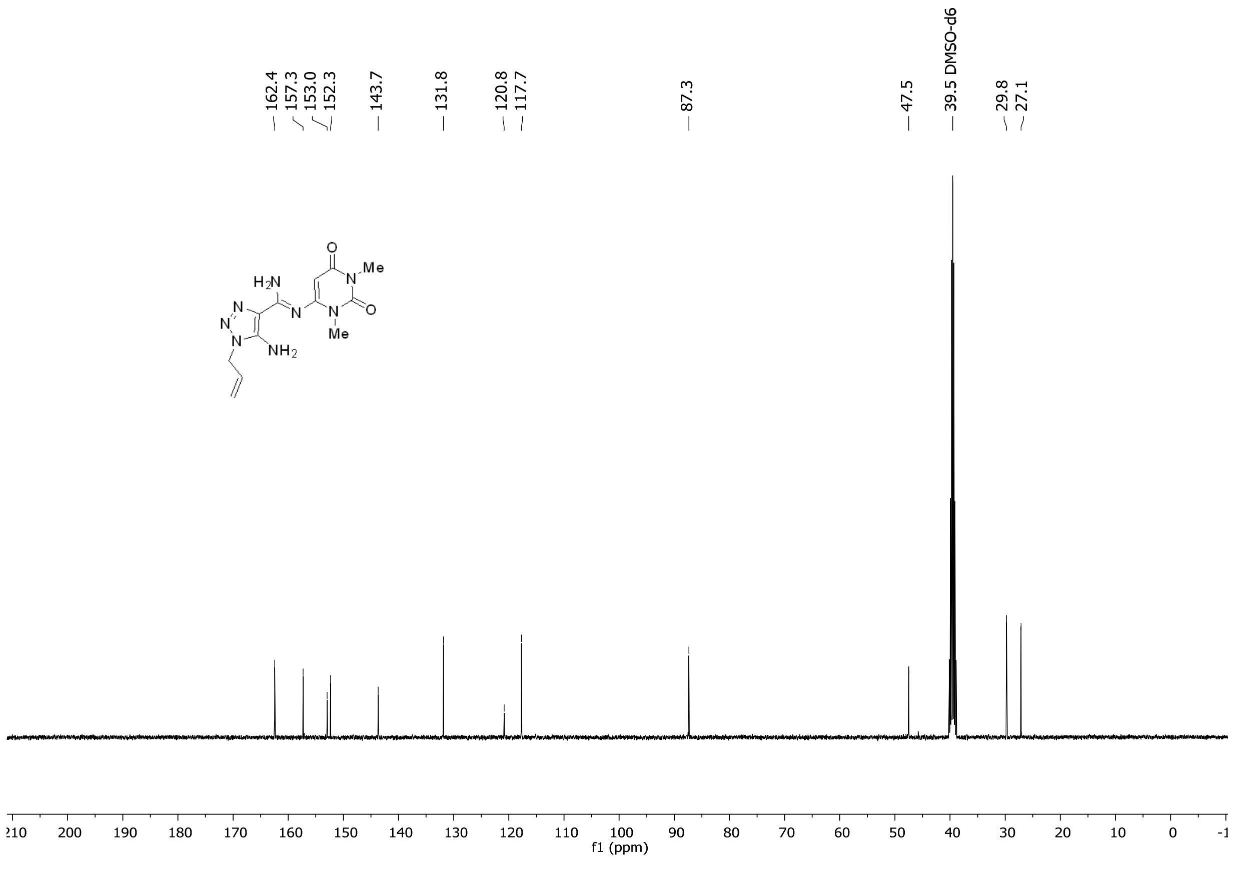

Fig. S12. ^{1}H NMR spectrum (DMSO-d_6) of **3f**

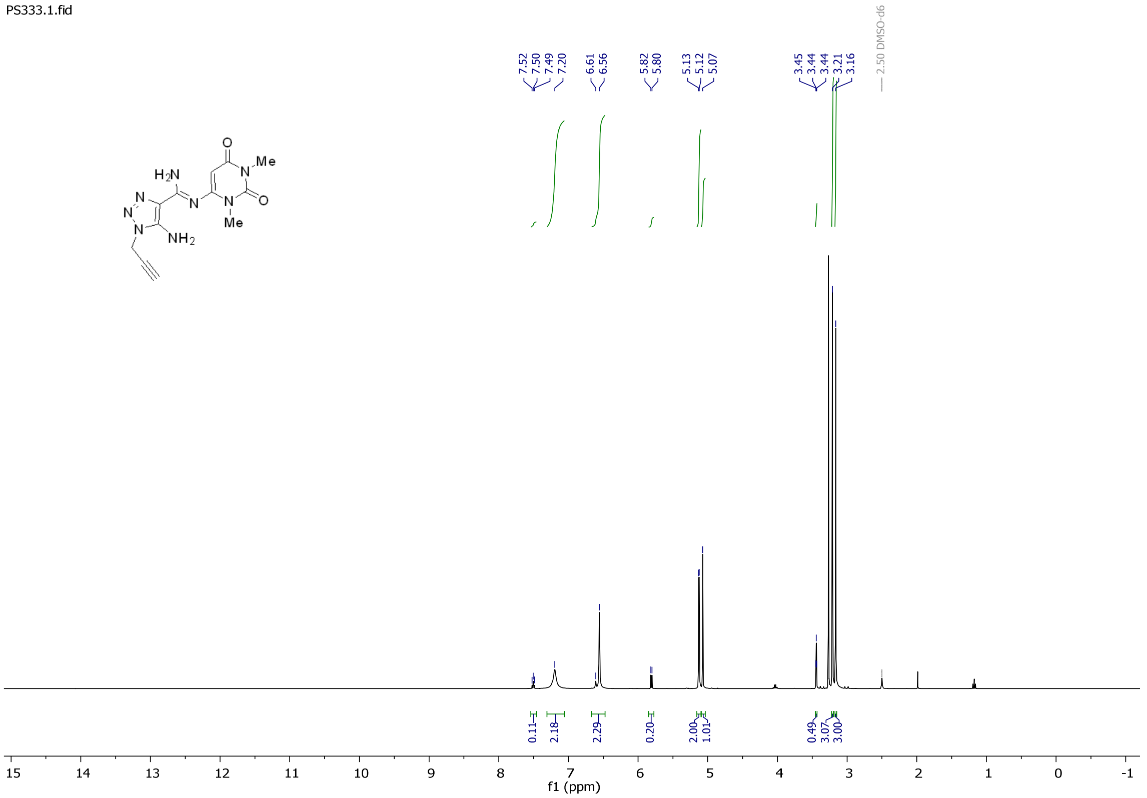

Fig. S13. ^{13}C NMR spectrum (DMSO-d_6) of **3f**

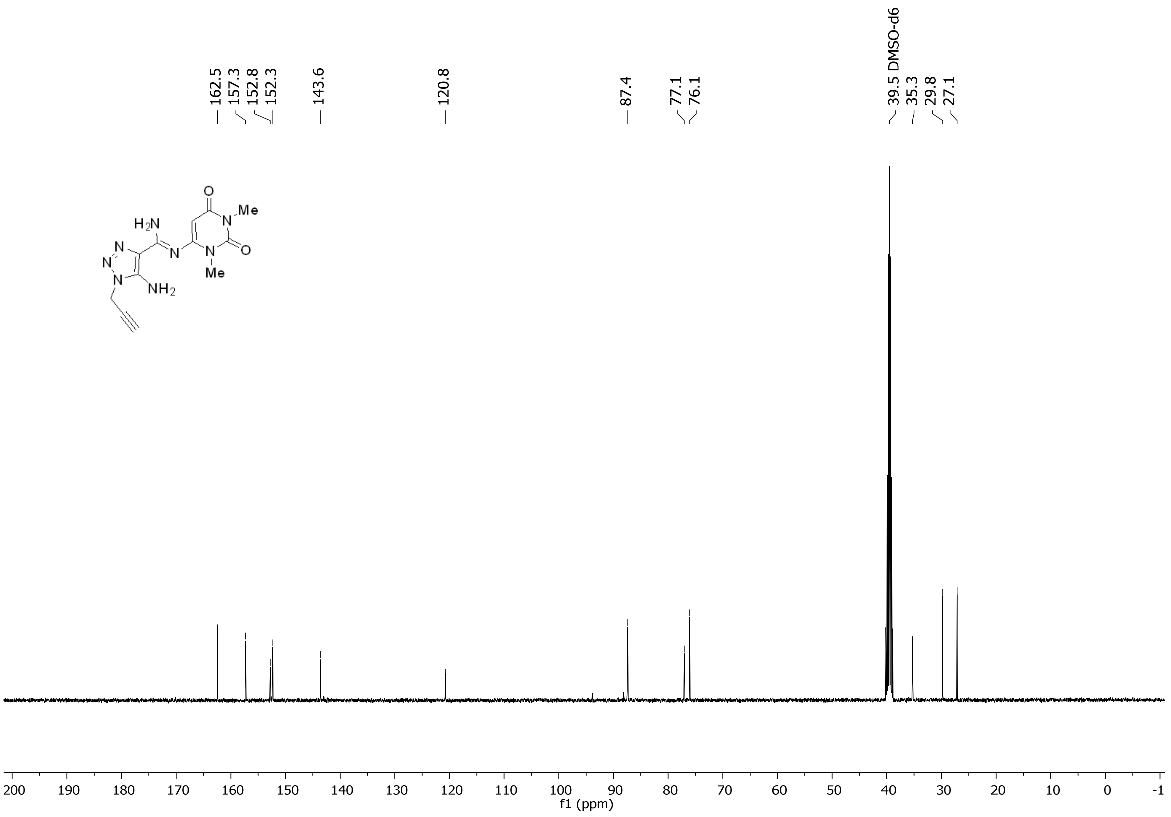

Fig. S14. ^{1}H NMR spectrum (DMSO-d_6) of **3g**

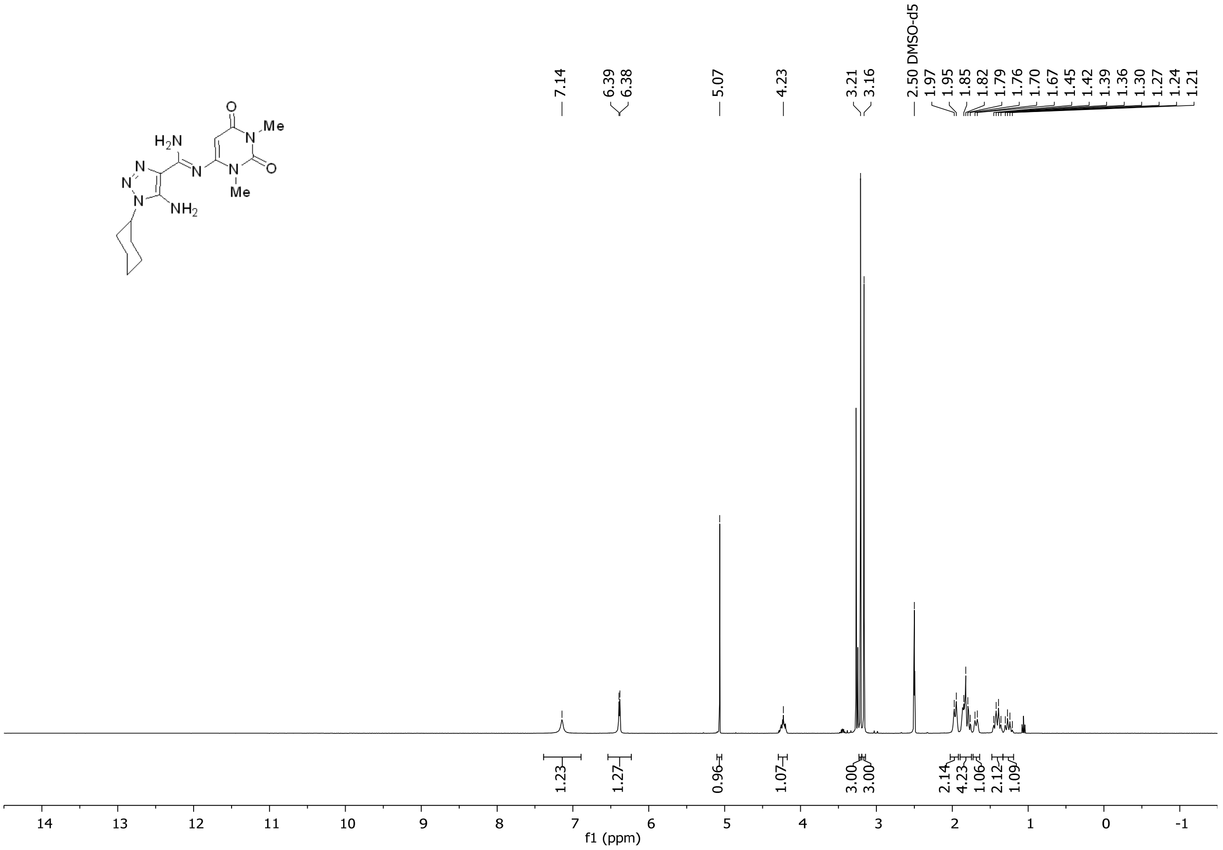

Fig. S15. ^{13}C NMR spectrum (DMSO-d_6) of **3g**

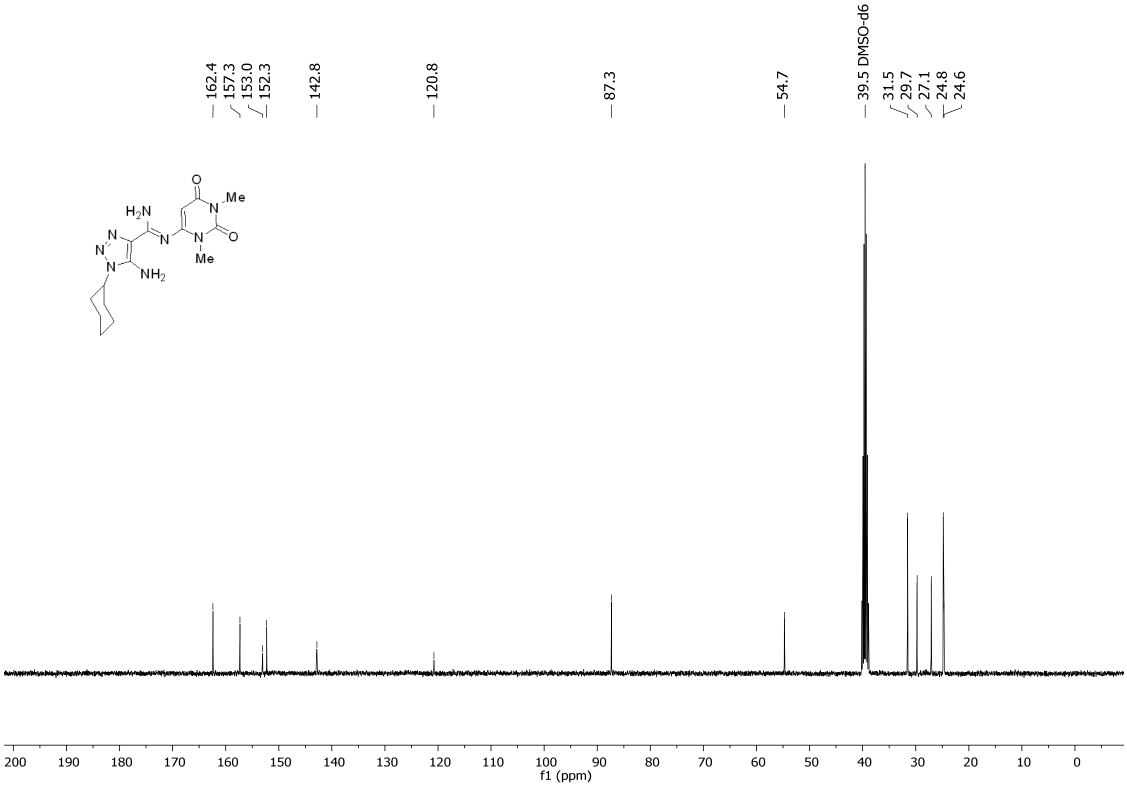

Fig. S16. ^{1}H NMR spectrum (DMSO-d_6) of **3h**

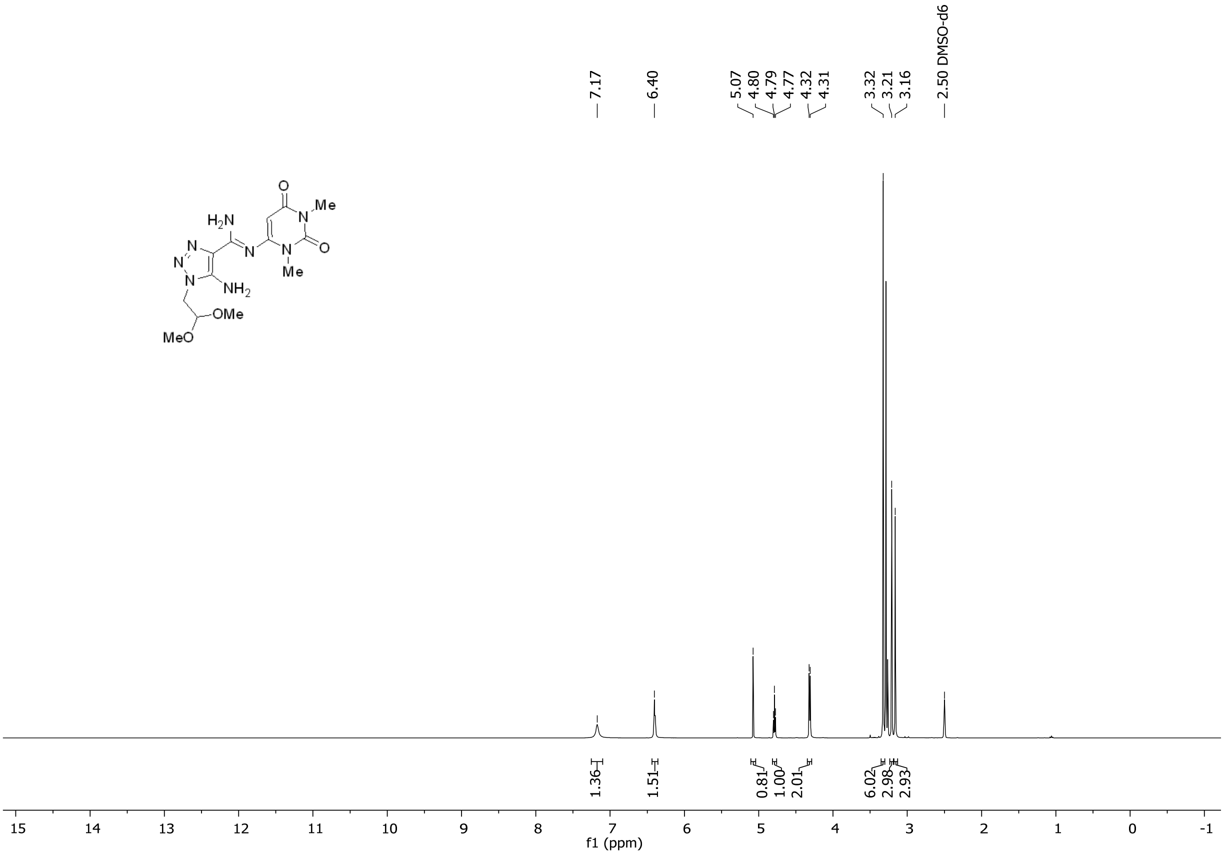

Fig. S17. ^{13}C NMR spectrum (DMSO-d_6) of **3h**

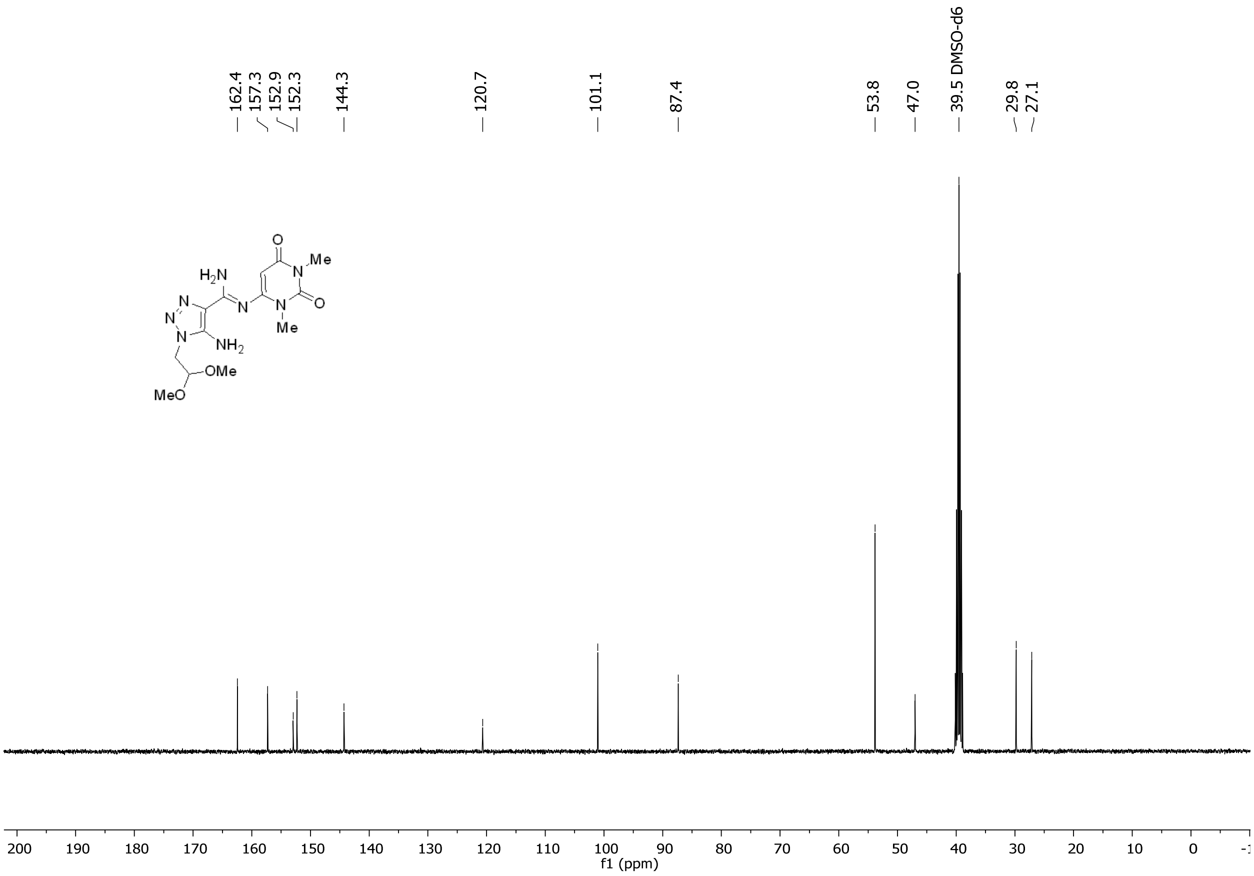

Fig. S18. ^{1}H NMR spectrum (DMSO-d_6) of **3i**

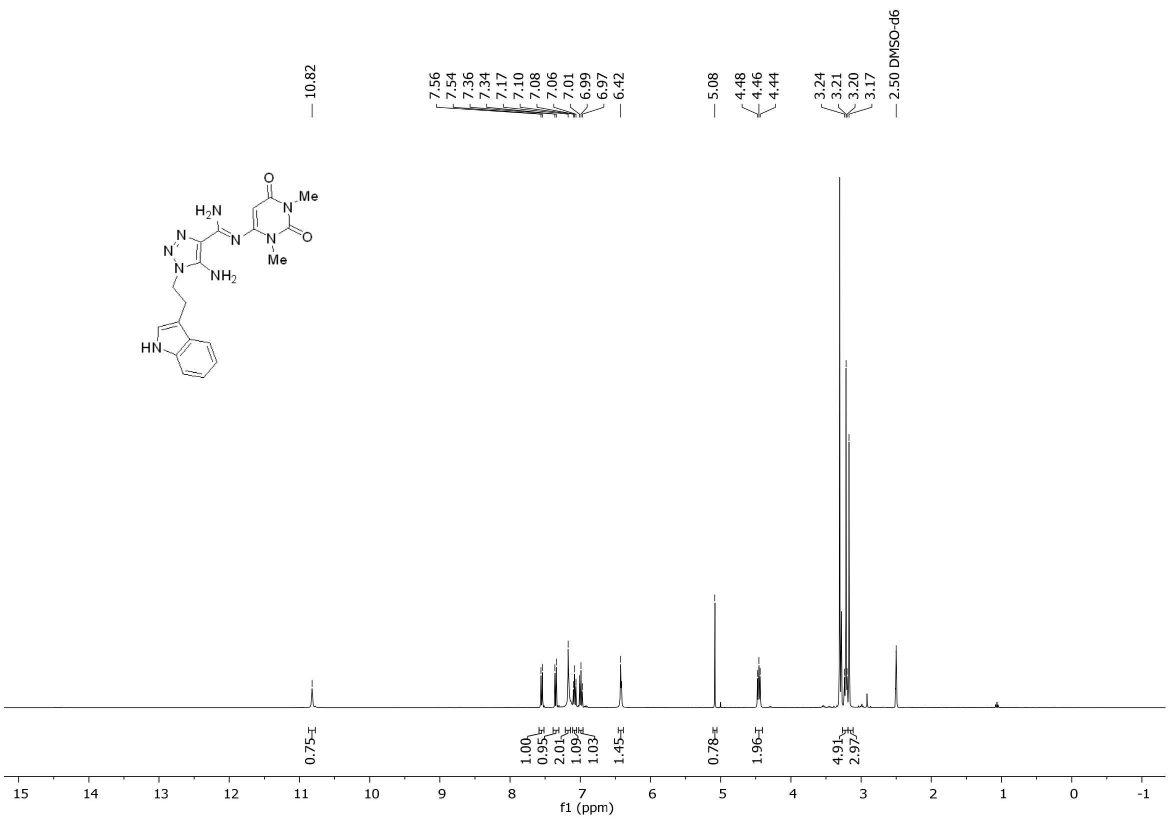

Fig. S19. ^{13}C NMR spectrum (DMSO-d_6) of **3i**

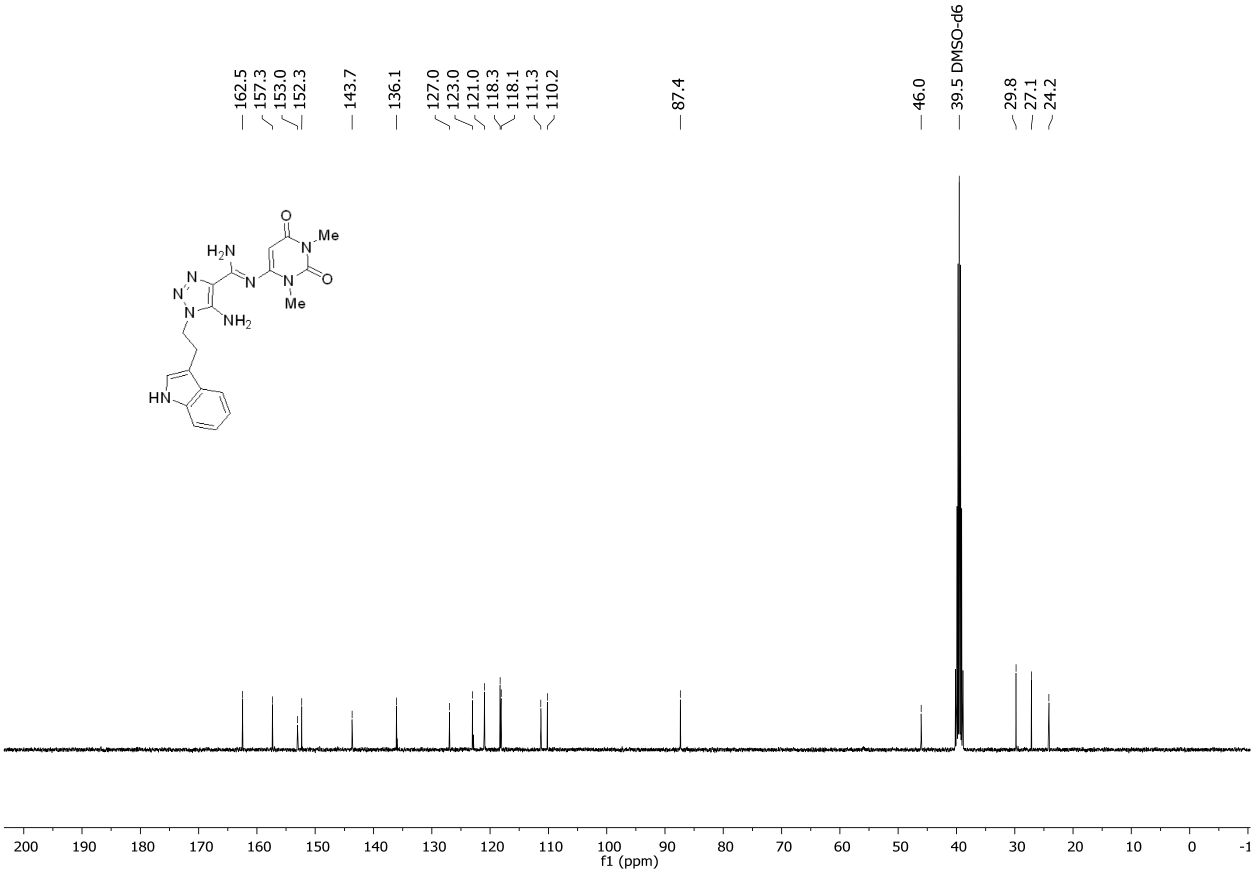

Fig. 19A. HMBC spectrum (DMSO-d_6) of **3r**

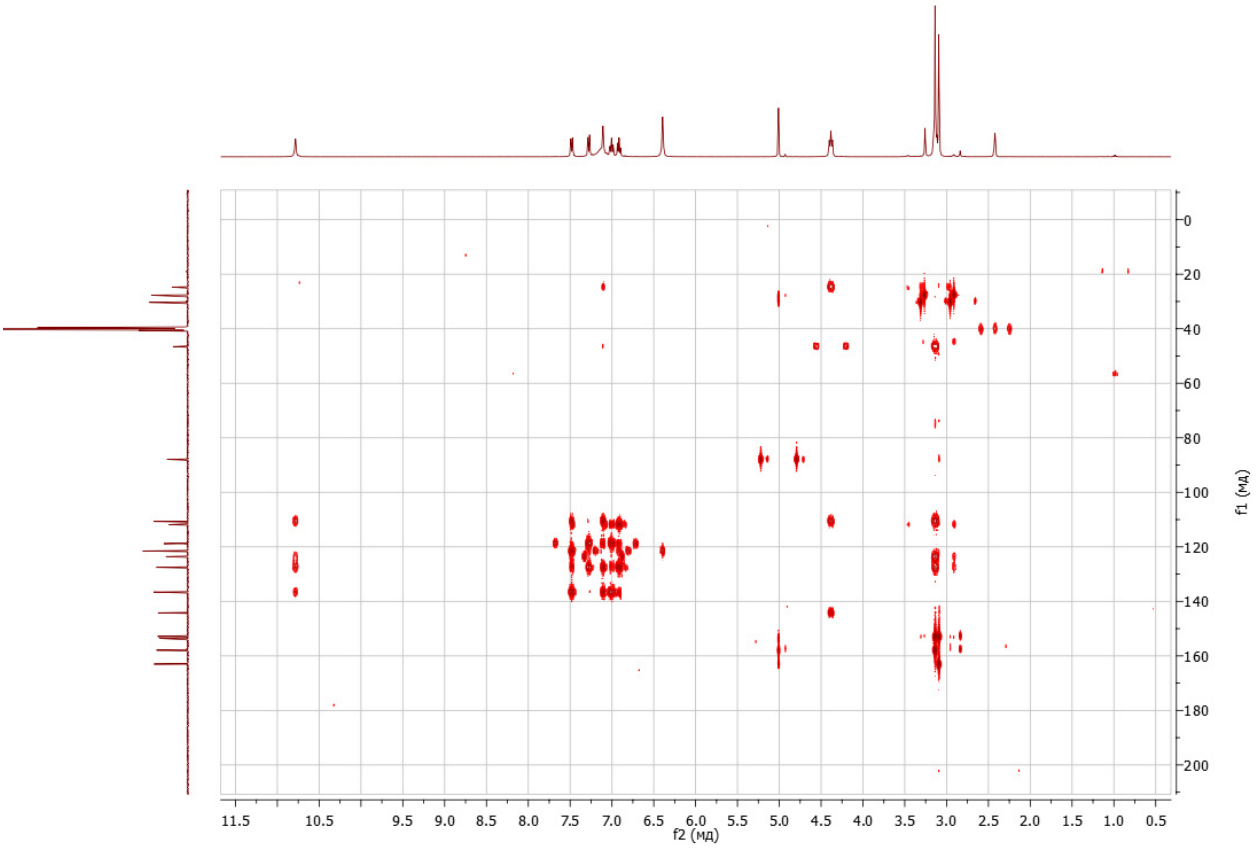

Fig. S20. ^{1}H NMR spectrum (DMSO-d_6) of **3j**

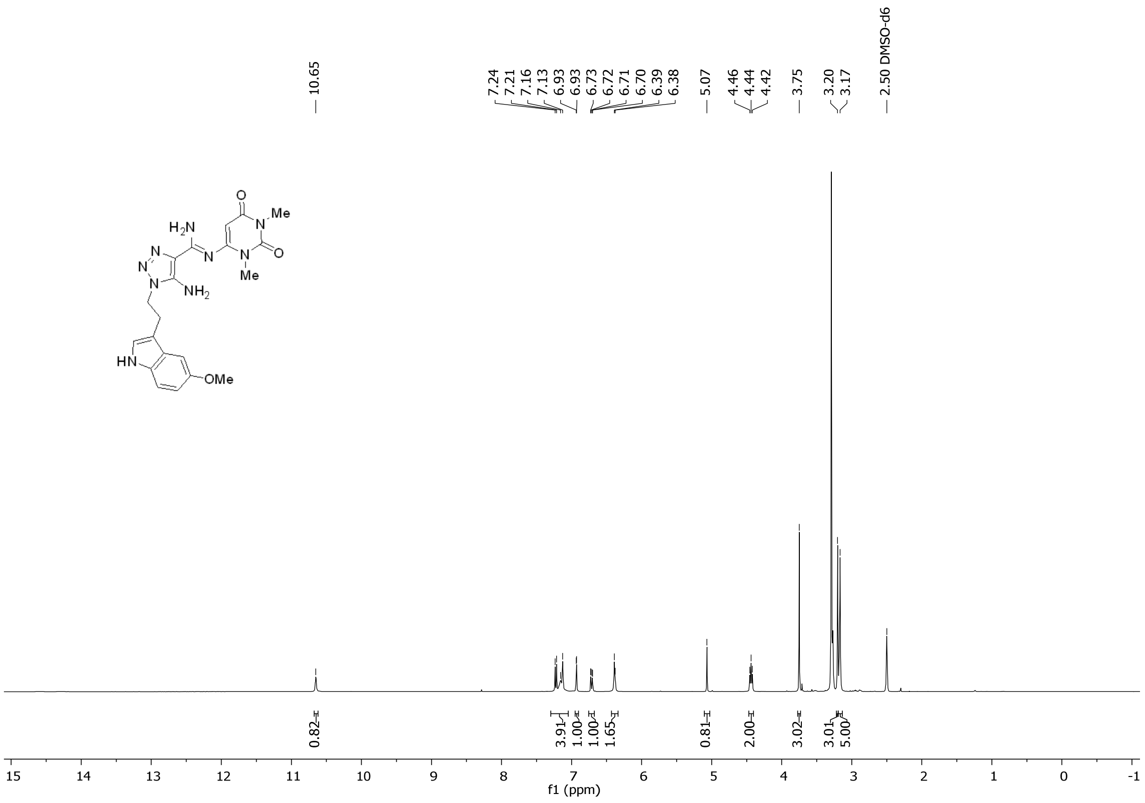

Fig. S21. ^{13}C NMR spectrum (DMSO-d_6) of **3j**

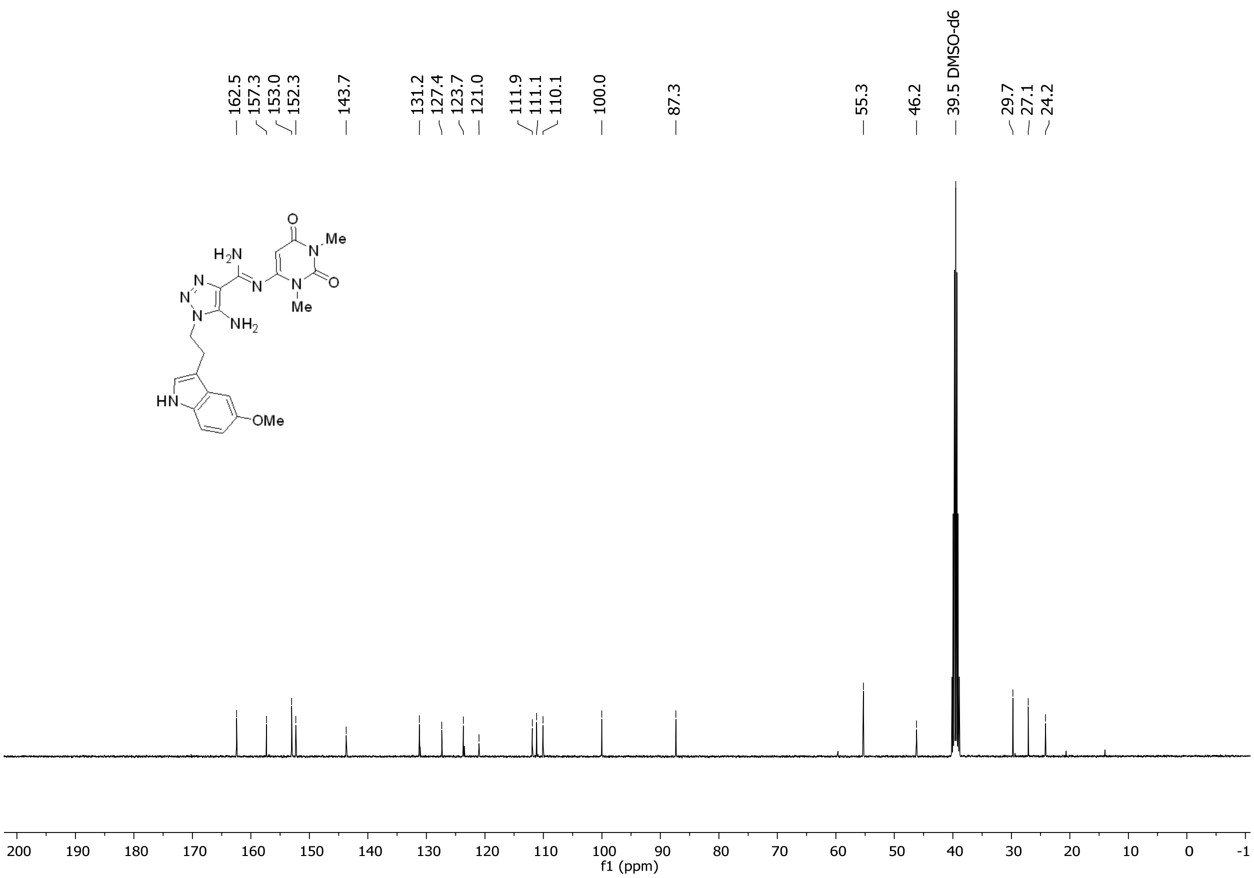

Fig. S22. ^{1}H NMR spectrum (DMSO-d_6) of **3k**

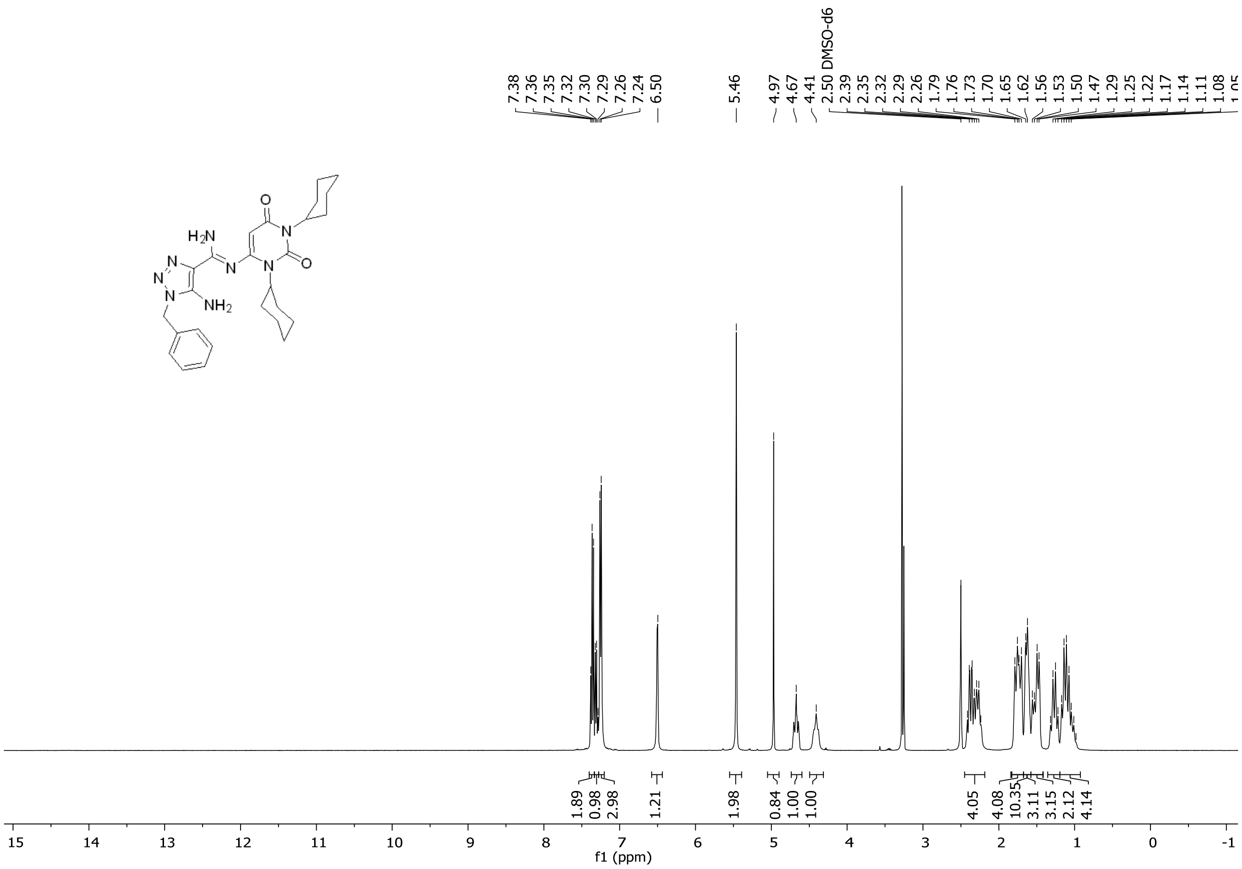

Fig. S23. ^{13}C NMR spectrum (DMSO-d_6) of **3k**

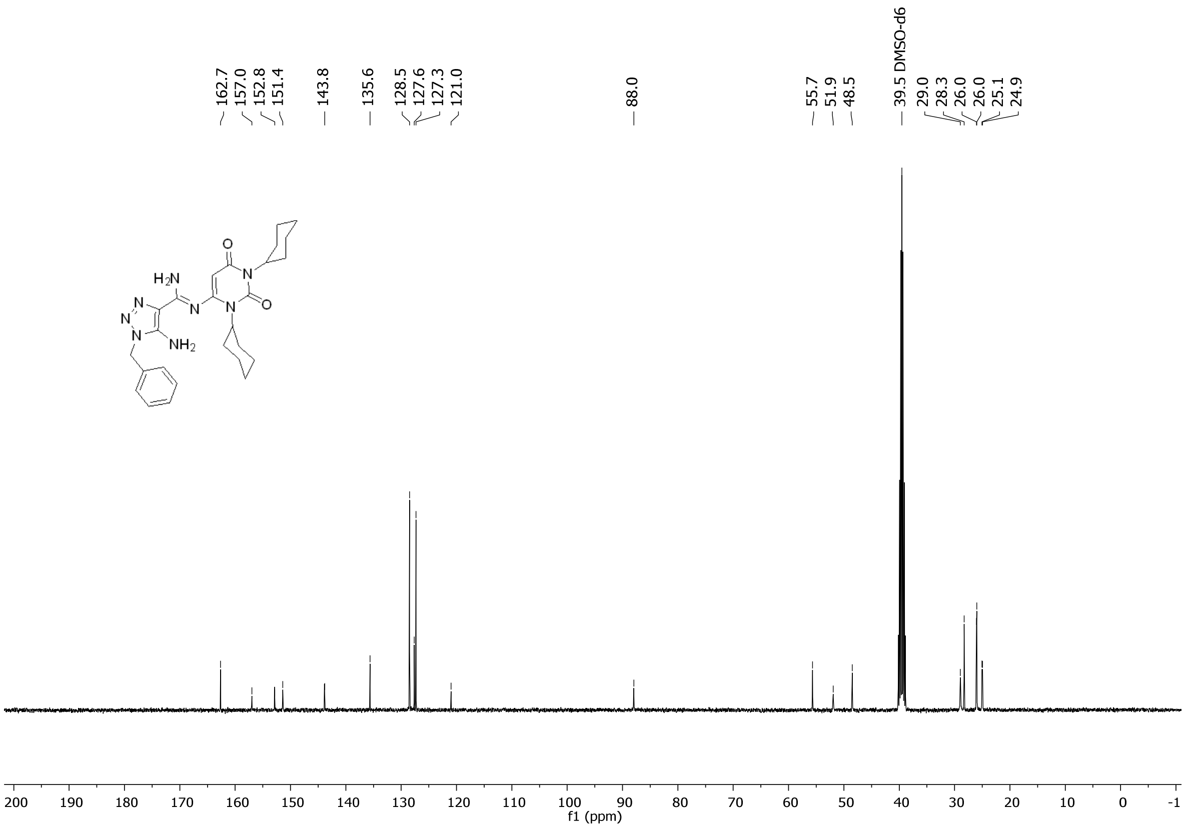

Fig. 23A. HMBC spectrum (DMSO-d$_6$) of **3k**

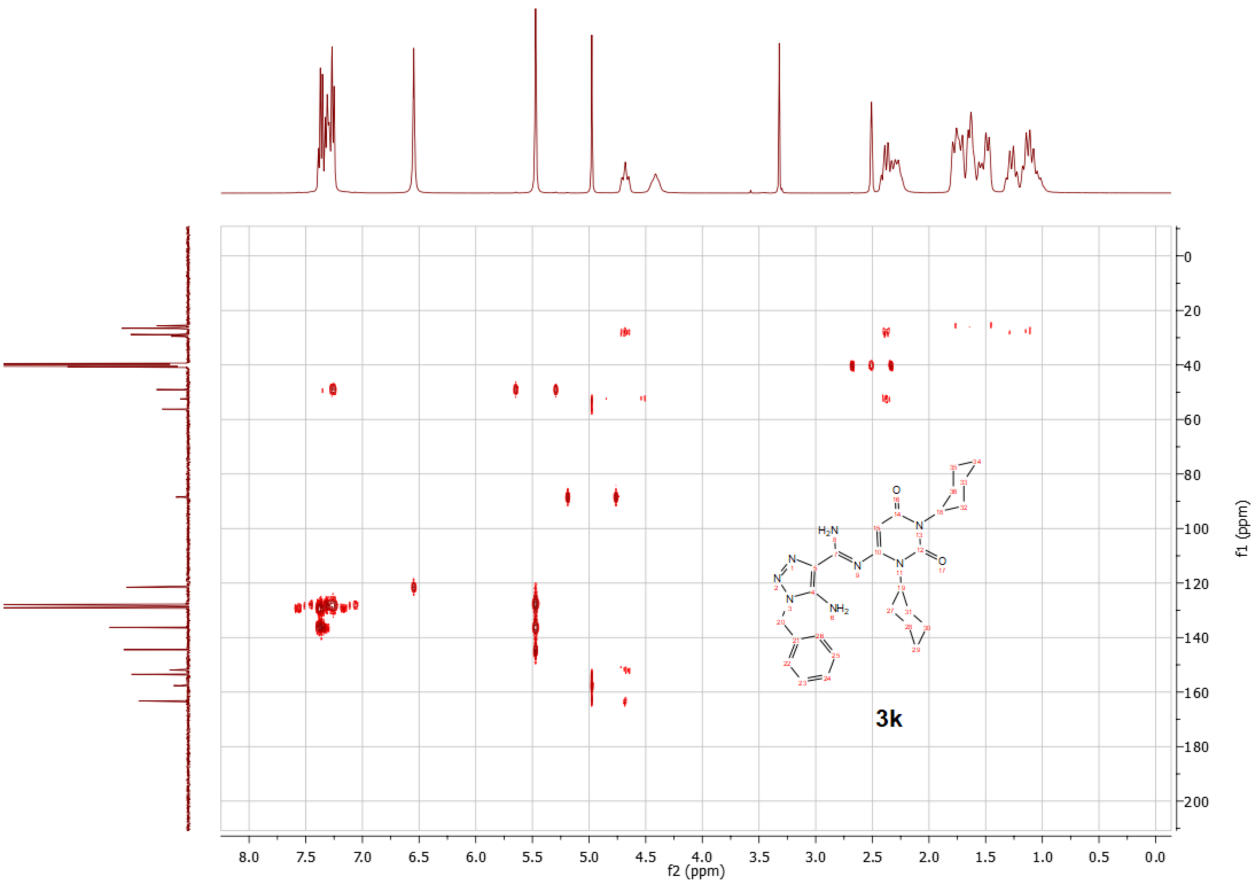

Fig. S24. ^{1}H NMR spectrum (DMSO-d_6) of **3l**

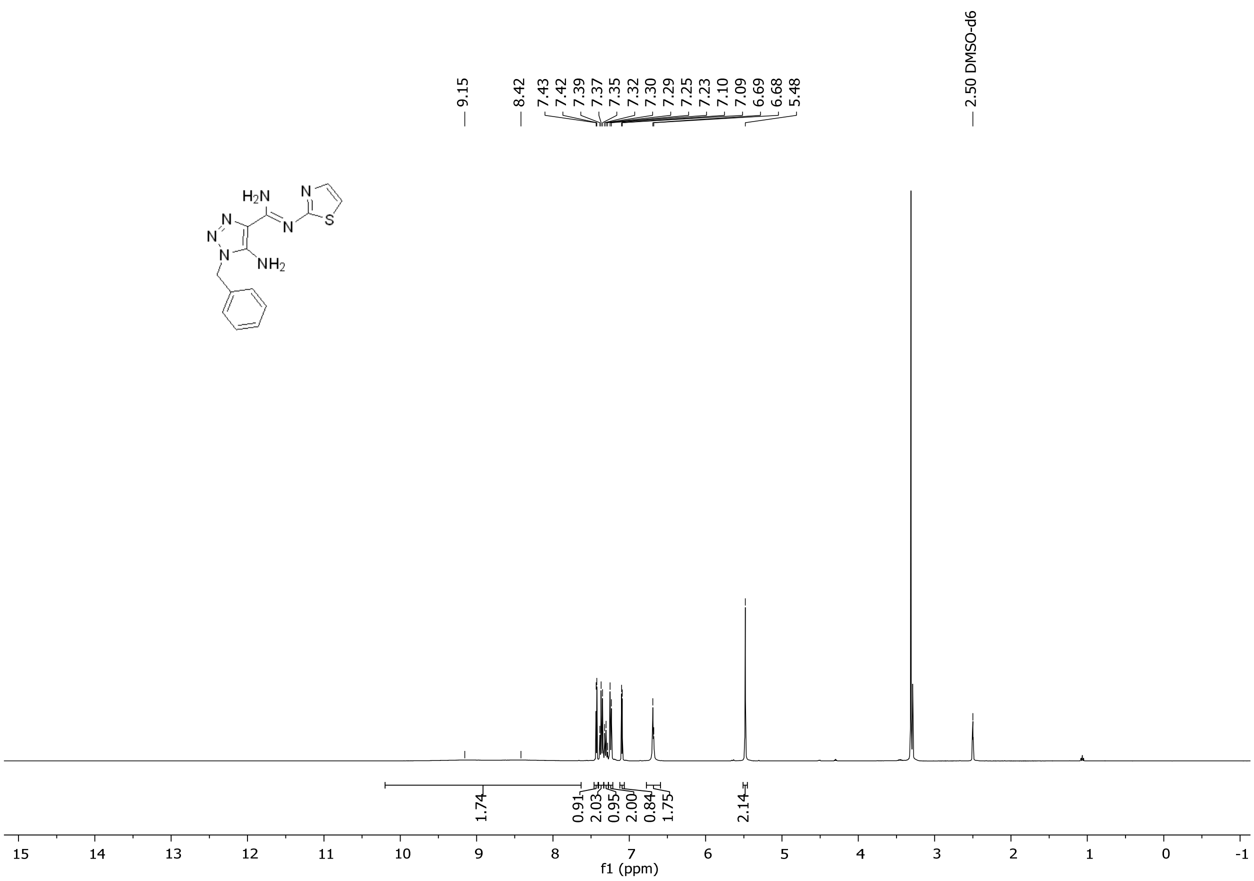

Fig. S25. ^{13}C NMR spectrum (DMSO-d_6) of **3l**

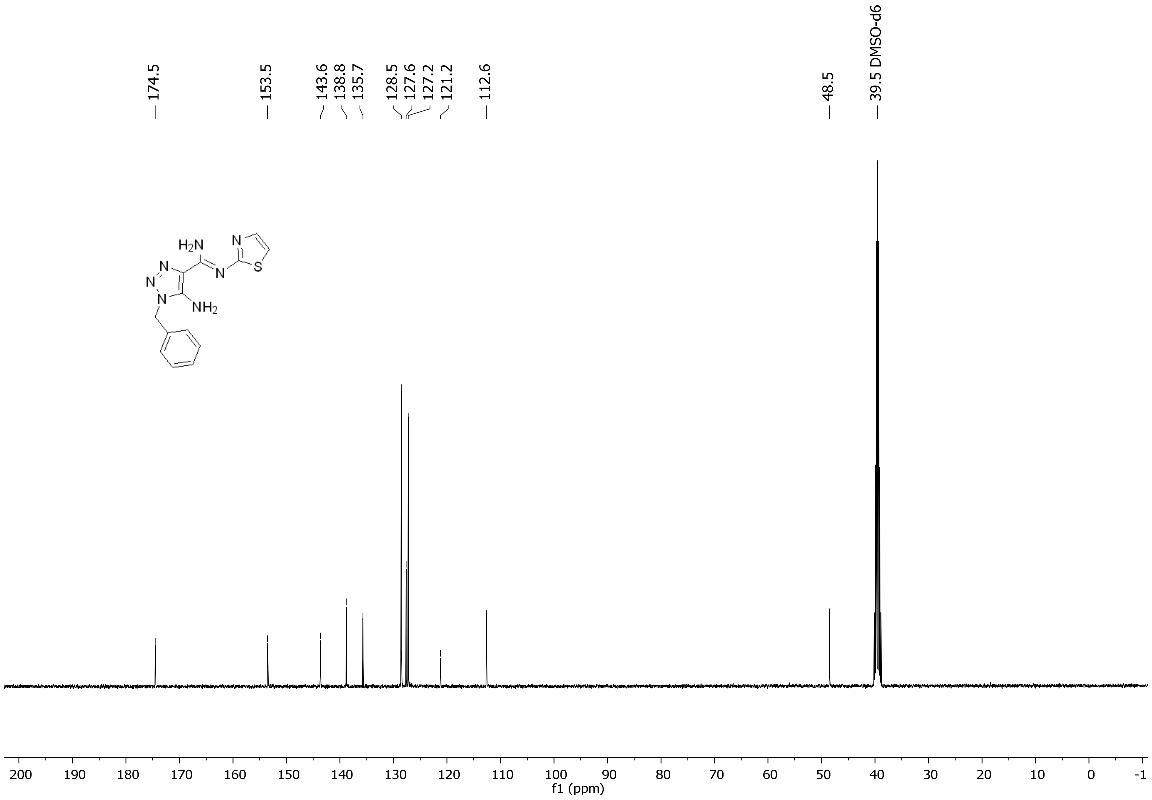

Fig. S26. ^{1}H NMR spectrum (DMSO-d_6) of **3m**

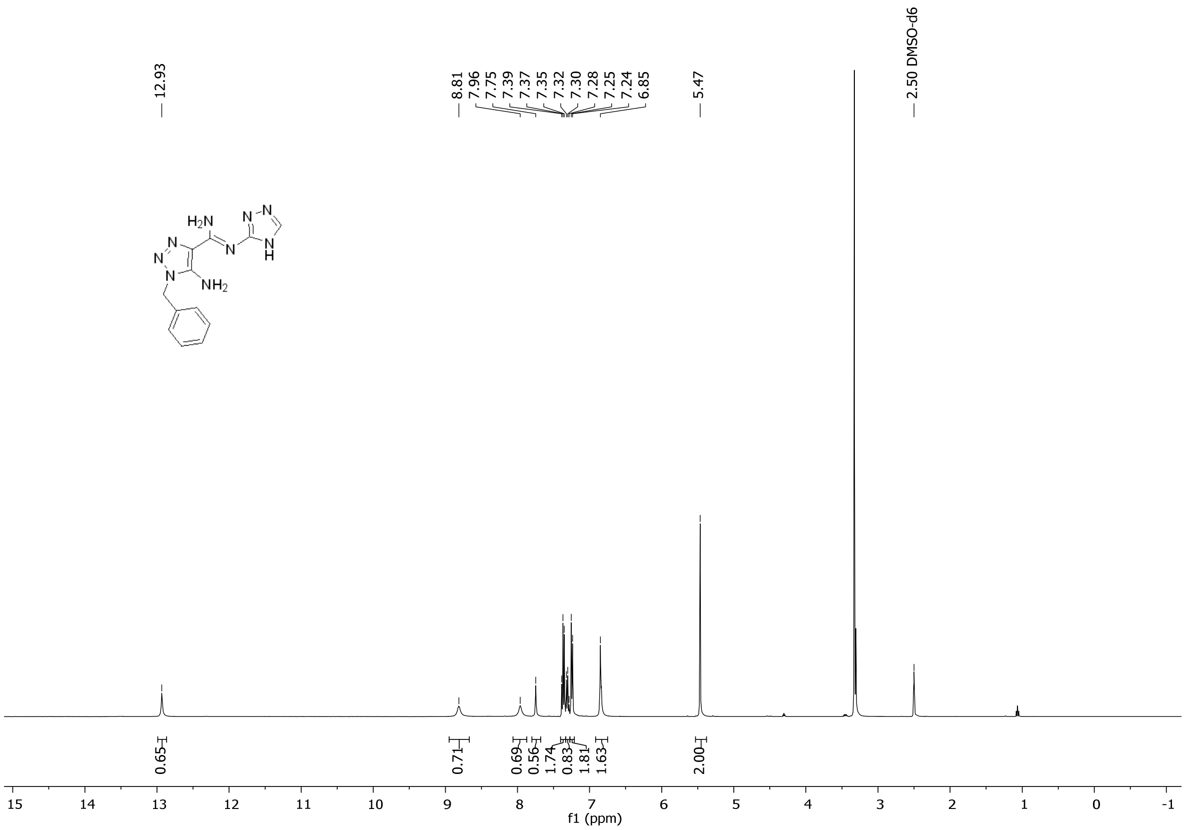

Fig. S27. ^{13}C NMR spectrum (DMSO-d_6) of **3m**

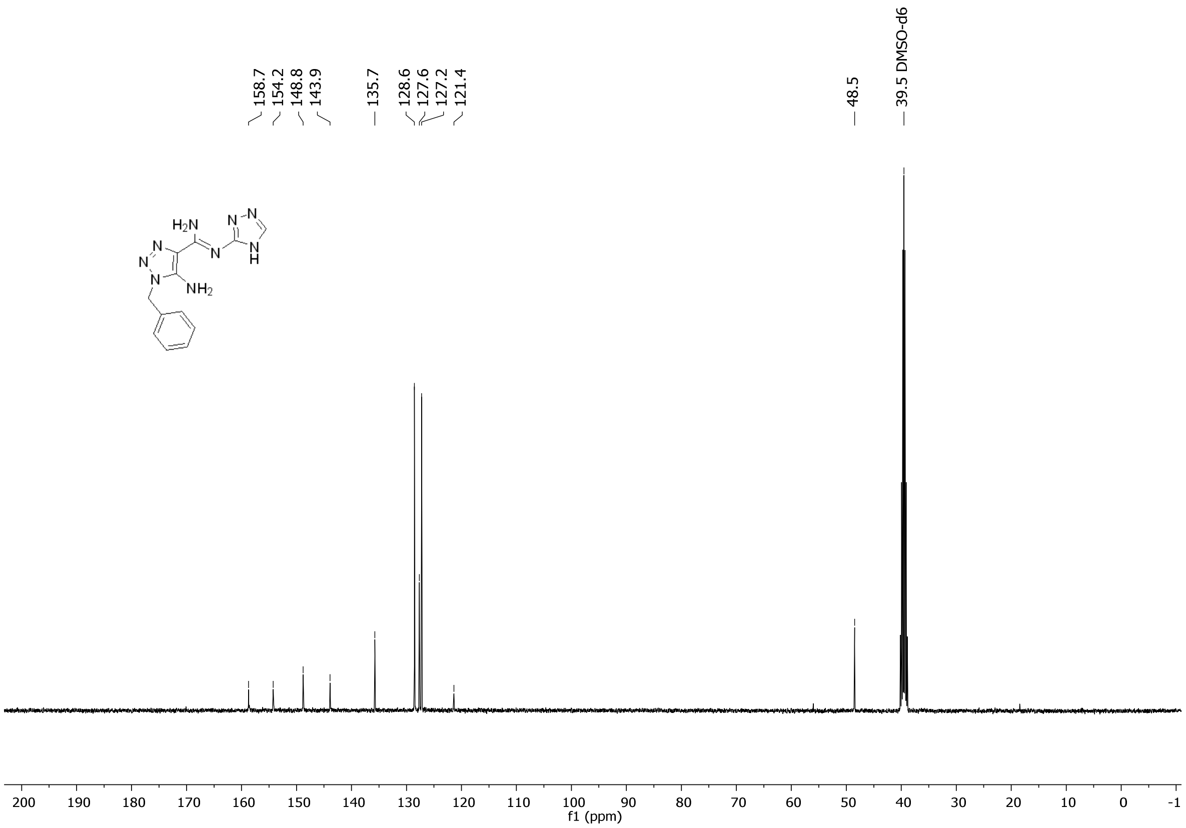

Fig. S28. ^{1}H NMR spectrum (DMSO-d_6) of **3n**

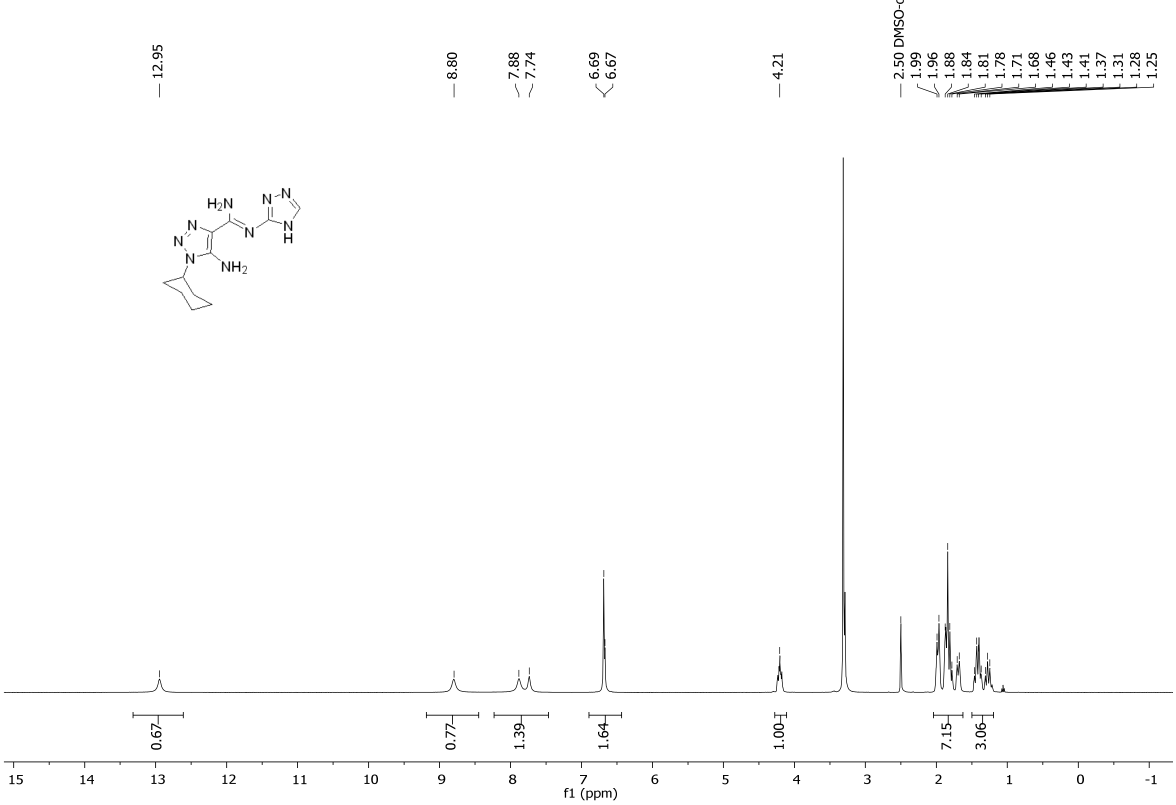

Fig. S29. ^{13}C NMR spectrum (DMSO-d_6) of **3n**

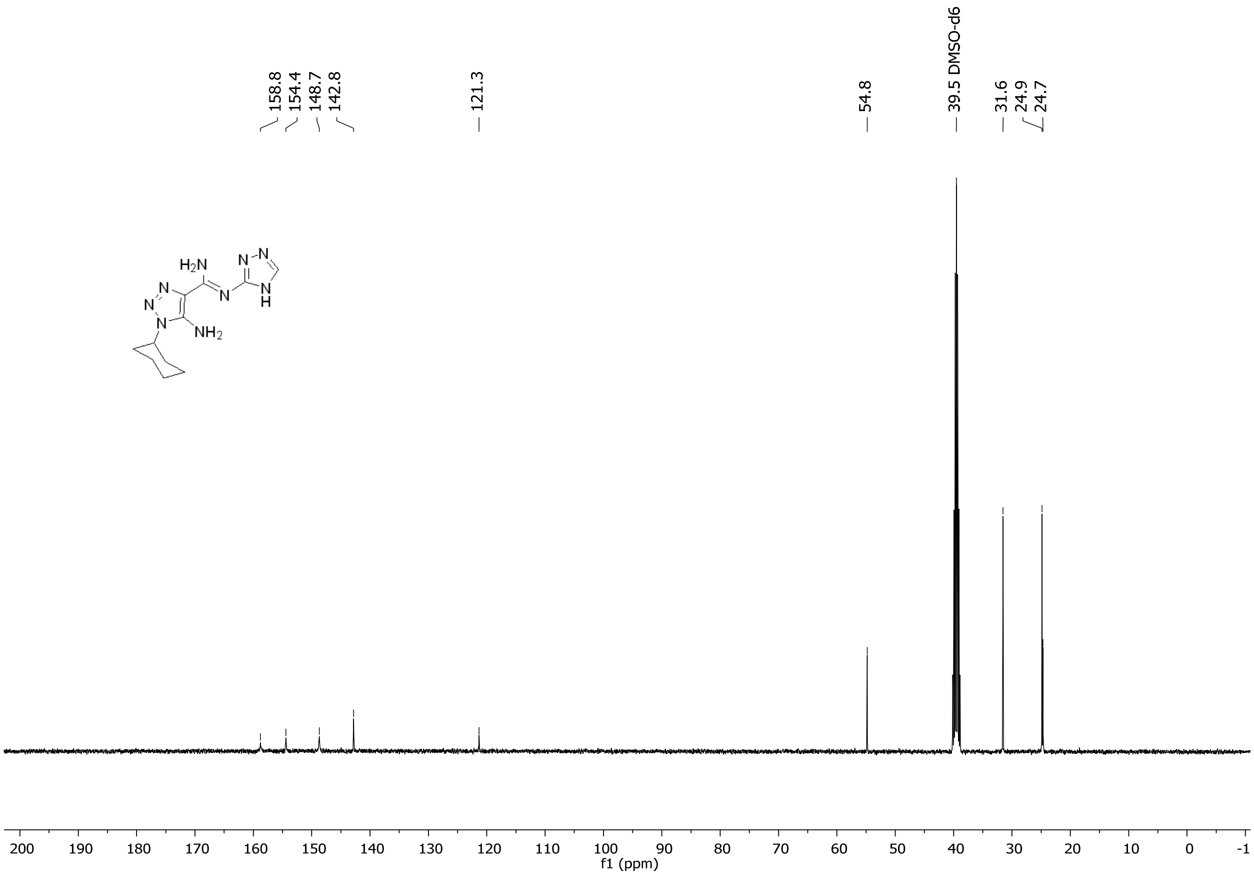

Fig. S30. ^{1}H NMR spectrum (DMSO-d_6) of **3o**

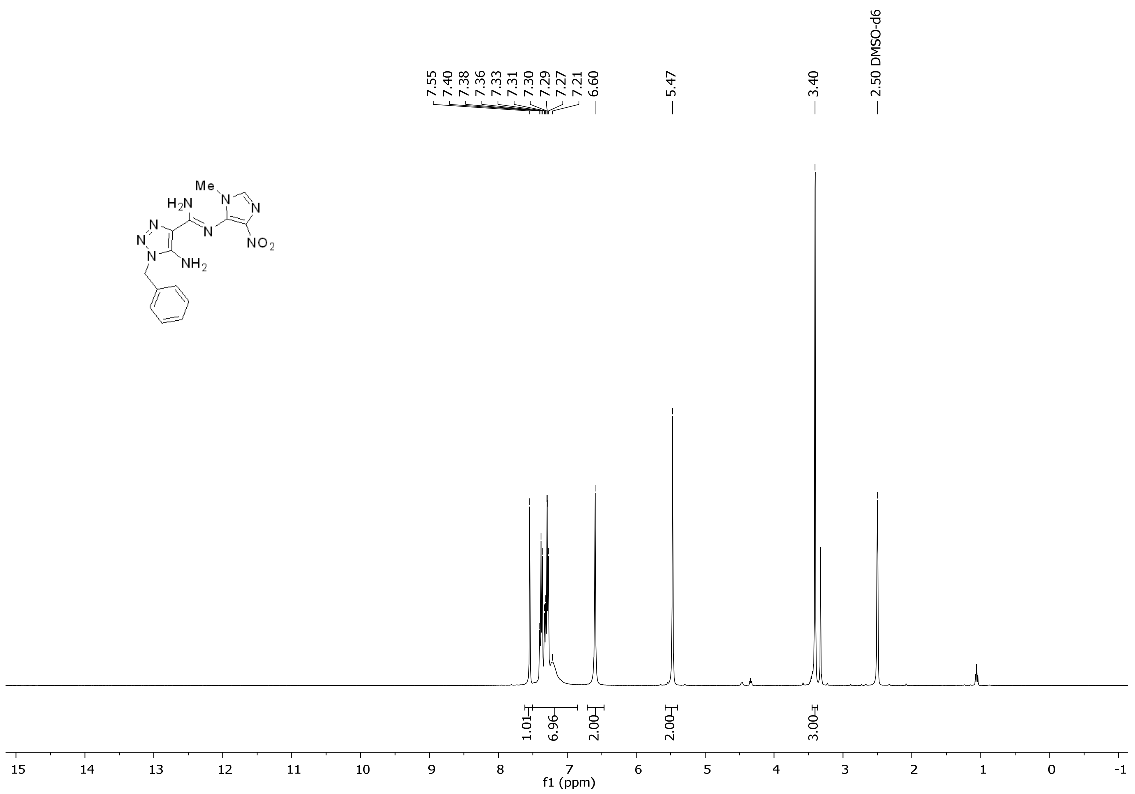

Fig. S31. ^{13}C NMR spectrum (DMSO-d_6) of **3o**

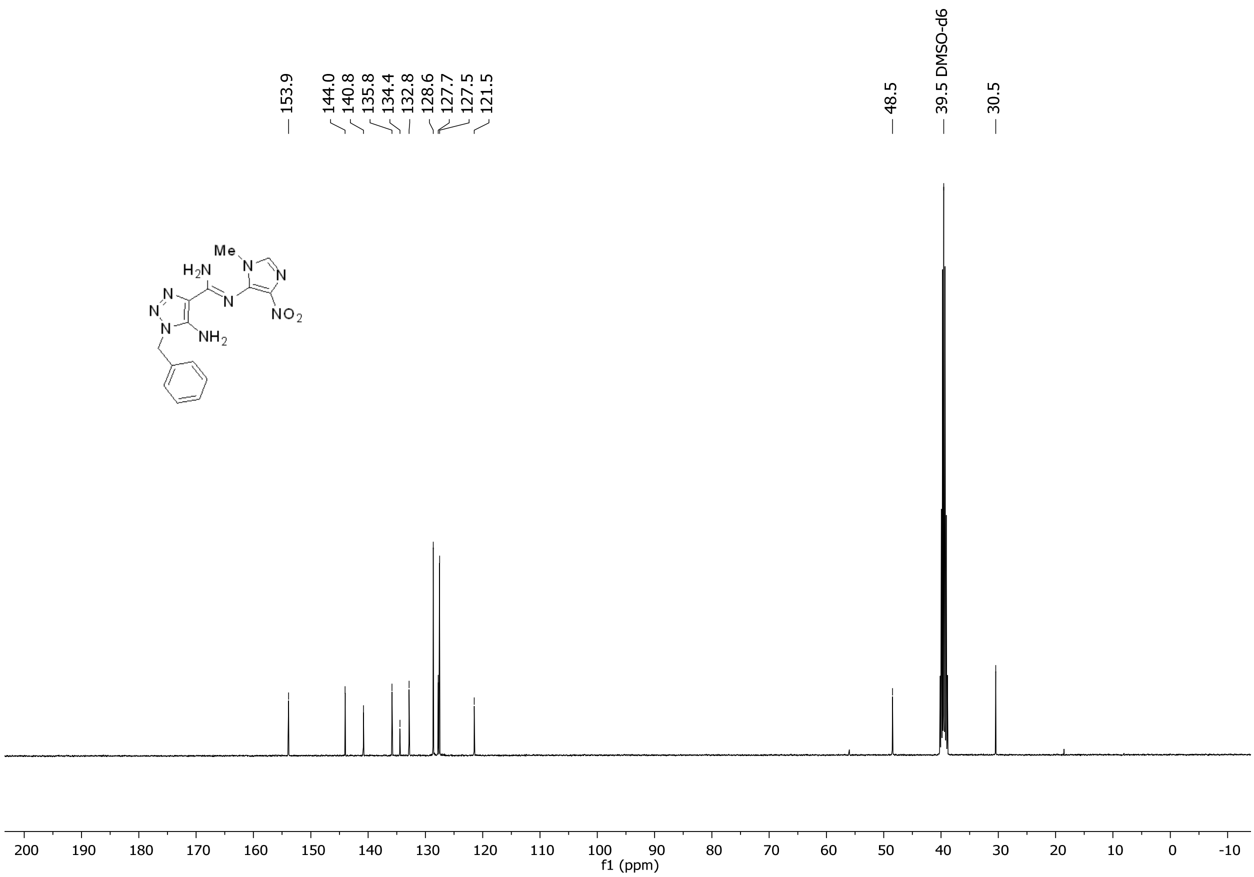

Fig. S32. ^{1}H NMR spectrum (DMSO-*d*6) of **3p**

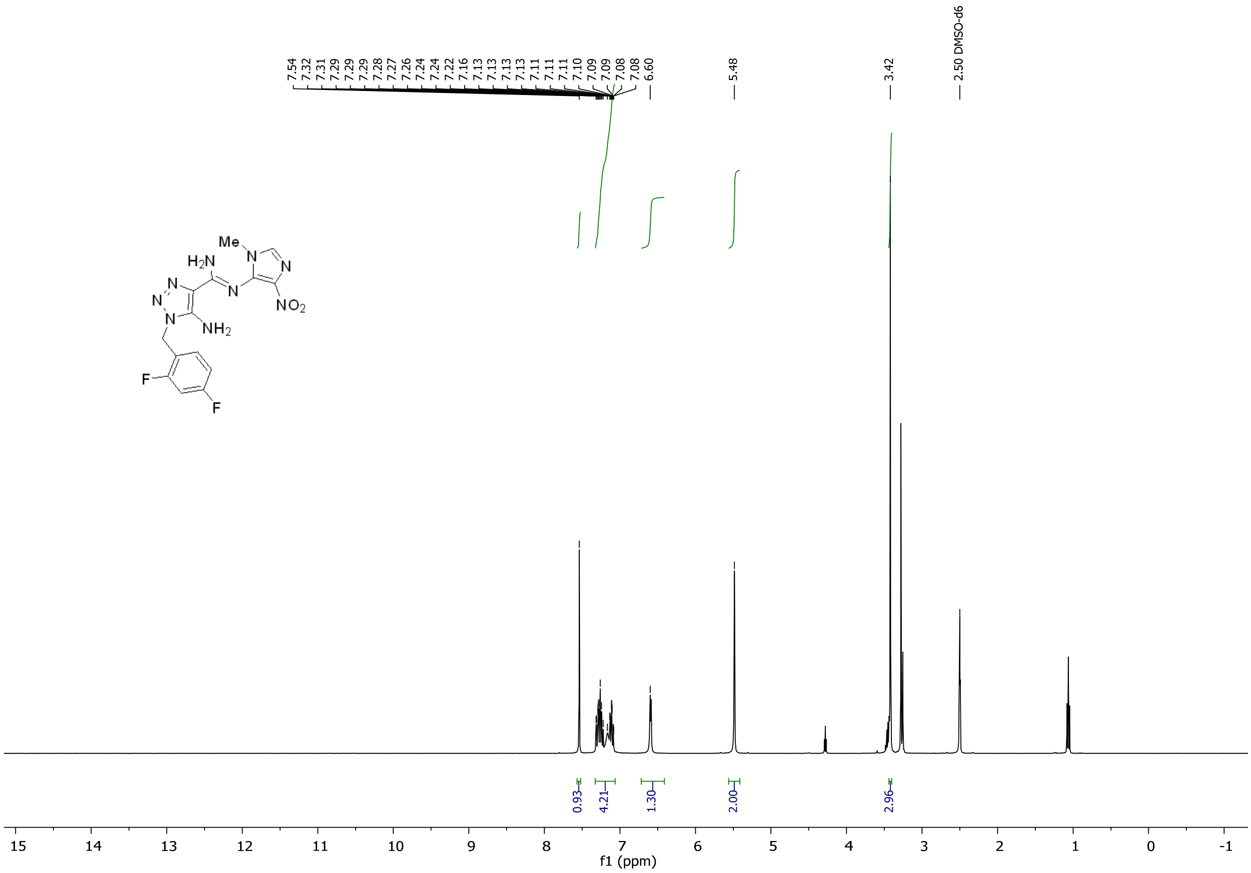

Fig. S33. ^{13}C NMR spectrum (DMSO-*d*6) of **3p**

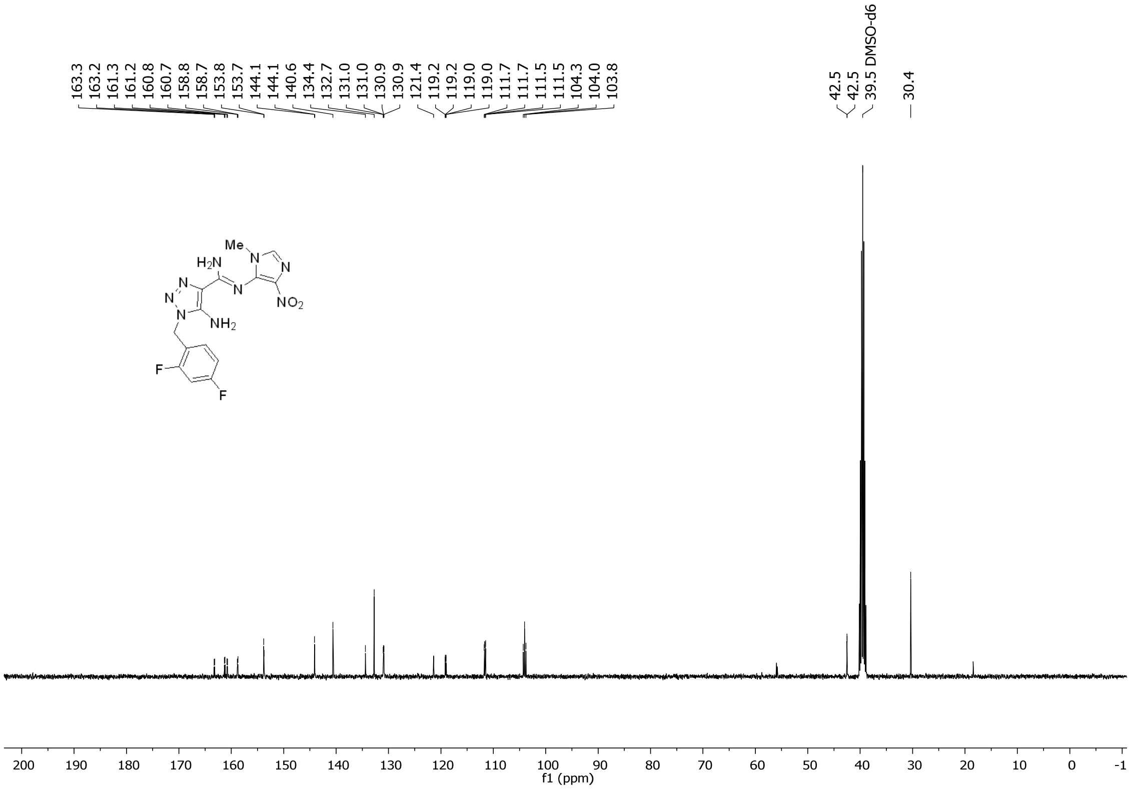

Fig. S34. ^{19}F NMR spectrum (DMSO-d_6) of **3p**

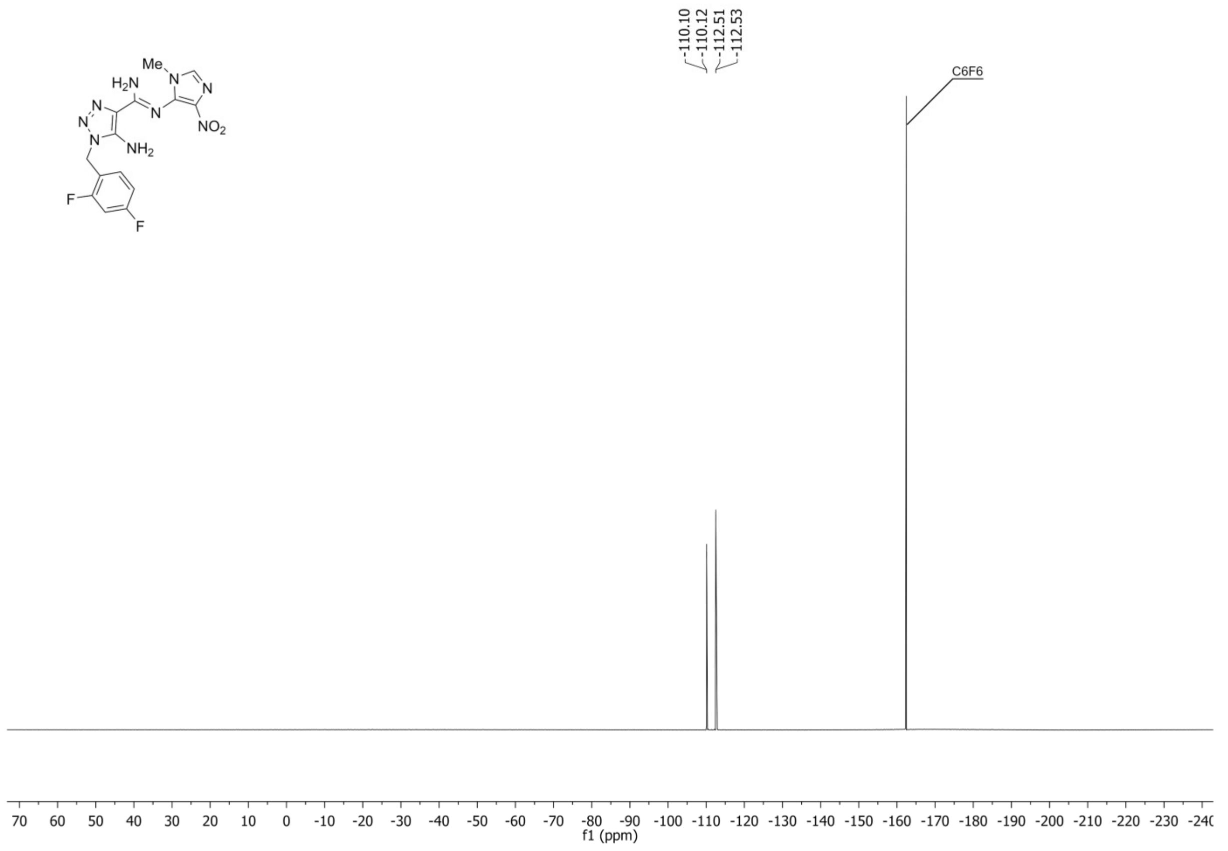

Fig. S35. ^{1}H NMR spectrum (DMSO-d_6) of **3q**

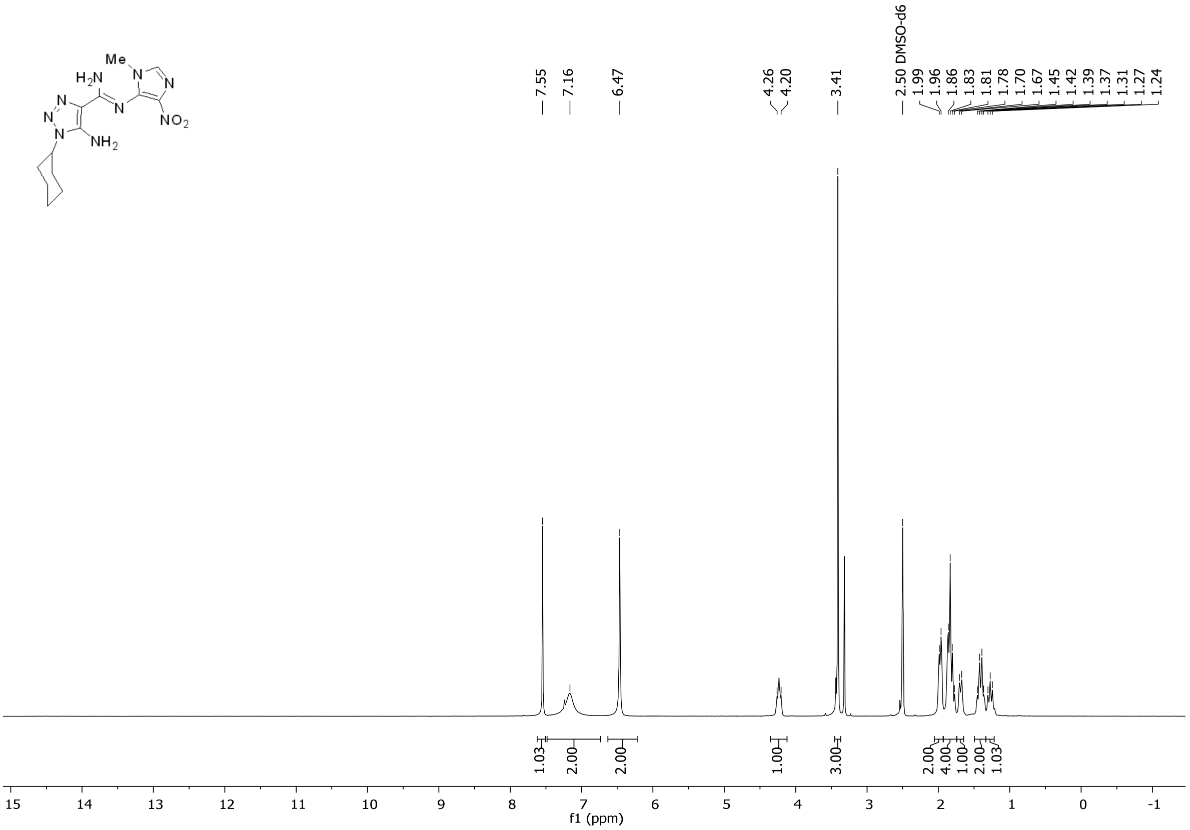

Fig. S36. ^{13}C NMR spectrum (DMSO-d_6) of **3q**

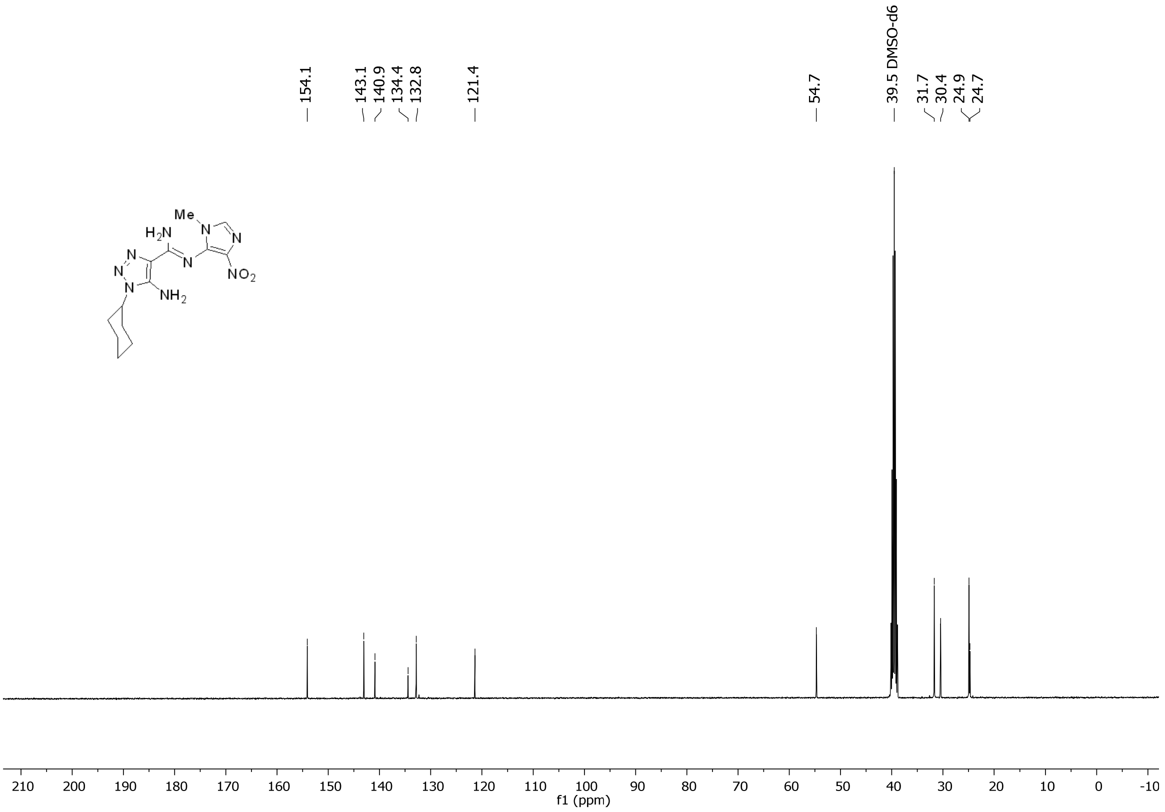

Fig. S37. ^{1}H NMR spectrum (DMSO-d_6) of **3r**

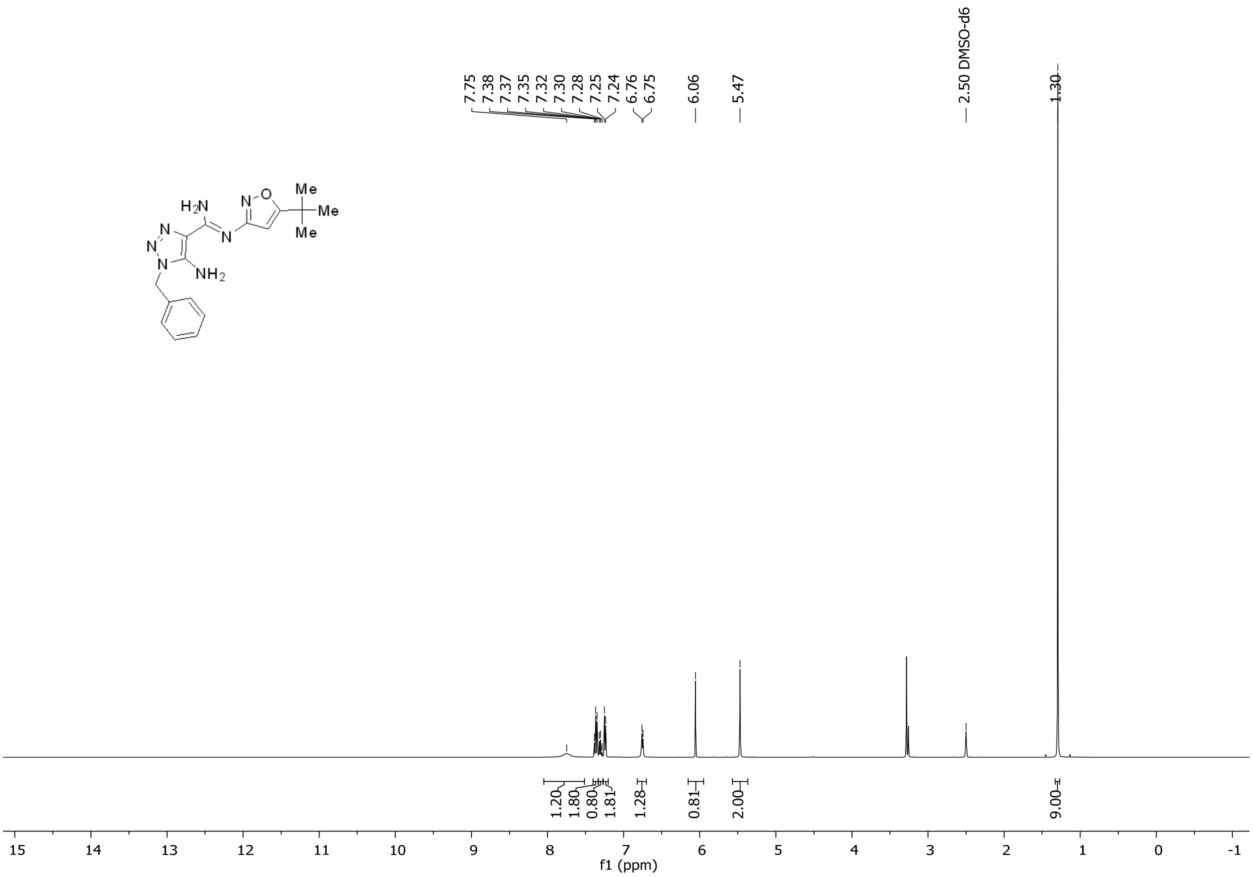

Fig. S38. ^{13}C NMR spectrum (DMSO-d_6) of **3r**

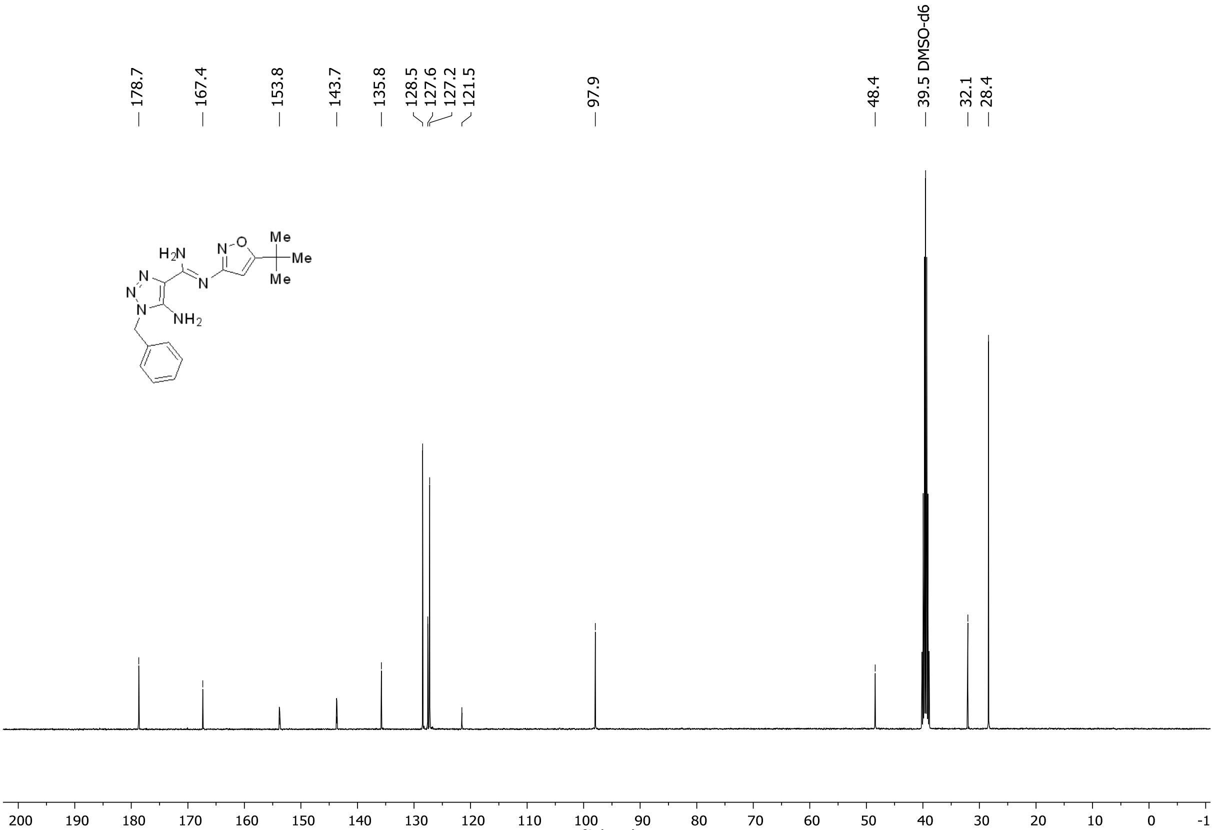

Fig. 38A. HMBC spectrum (DMSO-d$_6$) of **3r**

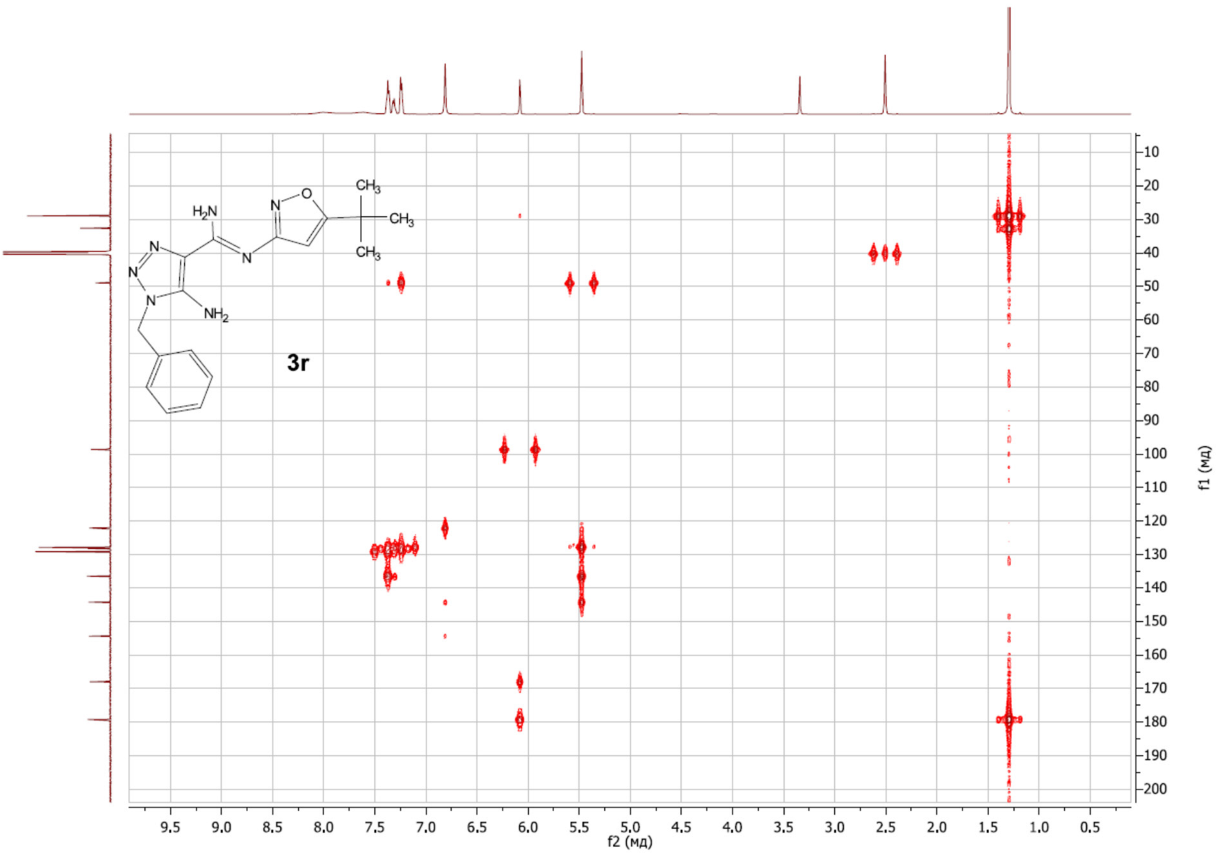

Fig. S39. ^{1}H NMR spectrum (DMSO-*d*$_6$) of **3s**

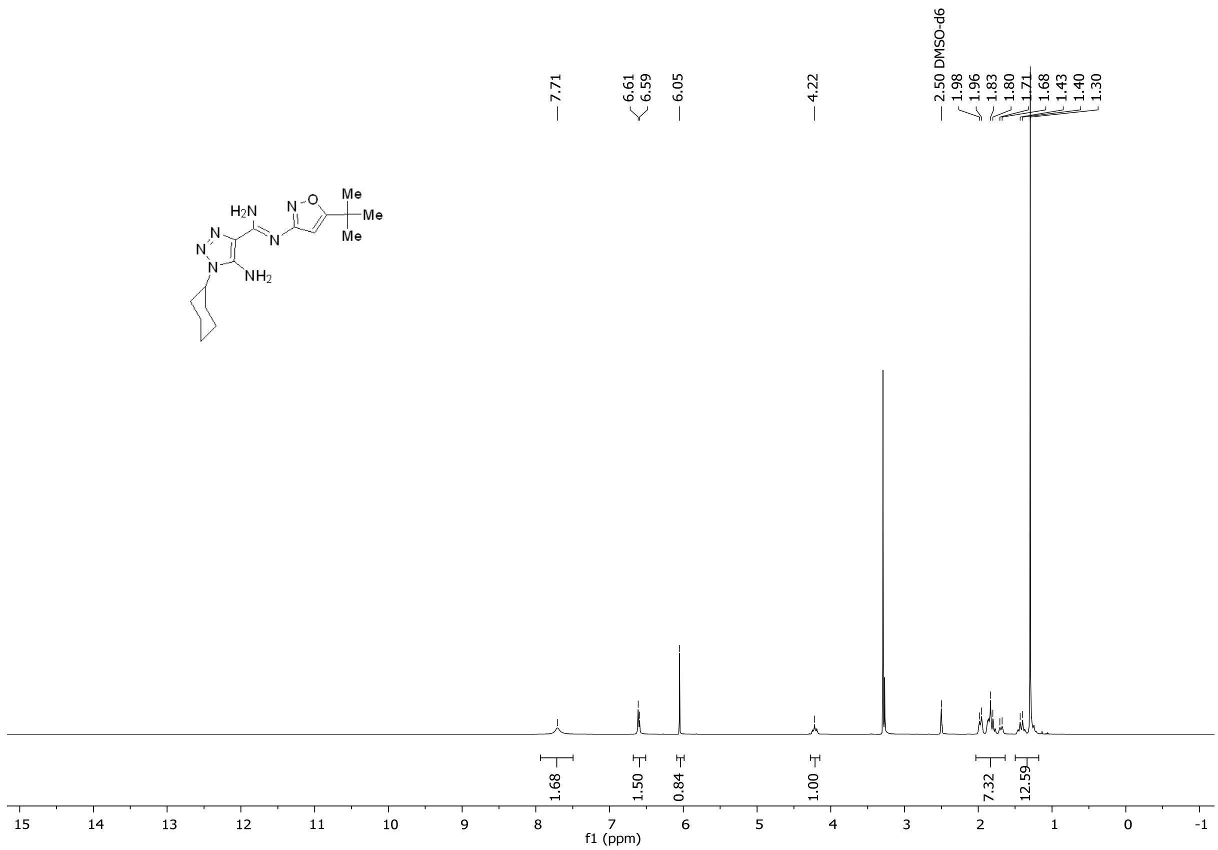

Fig. S40. ^{13}C NMR spectrum (DMSO-*d*$_6$) of **3s**

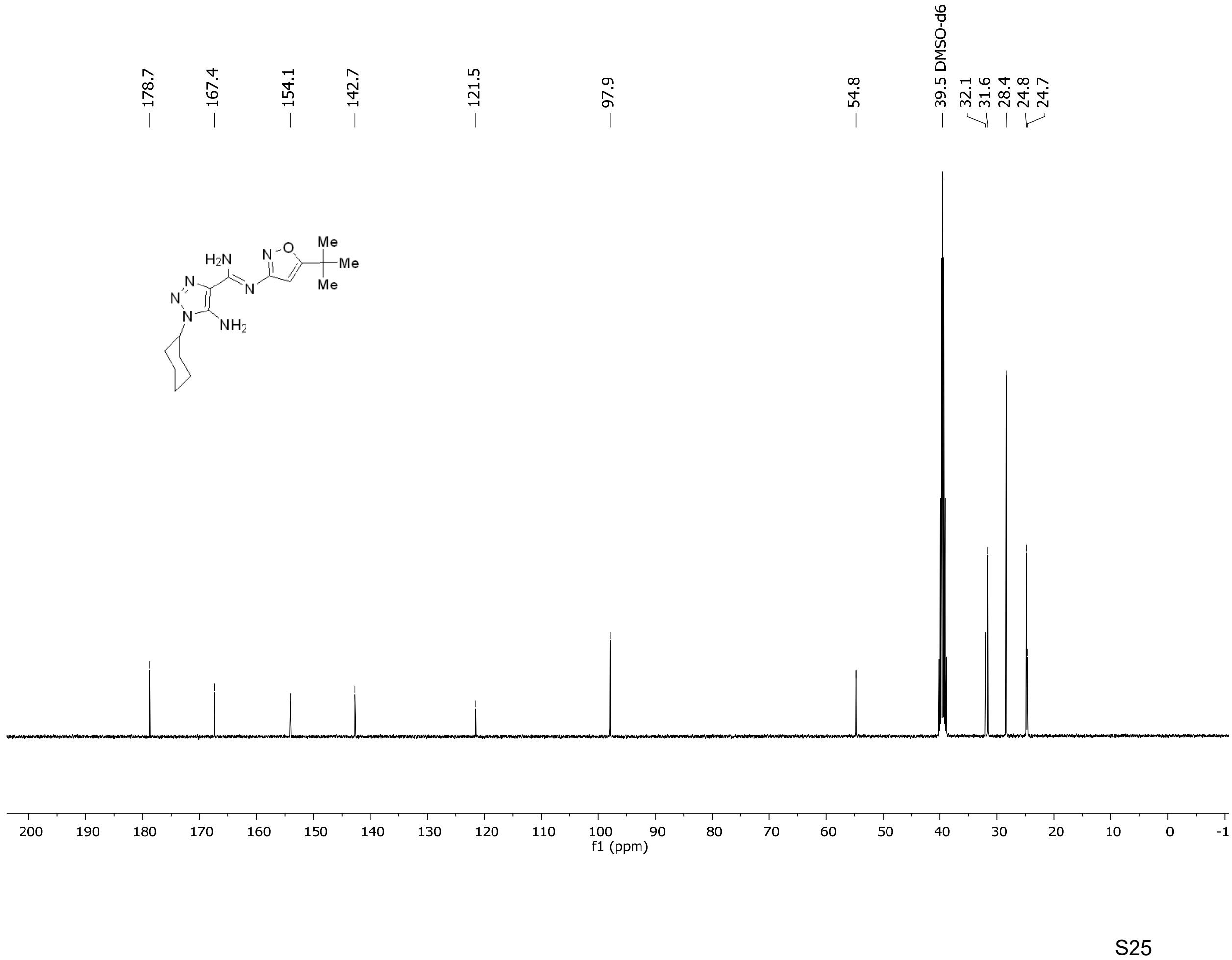

Fig. S41. ^{1}H NMR spectrum (DMSO-d_6) of **3t**

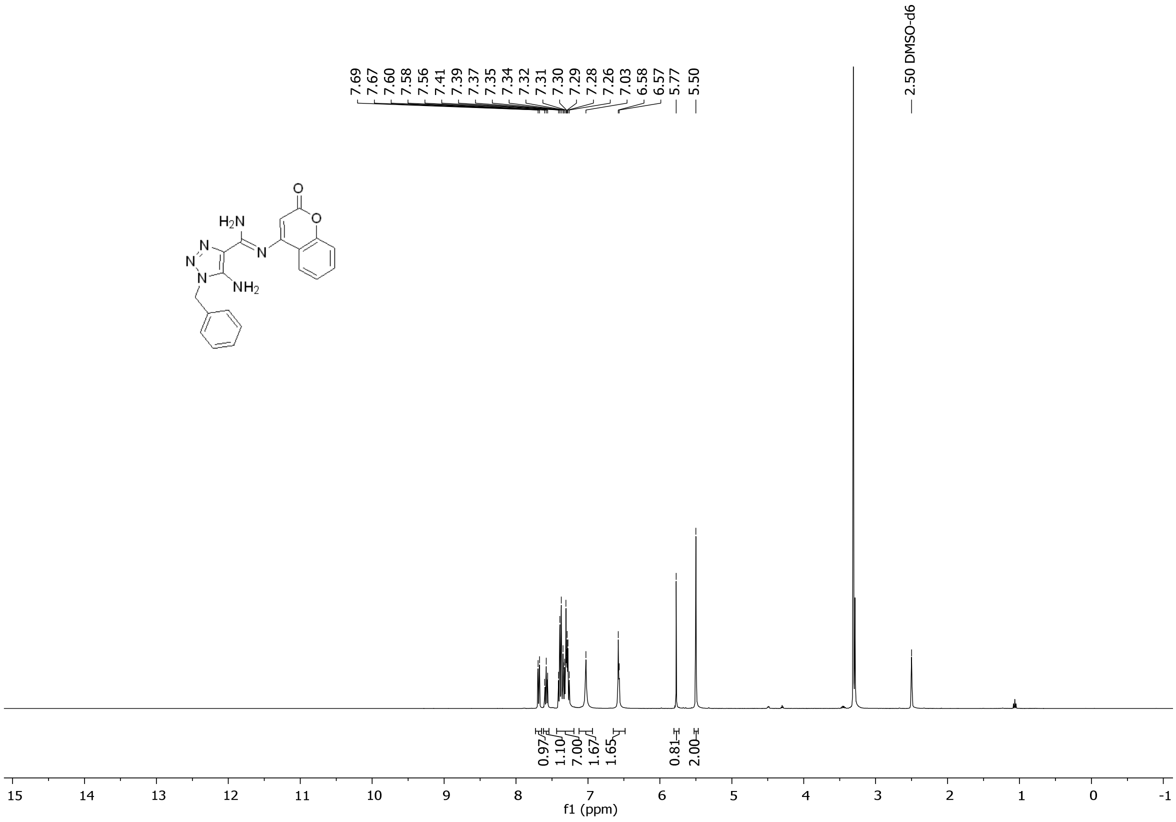

Fig. S42. ^{13}C NMR spectrum (DMSO-d_6) of **3t**

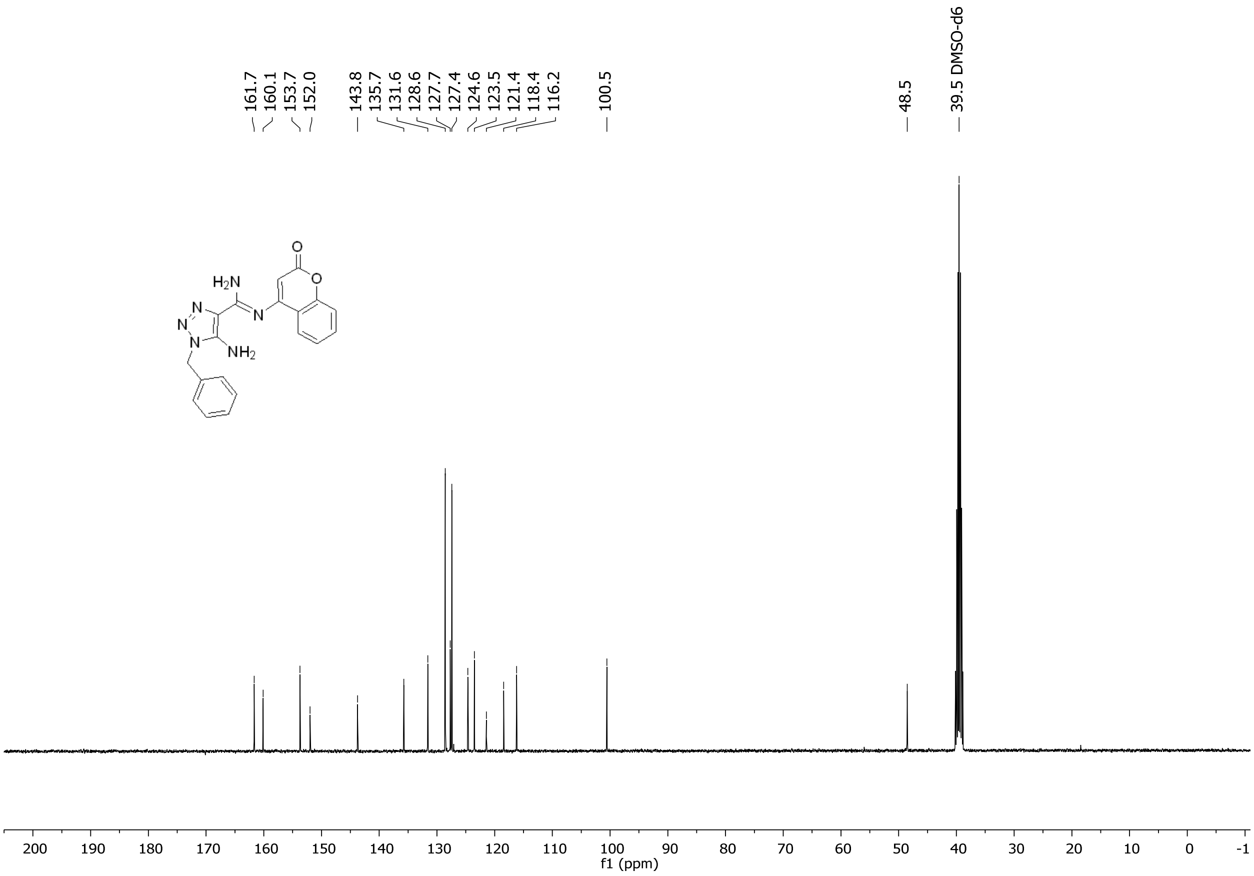

Fig. S43. ^{1}H NMR spectrum (DMSO-d_6) of **3u**

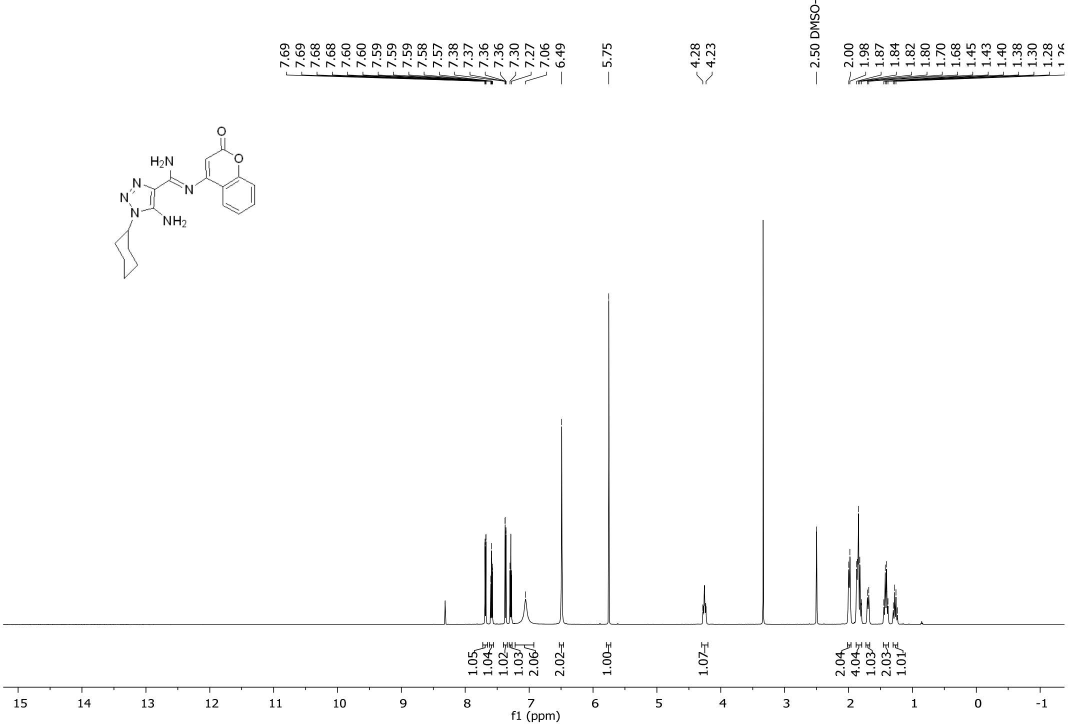

Fig. S44. ^{13}C NMR spectrum (DMSO-d_6) of **3u**

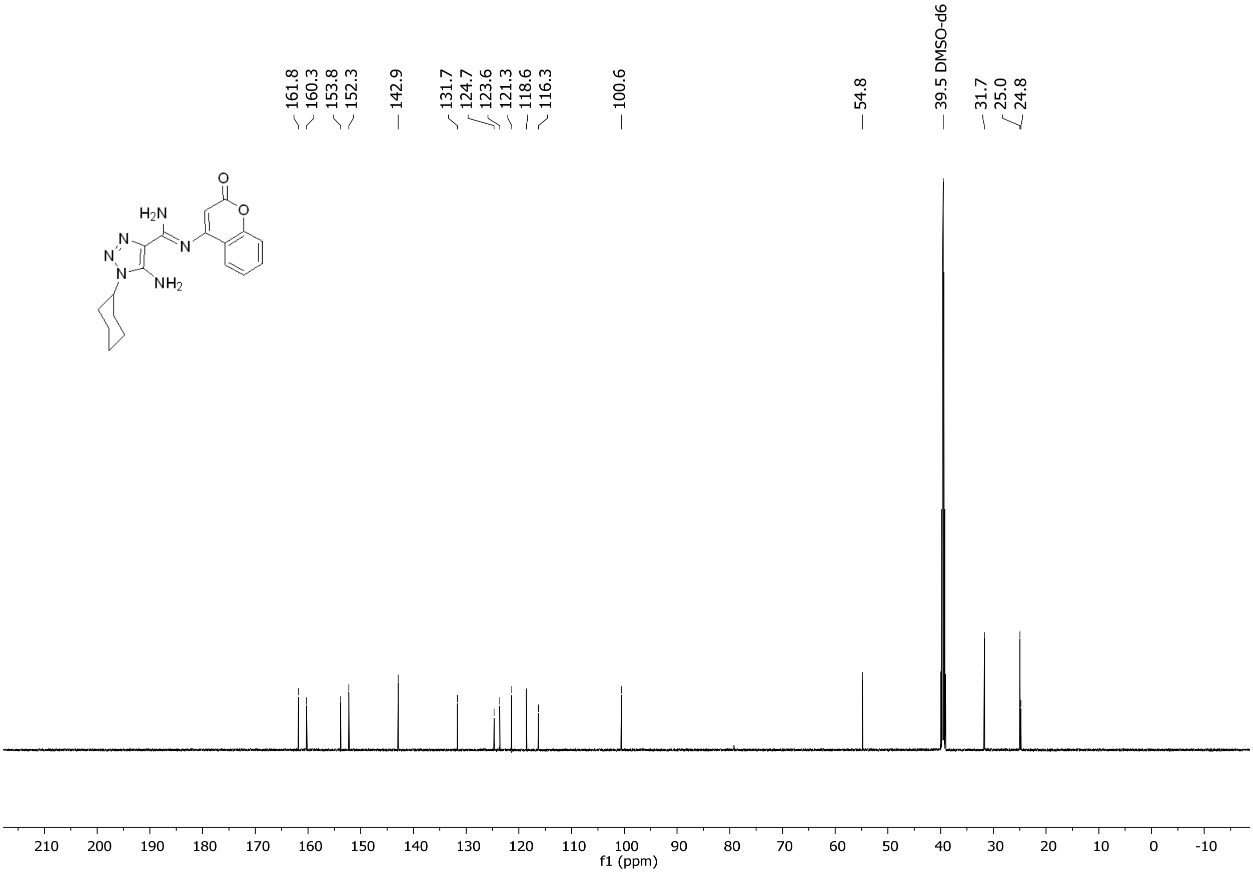

X-ray Single Crystal Data for 3g (CCDC: 2298850)

Procedure for the sample preparation: Compound **3g** (20 mg) was dissolved in EtOH. After slow evaporation at room temperature, the single crystals were afforded.

Fig. S45. Crystal Structure of **3g** (50% probability level)

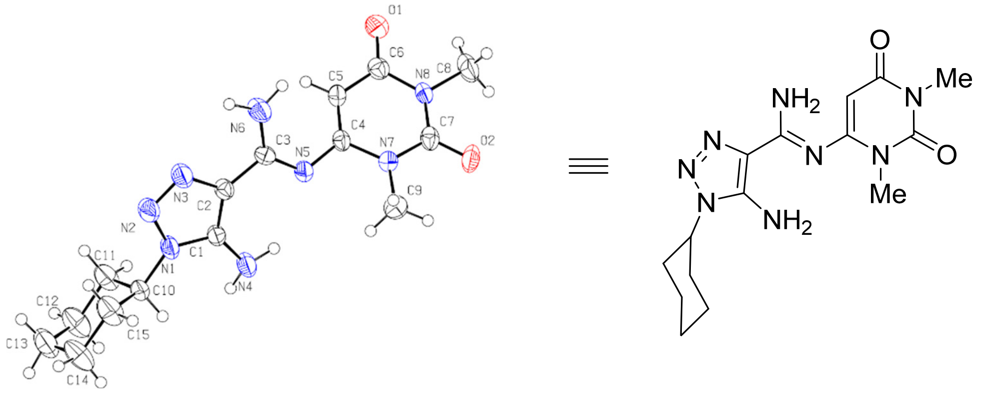

Chemical formula	$C_{15}H_{22}N_8O_2$
Formula weight	346.40
Temperature/K	295
Crystal system	Monoclinic
Space group	$P2_1/c$
a/Å	14.734(3)
b/Å	9.000(2)
c/Å	13.125(4)
α/°	90
β/°	104.29
γ/°	90
Volume/Å^3	1686.6(8)
Z	4
Radiation type	Mo $K\alpha$
Dx/Mg m^{-3}	1.364
μ/mm^{-1}	0.10
F(000)	736
Crystal size/mm	0.45 × 0.3 × 0.04
θ_{max}, θ_{min}/°	29.5, 2.7
Index ranges	$-20 \le h \le 20$, $-11 \le k \le 11$, $-17 \le l \le 17$
Measured reflections	6415
Independent reflections	6415
Reflections with $I > 2\sigma(I)$	3101
Reflections/parameters/restraints	6415/244/0
Goodness-of-fit	0.87
Final R indexes [$F^2 > 2\sigma(F^2)$]	$R_1 = 0.069$, $wR_2 = 0.185$
Final R indexes [all data]	$R_1 = 0.135$, $wR_2 = 0.207$
$\Delta\rho_{max}$, $\Delta\rho_{min}$/e Å^{-3}	0.31, -0.35
